# Effectiveness of a Timing and Coordination Group Exercise Program to Improve Mobility in Community-Dwelling Older Adults: A Randomized Clinical Trial

**DOI:** 10.1001/jamainternmed.2017.3609

**Published:** 2017-10-02

**Authors:** Jennifer S. Brach, Subashan Perera, Sandra Gilmore, Jessie M. VanSwearingen, Deborah Brodine, Neelesh K. Nadkarni, Edmund Ricci

**Affiliations:** 1 Department of Physical Therapy, University of Pittsburgh, Pittsburgh, Pennsylvania; 2 Division of Geriatric Medicine, Department of Medicine, University of Pittsburgh, Pittsburgh, Pennsylvania; 3 Department of Biostatistics, University of Pittsburgh, Pittsburgh, Pennsylvania; 4 Community Provider Services, University of Pittsburgh Medical Center, Pittsburgh, Pennsylvania; 5 Department of Behavioral and Community Health Sciences, University of Pittsburgh, Pittsburgh, Pennsylvania

## Abstract

**Importance:**

Timing and coordination exercises may be an important addition to community-based health promotion exercise programs to improve walking in older adults.

**Objective:**

To compare the effectiveness of the On the Move group exercise program, which focuses on the timing and coordination of movement, with a seated strength, endurance, and flexibility program (usual care) at improving function, disability, and walking ability of older adults.

**Design, Setting, and Participants:**

Cluster-randomized, single-blind intervention trial. Thirty-two independent living facilities, senior apartment buildings, and senior community centers were randomized to On the Move (16 sites; 152 participants) or usual care (16 sites; 146 participants). Participants were 65 years or older, able to ambulate independently with a gait speed of at least 0.60 m/s, able to follow 2-step commands, and were medically stable.

**Interventions:**

Exercise classes were 50 minutes, twice a week for 12 weeks and had 10 or fewer participants per class. On the Move consisted of warm-up, timing and coordination (stepping and walking patterns), strengthening, and stretching exercises. The usual-care program consisted of warm-up, strength, endurance, and stretching exercises.

**Main Outcomes and Measures:**

The primary outcomes were self-report of function and disability (Late Life Function and Disability Instrument) and mobility (6-minute walk distance and gait speed) assessed by blinded individuals.

**Results:**

Participants (mean [SD] age, 80.0 [8.1] years) were mostly female (251 [84.2%]) and white (249 [83.6%]) and had a mean (SD) of 2.8 (1.4) chronic conditions. Intervention groups were similar on baseline characteristics. Postintervention, 142 (93.4%) participants in On the Move and 139 (95.2%) participants in usual care completed testing. On the Move had greater mean (SD) improvements than the usual-care group in gait speed (0.05 [0.13] vs −0.01 [0.11] m/s; adjusted difference = 0.05 [0.02] m/s; *P* = .002) and 6-minute walk distance (20.6 [57.1] vs 4.1 [55.6] m; adjusted difference = 16.7 [7.4] m; *P* = .03). Attendance was greater in the usual-care program compared with On the Move (95 [65.1%] vs 76 [50.0%] attended ≥20 classes; *P* = .03). There were no significant differences in any of the other primary or secondary outcomes.

**Conclusions and Relevance:**

The On the Move group exercise program was more effective at improving mobility than a usual-care exercise program, despite lower attendance. Additional research examining the impact of the intervention on long-term disability outcomes is needed before recommending routine implementation into clinical practice.

**Trial Registration:**

clinicaltrials.gov Identifier: NCT01986647

## Key Points

**Question:**

What is the effectiveness of a timing and coordination group exercise program (On the Move) compared with a seated strength, endurance, and flexibility exercise program (usual care) for improving mobility in community-dwelling older adults?

**Findings:**

In this single-blind cluster-randomized trial that included 298 older adults, participants in the On the Move group had greater improvements in mobility than those in the usual-care group.

**Meaning:**

Group exercise programs to improve mobility in older adults should include timing and coordination exercises that are important for walking.

## Teaser

This cluster-randomized trial compares the effectiveness of a group exercise program that focuses on the timing and coordination of movement with a seated strength, endurance, and flexibility program (usual care) on function, disability, and walking ability of older adults.

## Introduction

Walking difficulty is a common, costly condition in older adults.^1^ Walking difficulty contributes to loss of independence, higher rates of morbidity, and increased mortality.^2^ Exercise is beneficial to physical and mental health and may prevent walking difficulty.^4^ Community-based group exercise programs are 1 option for promoting health and wellness and could potentially be used to improve walking in older adults.

Studies examining the impact of group exercise programs on walking have conflicting findings and limitations.^7^ Many of the studies were small, conducted in “young” older adults, or the control groups were nonexercise. The 1 group exercise program that did improve mobility consisted of a high dose of exercise (65 minutes a day, 5 days a week for 24 weeks), which may not be acceptable to most older adults.^7^ Whereas many group programs emphasize lower extremity muscle strengthening, flexibility, and general conditioning because of the association of related impairments with walking difficulties,^12^ they have failed to focus on the ability to walk or the timing and coordination of movement that is critical to walking.^7^ Previously, therapeutic, individually supervised exercise led by physical therapists that includes timing and coordination components has been shown to improve walking in older adults.^13^ Therefore, timing and coordination exercises could be an important addition to community-based health promotion group exercise programs to improve walking.

Based on previous research,^14^ and with critical input from older adults and other stakeholders, the On the Move (OTM) group-based exercise program that includes timing and coordination and focuses on improving walking was developed.^17^ A pilot study showed that OTM was feasible and acceptable.^17^ We now report the results of a large patient-centered comparative effectiveness trial establishing the effectiveness of OTM against a usual-care group exercise program in community-dwelling older adults residing in independent living facilities, senior apartment buildings, and attending senior community centers. We hypothesized that OTM would produce greater gains in self-reported function and disability and walking ability, be acceptable and low in risk, and would result in greater satisfaction and adherence than the usual-care group exercise program.

## Methods

### Study Design and Oversight

Detailed methods are published elsewhere.^18^ As described therein, all major components of the study design including formulation of hypotheses, participants, randomization, and outcomes were influenced by input from stakeholders and decided on with a patient-centered view. Briefly, the trial was designed to establish the effectiveness and explore the sustainability of the OTM group exercise program. The study was a cluster-randomized, single-blind intervention trial to compare the effects of OTM and a usual-care group exercise program in community-dwelling older adults. The sustainability of the program, defined as similar benefits when taught by a community member, is being explored elsewhere (D. Wert, PhD, PT, et al, unpublished data, May 2017). The study was approved by the University of Pittsburgh Institutional Review Board and registered in clinicaltrials.gov, and signed informed consent was obtained from all participants. The study protocol is available in Supplement 1.

### Study Participants

Participants were recruited between April 2014 and January 2016, from those individuals residing in independent living facilities and senior housing, and attending community centers in the greater Pittsburgh, Pennsylvania, area. Inclusion criteria were age 65 years or older, a resident or member of the participating facility, and ability to ambulate independently (with or without a straight cane) with a gait speed of at least 0.60 m/s. Individuals with a gait speed less than 0.60 m/s have difficulty participating in the program and raise safety concerns in the group exercise setting. Those non-English speaking; unable to follow a 2-step command or understand the informed consent process; planning to leave the area for an extended period of time; with a progressive neuromuscular disorder; with any medical condition or illness that was not stable (ie, unplanned hospitalization for a life-threatening illness or major surgery in the past 6 months); or with a post–6-minute walk test heart rate of at least 120 beats per minute, systolic blood pressure of at least 220 mm Hg, a drop in systolic blood pressure of greater than 10 mm Hg, or diastolic blood pressure of at least 110 mm Hg were excluded.

### Interventions

Both exercise programs were group based, had identical frequency and duration (50 minutes, twice a week for 12 weeks), had 10 or fewer participants per class, and were delivered by trained exercise leaders (ie, physical therapists or physical therapy assistants). All exercise sessions were held on site at the facilities and were initiated within 2 weeks of baseline testing. Exercise program fidelity was determined through site visits by the program developers. A detailed description of both interventions can be found in the eMethods in Supplement 2.

On the Move was based on principles of motor learning that enhance “skill,” or smooth and automatic movement control.^19^ The program contained a warm-up, stepping patterns, walking patterns, strengthening, and cool-down.^22^ The stepping and walking patterns were goal-oriented, progressively harder patterns that promoted the timing and coordination of stepping, integrated with the phases of the gait cycle.^20^^(pp181-194)^ ^21^ The goal of the stepping patterns was to facilitate a shifting of the center of pressure posterolateral and then forward, encouraging hip extension. Stepping patterns consisted of stepping forward and across the midline of the body with 1 foot for several repetitions followed by stepping forward and across the midline with the opposite foot. A similar activity was conducted with backward stepping. Stepping was progressed from stepping on all 1 side, to alternating left and right steps, to alternating forward and backward stepping. The goal of the walking patterns was to promote a shift of the center of pressure during medial stance and to promote the timing and interlimb coordination of muscle activations (ie, abductors of the imminent swing limb with the adductors of the stance limb). Patterns consisted of ovals, spirals, and serpentines that were progressed by changing the amplitude of the pattern (ie, narrower oval), altering the speed of walking, or increasing the complexity of the task (ie, walking past other walkers or object manipulation while walking). Exercises were progressed when participants correctly completed the activity at least 80% of the time. Only 1 item—amplitude, speed, or complexity—was progressed at a time. The strengthening program focused on lower extremity muscle groups important for walking. A detailed description of OTM can be found in Brach et al.^17^ The majority of the program was conducted in standing (40 minutes), with only 10 minutes in sitting.

The usual-care group exercise comparator was a seated strength, endurance, and flexibility program based on programs being conducted in the community-based facilities. The usual-care exercise program contained a warm-up, upper and lower extremity strength exercises, aerobic activities, and a cool-down. The warm-up and cool-down contained gentle range-of-motion exercises and stretches. Strengthening exercises focused on upper and lower extremity muscle groups and used playground balls, the opposite extremity, and body weight to provide resistance to the movements. Aerobic activities included repeated movements of the lower extremities (marching, tapping, and skiing) at various speeds. Upper extremity movements were added to increase the intensity of the activity. The entire program was conducted while sitting (50 minutes).

### Randomization

We randomized facilities equally to the 2 interventions stratified by facility type. Participating independent-living facilities were known ahead of time and further stratified by socioeconomic status, academic medical center affiliation, and location in adjacent county before randomization to achieve a balance by design. Each of the other 2 facility types was randomized as they agreed to participate, using random block sizes of 2 and 4. Facility assignment was revealed to the coordinator only after baseline testing. We then randomized participants within each facility to a class run by a study exercise leader (primary aim) or a subsequent one by a facility staff activity person (exploratory sustainability aim). Per a midtrial protocol modification approved by the sponsor for potential safety concerns, if a suitable facility staff person was not available, their class was also taught by an exercise leader, and participants were analyzed accordingly.

### Outcomes

All measures were collected at baseline prior to randomization, immediately prior to intervention in those randomized to a subsequent class, and immediately following the 12-week intervention by blinded personnel. All testing was conducted on site at the facilities.

The primary outcomes were Late Life Function and Disability Instrument (LLFDI) overall function and disability frequency domains, 6-minute walk distance (6MWD), and gait speed. The LLFDI is a pair of self-report instruments targeted for assessing physical function and disability in older adults with acute or chronic problems, and designed to be more sensitive to change than similar measures. The LLFDI has established known-groups validity and test-retest reliability (0.68-0.98).^24^ Scores range from 0 to 100, with higher scores representing greater function. The 6MWD included time for rest as needed^26^ and has excellent test-retest reliability (0.95)^27^ and construct validity.^28^ A 20-m change in 6MWD is considered small but meaningful, and 50 m, substantial.^29^ Gait speed is a strong indicator and predictor of disability, morbidity, and mortality,^2^ and was assessed on an instrumented walkway (Zeno Walkway, Zenometrics). Participants completed 6 passes at their usual speed, which were averaged. Gait speed has excellent test-retest reliability (0.98).^31^ A 0.05-m/s change in gait speed is considered small but meaningful, and 0.10 m/s, substantial.^29^ The secondary outcomes were Figure of 8 walk test, modified Gait Efficacy Scale, Short Physical Performance Battery, gait variability, and complex walks.^32^ Demographic characteristics, fear of falling, fall history, height, and weight were self-reported. Chronic condition burden was assessed with a self-reported comorbidity index indicating 18 common conditions. Eight domains (cardiovascular, respiratory, musculoskeletal, neurological, general, cancer, diabetes, and visual) were derived and summed.^36^

Participant satisfaction was assessed by exit surveys. The items included degree of satisfaction with components of the exercise program (the exercises, safety, individualized instruction, satisfaction, and likelihood of continued participation), participants’ expectations, perception of benefit from the program, and likelihood of recommending the program to others, and included open-ended questions.

Adherence was measured by class attendance rosters. Reasons for missed classes were recorded when available. Adverse events during testing or intervention were recorded for review by the study physician for adjudication and direction.

### Sample Size

We based sample size on pilot studies,^13^ 2-tailed α = .05 tests, 80% power, 10% attrition, class size of 10, intracluster correlation of 0.1, and detectable clinically meaningful^29^or moderate effect sizes (Cohen *d* = 0.5).^37^ Ninety participants per arm was estimated to detect a between-intervention difference as small as 3.1 points in LLFDI overall function change; 80 per arm for 3.2 points in LLFDI disability frequency; 140 per arm for 0.1 m/s in gait speed; and 40 per arm for 50 m in 6MWD. Therefore, 140 per intervention were necessary in arms taught by exercise leaders to accommodate all primary outcomes.

### Statistical Analysis

We performed a prespecified intention-to-treat analysis. Participant flow was summarized using a CONSORT diagram.^38^ First, the baseline participant characteristics were compared between the 2 arms. Second, we performed a multivariate Hotelling *t* test to simultaneously compare the baseline to follow-up change in the primary outcomes between arms to protect the type I error rate from multiplicity. On observing significance, subsequent analyses were to be performed without further multiplicity adjustment. Third, we fit linear mixed models^39^^(pp275-283)^ with baseline to follow-up change in continuous outcomes as the dependent variable; intervention arm as the fixed effect of interest; baseline value of outcome as a covariate; and a facility random effect for site clustering. We used multiple imputation to account for missing data.^40^^(p15-18)^ Fourth, dichotomous secondary outcomes were analyzed similarly, but using a generalized estimating equations model^41^^(pp146-147)^ with a binomial distribution, logit link, and an exchangeable correlation structure for clustering. Fifth, we explored with a series of unplanned subgroup analyses stratified by adherence, setting, and baseline performance and/or function. Finally, we performed sensitivity analyses of results to additionally adjust for baseline characteristics different between groups, using immediate preintervention measurement instead of prerandomization baseline for those in a subsequent class, and ignoring missing data. SAS, version 9.3, software (SAS Institute, Inc) was used.

## Results

See the Figure for participant flow and sources of missing data (and eFigure, A and B in Supplement 2 for a comprehensive CONSORT diagram of the entire trial). Of the 37 sites contacted, 32 participated. Three were not interested, and 2 had insufficient numbers of participants. By telephone or in person, 560 were screened for initial eligibility, among whom 482 were eligible and assessed in person (17 failed: 5 used a walker; 5 too young; 7 for other reasons). Of them, 476 completed screening (6 did not: 3 refused to sign the liability waiver; 3 changed mind). Of the 52 screen failures, 37 had gait speed less than 0.6 m/s and 15 had abnormal blood pressure and/or heart rate, leaving 424 for trial participation. Of the 32 sites, 16 were randomized to OTM and 16 to usual-care intervention where exercise leaders taught 152 and 146 participants, respectively. In OTM, 142 (93.4%) completed postintervention testing and 10 did not (4 lost to follow-up, 5 health issues, 1 dropped out). In the usual-care program, 139 (95.2%) completed postintervention testing and 7 did not (1 lost to follow-up, 3 health issues, 3 dropped out).

**Figure.**
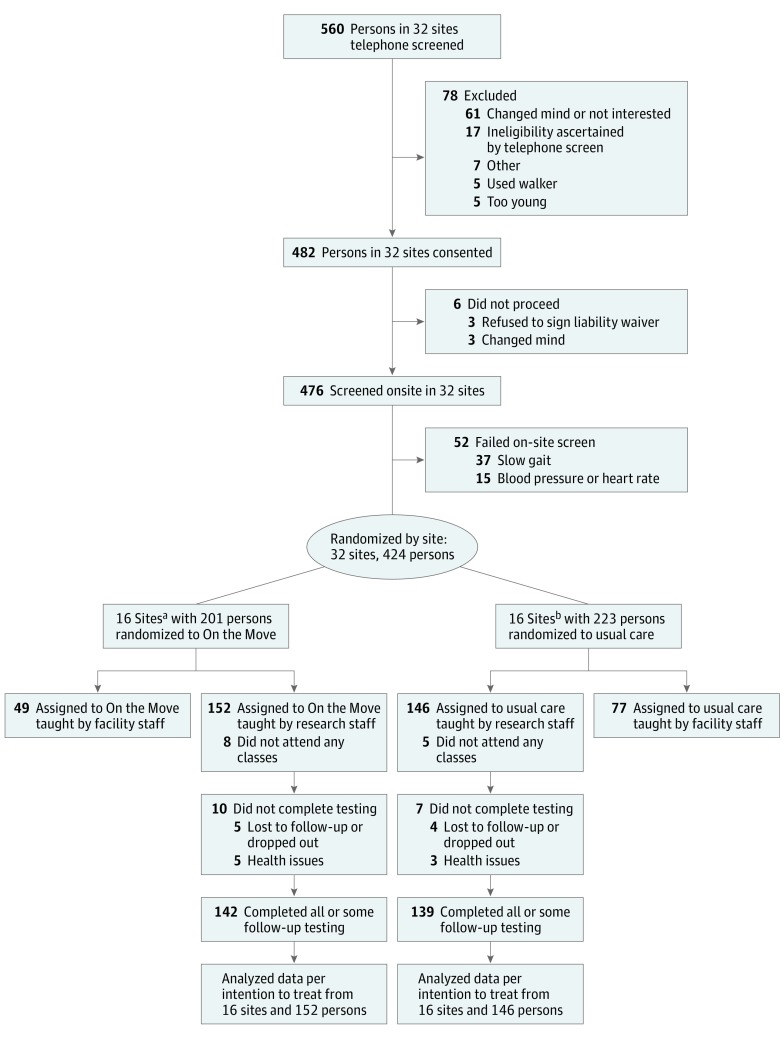
CONSORT Diagram ^a^Sites included 5 independent living facilities, 3 senior community centers, and 8 senior apartment buildings. ^b^Sites included 5 independent living facilities, 4 senior community centers, and 7 senior apartment buildings.

Participants had a mean (SD) age of 80.0 (8.1) years, were mostly female (84.2%) and white (83.6%), and had a mean (SD) of 2.8 (1.4) chronic conditions. Participants walked slowly (mean [SD] speed, 0.91 [0.21] m/s), and 170 (57.0%) had a 6MWD less than community ambulation distance (300 m). The intervention groups were similar except minor differences in facility type (Table 1).

**Table 1.** Participant Characteristics and Measures at Baseline by Intervention Group

Characteristic	On the Move (n = 152)	Usual Care (n = 146)	*P* Value^a^
Recruitment and intervention setting, No. (%)^b^			
Community senior center	36 (23.7)	29 (19.9)	.04
Independent living facility	42 (27.6)	61 (41.8)	
Senior apartment complex	74 (48.7)	56 (38.4)	
Age, mean (SD), y	79.6 (8.2)	80.5 (8.1)	.74
Female sex, No. (%)	131 (86.2)	120 (82.2)	.55
White race, No. (%)	129 (84.9)	120 (82.2)	.62
Married, No. (%)	29 (19.1)	30 (20.6)	.88
College education, No. (%)	67 (44.1)	73 (50.7)	.77
Comorbidities, No. (%)			
Cardiovascular	26 (17.1)	26 (17.8)	.87
Neurological	10 (6.6)	13 (8.9)	.36
Musculoskeletal	129 (84.9)	114 (78.1)	.18
General	66 (43.4)	56 (38.4)	.33
Visual and/or hearing	109 (71.7)	107 (71.9)	.92
Diabetes	41 (27.0)	25 (17.1)	.06
Cancer	27 (17.8)	28 (19.2)	.74
Lung	30 (19.7)	32 (21.9)	.70
Duke comorbidity index, mean (SD)	2.9 (1.4)	2.7 (1.5)	.45
Fear of falling, No. (%)	53 (34.9)	57 (39.0)	.53
Fall prior year, No. (%)	45 (29.6)	41 (28.1)	.85
Excellent/very good mobility, No. (%)	90 (59.2)	90 (61.6)	.63
Excellent/very good health, No. (%)	80 (52.6)	75 (51.4)	.85
Excellent/very good balance, No. (%)	47 (30.9)	51 (34.9)	.42
Height, mean (SD), m	1.61 (0.10)	1.61 (0.13)	.97
Weight, mean (SD), kg	75.3 (21.1)	71.9 (15.7)	.35
BMI, mean (SD)	29.0 (7.6)	28.6 (15.8)	.86
Six-minute walk distance, mean (SD), m	273.3 (88.0)	277.3 (95.5)	.77
<300 m, No. (%)	89 (58.6)	81 (55.5)	
≥300 m, No. (%)	63 (41.5)	65 (44.5)	
Late Life Function and Disability Index, mean (SD)			
Overall function	58.9 (8.5)	60.0 (10.5)	.34
Disability frequency	53.3 (6.5)	51.6 (5.8)	.24
Instrumented walkway gait speed, mean (SD), m/s	0.90 (0.20)	0.92 (0.21)	.28
<0.8 m/s, No. (%)	52 (34.9)	35 (25.7)	
0.8 to <1.0 m/s, No. (%)	52 (34.9)	56 (41.2)	
≥1.0 m/s, No. (%)	45 (30.2)	45 (33.1)	

Abbreviation: BMI, body mass index (calculated as weight in kilograms divided by height in meters squared).

^a^ Obtained using a linear mixed or generalized estimating equation model due to clustering by facility unless otherwise noted.

^b^ *P* value obtained using χ^2^ test.

The 2 groups had significantly different improvements, when primary outcomes were simultaneously considered in a multivariate test (*P* = .02). The OTM group had significantly greater mean (SD) improvements than the usual-care group in gait speed (0.05 [0.13] vs −0.01 [0.11] m/s; adjusted difference = 0.05 [0.02] m/s; *P* = .002) and 6MWD (20.6 [57.1] vs 4.1 [55.6] m; adjusted difference = 16.7 [7.4] m; *P* = .03). A meaningful improvement in gait speed (0.05 m/s) was achieved by 64 of 152 (42.1%) and 36 of 146 (24.7%), respectively, in the 2 groups, resulting in a number needed to treat of 5.7. There were no significant differences between groups in other outcomes (Table 2 and Table 3).

**Table 2.** Baseline to Follow-up Change in Primary Outcome Measures and Between-Intervention Differences

Measure	Baseline to Follow-up Change Estimate (SE)		Adjusted Difference (SE)^a^	*P* Value
	On the Move	Usual Care		
LLFDI overall function	0.4 (5.7)	−0.6 (5.8)	0.8 (0.7)	.27
LLFDI disability frequency	0.4 (4.1)	0.7 (5.0)	0.3 (0.6)	.61
Six-minute walk distance, m	20.6 (57.1)	4.1 (55.6)	16.7 (7.4)	.03
Instrumented walkway gait speed, m/s	0.05 (0.13)	−0.01 (0.11)	0.05 (0.02)	.002

Abbreviation: LLFDI, Late-Life Function and Disability Index.

^a^ Obtained using a linear mixed model due to clustering by facility and multiple imputation for missing data.

**Table 3.** Comparisons of Follow-up Measures of Exercise Attendance and Satisfaction^a^

Measure	No. (%)		Odds Ratio (95% CI)	*P* Value
	On the Move (n = 152)	Usual Care (n = 146)		
Attended ≥20 classes	76 (50.0)	95 (65.1)	0.52 (0.29-0.95)	.03
Satisfaction				
Benefited from class a good bit/somewhat	104 (78.8)	105 (80.8)	0.89 (0.49-1.60)	.69
Class was at least somewhat challenging	92 (69.7)	94 (72.3)	0.88 (0.56-1.37)	.57
Just right or more individualized instruction	128 (97.0)	124 (95.4)	1.56 (0.44-5.53)	.49
Felt safe or very safe	131 (99.2)	127 (97.7)	3.12 (0.37-26.6)	.30
Satisfied or very satisfied	128 (97.0)	126 (96.9)	1.06 (0.30-3.74)	.93
Definitely or probably will continue exercise afterward	113 (85.6)	108 (83.1)	1.17 (0.67-2.06)	.58

^a^ Using a generalized estimating equations model to account for clustering by site.

Participants in OTM were less likely than usual-care program participants to attend at least 20 of the 24 classes (76 [50.0%] vs 95 [65.1%]; odds ratio, 0.52; 95% CI, 0.29-0.95; *P* = .03) (Table 3). The majorities in both programs felt that they benefited from the class; the classes were at least somewhat challenging; they received just enough or more personalized instruction; and felt safe or very safe while doing the exercises. In both groups, almost all (128 [97.0%] and 126 [96.9%]) were satisfied or very satisfied and said that they would continue with the same program if offered (113 [85.6%] vs 108 [83.1%]).

Unplanned exploratory subgroup analyses showed significant treatment by class attendance (*P* = .01) and facility type (*P* = .04) interactions with respect to 6MWD. We hypothesize that those attending 20 or more classes (mean [SE] 6MWD improvement, 31.8 [9.4] m; *P* = .002) or from community centers (mean [SE], 48.9 [17.3] m; *P* = .005) may derive greater benefits from OTM than the usual-care program. There were 4 adverse events (falls, fatigue, pain), and all occurred during the OTM classes. Sensitivity analyses did not materially change the results (data not shown).

## Discussion

The On the Move program, designed to target the timing and coordination of movement important for walking, elicited greater improvements in mobility than a usual-care seated group exercise program when taught by an exercise leader. The greater gait speed gain was both statistically significant and clinically meaningful based on the 0.05-m/s criterion, with a favorable 5.7 needed to treat.^29^ The greater 6MWD gain was close to the small but meaningful change criterion of 20 m.^29^ The greater improvements in mobility in OTM compared with the usual-care group occurred despite the lower adherence. Our findings support the idea that timing and coordination exercise should be included in group exercise programs to improve mobility in older adults. Individuals in OTM did not report greater improvements in function and disability than the usual-care group. One possible explanation is that mobility performance improvement may not have reached a threshold that the older adults would recognize as affecting overall function and disability. Another possible explanation is that the intervention may need to be of a longer duration to affect function and disability.

Although the number of adverse events experienced during the trial was minimal (4 events), it is important that all the events occurred during the OTM program. They were expected adverse events of exercise and did not prohibit anyone from finishing the program. All who experienced an event were willing and able to return to exercise. Previously, we had found that older adults recognize the risks of more challenging exercises yet believe that the risks are worth the benefit.^17^

Attendance in OTM was lower than in the usual-care exercise program. Participants were equally satisfied with either program, and they felt safe in both programs. On the Move is conducted primarily while standing and was designed to be a more challenging program than the usual-care class. One potential explanation for the lower adherence is that an individual who had a health status change during the 12-week program may have been more likely to continue in the usual-care program, which was completed while seated, than in the OTM program, which was completed primarily while standing. Another potential explanation is that participants need a certain level of baseline reserve to elicit greater gains from OTM than the usual-care program, as suggested by weak evidence of greater between-intervention differences among faster walkers than slower walkers from the post hoc subgroup analyses.

Our findings have important public health relevance. Many community sites are looking for evidence-based exercise programs for their facilities. By examining the OTM intervention in a rigorous comparative effectiveness trial, OTM can be submitted to the Administration for Community Living to be evaluated for Evidence-Based Program status. The purpose of this process is to improve access to information on evidence-based interventions with the ultimate goal of reducing the lag time between the creation of scientific knowledge and its practical application in the field. Interventions that have the Evidence-Based Program designation have met the highest-level criteria for Title IIID funding of the Older Americans Act. Obtaining the Evidence-Based Program designation is an important first step in the translation of OTM into widespread use in the community. 

Our study has several strengths. First, ours was a real-world community-based comparative effectiveness study^42^ in which we compared OTM with a usual-care exercise program instead of a passive nonintervention control. Many of the previous research reports on community-based group exercise programs have compared a group exercise program with a nonexercise control,^7^ whereas ours was evaluated against a more challenging usual-care exercise program. Not only was the comparator an active exercise program, but it was also one well taught by trained exercise professionals. Despite being held to a more challenging standard, we were able to demonstrate a greater improvement in mobility with OTM. Second, we were able to demonstrate the effectiveness on site in 3 different settings. All testing and interventions were delivered at the facilities, thus indicating that the program can be conducted in various community locations, which supports implementation in a variety of settings. Third, participants were older, many had multiple chronic conditions and impaired mobility, and approximately one-third reported fear of falling and a history of falls. The participants constituted a somewhat frail group of old-older adults, usually not included in community-based exercise studies.

### Limitations

Some limitations should be considered. In some sites, all individuals were not taught simultaneously, but in classes conducted in series. The study was so designed that during the first session taught by an exercise leader, we could train the staff activity personnel to lead the second session. Consequently, participants randomized to the staff activity personnel class had to wait 12 weeks before starting their exercise sessions. The additional waiting time could have exposed the participants to additional health events, fatigue of waiting, and disappointment of not being taught by a professional. Second, outcomes were examined immediately following the intervention; thus, the long-term intervention effects on mobility, function, and disability and whether the mobility improvements persist over time is unknown. Last, our heterogeneity of treatment examination was exploratory, and we have interpreted cautiously contingent on significant interactions. Interaction tests^43^ have low statistical power and require large samples, and thus we may have been unable to generate hypotheses about important subgroups. With the present findings, a larger study aiming for confirmatory subgroup conclusions may now be warranted.

## Conclusions

From a community-based health promotion and wellness exercise programming perspective, the On the Move group exercise program was more effective at improving mobility than a usual-care group exercise program, despite lower attendance. Additional research examining the impact of the intervention on long-term disability outcomes is needed before routine implementation into clinical practice can be recommended.

## Notes

Supplement 1. Trial Protocol

Supplement 2. **eMethods.** Detailed description of the On the Move and Usual Care interventions **eFigure 1a.** Detailed CONSORT diagram part 1 **eFigure 1b.** Detailed CONSORT diagram part 2
