# Supplementary material for: Effectiveness of a Timing and Coordination Group Exercise Program to Improve Mobility in Community-Dwelling Older Adults: A Randomized Clinical Trial
Source: JAMA Intern Med. 2017 Oct 2;177(10):1437–44. doi: 10.1001/jamainternmed.2017.3609 (PMC5710210; doi:10.1001/jamainternmed.2017.3609)
Supplement: Supplement 1. — Trial Protocol [file jamainternmed-177-1437-s001.pdf]

ON THE MOVE: OPTIMIZING PARTICIPATION IN GROUP EXERCISE TO PREVENT WALKING DIFFICULTY IN AT-RISK OLDER ADULTS

Principal Investigator:

Jennifer S. Brach, PhD, PT

Supported by:

PCORI

CE-1304-6301

Version 1
October 6, 2015

TABLE OF CONTENTS

Page

PRÉCIS **5**

STUDY TEAM ROSTER **6**

1 Study objectives **7**

1.1 Primary Objective 7

1.2 Secondary Objectives 7

2 BACKGROUND AND RATIONALE **7**

3 STUDY DESIGN **10**

4 SELECTION AND ENROLLMENT OF PARTICIPANTS **13**

4.1 Inclusion Criteria 13

4.2 Exclusion Criteria 13

4.3 Study Enrollment Procedures 14

5 STUDY INTERVENTIONS **15**

5.1 Overview 15

5.2 On the Move 15

5.3 Standard 16

5.4 Monitoring of vital signs 16

5.5 Program monitoring 16

5.6 Adherence Assessment 16

6 STUDY PROCEDURES **16**

6.1 Schedule of Evaluations 17

6.2 Description of Evaluations 18

6.2.1 Baseline Assessments 18

6.2.2 Baseline 2 Assessments 20

6.2.3 Post-Intervention Assessment 21

7 SAFETY ASSESSMENTS **21**

7.1 Expected Adverse Experiences 21

7.2 Minimizing Risk during Assessments and Interventions 22

7.3 Confidentiality. 22
7.4 Participant Education about Potential Risks 23
7.5 Adverse Events and Serious Adverse Events 23
7.5.1 Classifying Adverse Events (AE) 24
7.5.2 Severity 24
7.5.3 Expectedness 24
7.5.4 Relatedness 25
7.6 Expected AEs 25
7.7 Reportable AEs (RAEs) 25
7.8 Reporting of Events 26
8 INTERVENTION DISCONTINUATION 26
8.1 Interruption to Exercise Participation 26
8.2 Intervention Discontinuation 26
8.3 Voluntary Participation 27
9 STATISTICAL CONSIDERATIONS 27
9.1 General Design Issues 27
9.2 Sample Size 28
9.3 Data Analyses 30
9.3.1 Overview 30
9.3.2 Main Analysis for Aims 1-3 30
9.3.3 Aim 4 exploratory Analysis 31
9.3.4 Additional Exploratory/Sensitivity and Compliance/Dropout Analyses 32
10 DATA COLLECTION AND QUALITY ASSURANCE 32
10.1 Data Collection Forms 32
10.2 Data Management 33
10.3 Quality Assurance 33
10.3.1 Intervention 33
10.3.2 Protocol Deviation Tracking 33
10.3.3 Subject Termination 34
11 PARTICIPANT RIGHTS AND CONFIDENTIALITY 34
11.1 Institutional Review Board (IRB) Review 34
11.2 Informed Consent Forms 34
11.3 Participant Confidentiality 34

11.4 Study Discontinuation ... 35

12 STUDY TIMELINE ... 35

13 PUBLICATION OF RESEARCH FINDINGS ... 36

14 REFERENCES ... 37

PRÉCIS

Community-dwelling older adults fear loss of independence and nursing home placement more than death. Walking difficulty often leads to loss of independence. Exercise is beneficial to physical and mental health and may prevent walking difficulty and promote independence. Recognizing the importance of exercise, senior housing facilities offer exercise programs to their residents. The exercise programs are often group-based, seated range of motion exercises that do not challenge the older adult; consequently participation rates and resident satisfaction are low. If the goal is to improve walking to promote independence than the exercise program should specifically target walking. Therefore, we developed a challenging, group exercise program entitled "On the Move" which focuses on the fundamentals of walking. In this research study we will determine if the On the Move program is better than a standard program at improving walking and promoting independence and if the same benefits can be obtained if the On the Move program is delivered by staff of the senior living facilities instead of an exercise leader. To answer these questions, 560 community-dwelling older adults living in 28 different Independent Living Facilities and Senior High Rises will be randomly assigned to either the 12 week On the Move group exercise program or the standard group exercise program delivered by either an exercise leader or staff activity personnel. Participants' walking and reported ability to carry out everyday activities (functional ability) will be assessed before and after the 12 week program. We will also assess participant safety and satisfaction with the exercise program and instructor.

The findings from this research study will provide evidence for the value of the On the Move group exercise program and will better inform patient choices regarding participation in exercise programs. If successful in improving walking and promoting independence and acceptable to the older adult, the On the Move program could be incorporated into exercise programming for older adults in community centers, health clubs, and senior residences across the country.

STUDY TEAM ROSTER

Principal Investigator:

Jennifer S. Brach, PhD, PT
Bridgeside Point 1
100 Technology Drive
Pittsburgh, PA 15219-3130
Phone: 412-383-6533
Fax: 412-648-5970
jbrach@pitt.edu

Main responsibilities/Key roles: Oversees and is responsible for all aspects of the study

Co-Investigators:

**Deborah Brodine, MHA, MBA**<br>UPMC Community Provider Services<br>Forbes Tower Suite 10055<br>200 Lothrop St<br>Pittsburgh, PA 15213<br>Phone: (412) 647-0548<br>brodineds@umpmc.edu<br>Main responsibilities: Provider Stakeholder	**Sandra Gilmore, RN, MS**<br>UPMC Community Provider Services<br>101 Orchard Drive<br>Suite 104<br>Trafford, PA 15085<br>Phone: (412)380-8750<br>gilmoresl@upmc.edu<br>Main responsibilities: Provider Stakeholder, community liaison. Identify community sites.
**Neelesh Nadkarni, MD, PhD**<br>Division of Geriatric Medicine<br>Kaufmann Building, Suite 500<br>Pittsburgh, PA<br>Phone: (412) 692-2383<br>nkn3@pitt.edu<br>Main responsibilities: Study physician	**Subashan Perera, PhD**<br>Division of Geriatric Medicine<br>Kaufmann Building, Suite 500<br>Pittsburgh, PA<br>Phone: (412) 692-2365<br>Ksp9@pitt.edu<br>Main responsibilities: Randomization, data management and study statistician
**Jessie VanSwearingen, PhD, PT**<br>Bridgeside Point 1<br>100 Technology Drive<br>Pittsburgh, PA 15219-3130<br>Phone: 412-383-6533<br>jessievs@pitt.edu<br>Main responsibilities/Key roles: Quality control of the intervention	**Edmund Ricci, PhD**<br>207A Parran Hall<br>Graduate School of Public Health<br>University of Pittsburgh<br>130 DeSoto Street<br>Pittsburgh, PA 15261<br>Phone: 412-624-6393<br>emricci@pitt.edu<br>Main Responsibilities: Program evaluation

1 STUDY OBJECTIVES

1.1 Primary Objective

Compare the effects of the *On the Move* group exercise program to a standard program on self-reported function and disability and walking ability. *The On the Move program will produce greater gains in self-reported function and disability (Late Life Function and Disability Index/LLFDI) and walking ability (6-minute walk test/6MWT and gait speed) when delivered by an exercise leader.*

1.2 Secondary Objectives

When delivered by staff activity personnel, assess the effectiveness of On the Move compared to a standard program; and sustainability compared to delivery by exercise leaders. *On the Move delivered by staff activity personnel will produce gains in above outcomes that are greater than the standard program; and comparable to when delivered by an exercise leader.*

Compare the acceptability and the risks of the On the Move and standard exercise programs delivered by exercise leaders and staff activity personnel. *On the Move will result in greater satisfaction and higher attendance rates than the standard program. Attendance rates and satisfaction will be similar for exercise leader and staff activity personnel led programs. Adverse event (falls, soft tissue injuries, muscle soreness, etc.) rates during exercise will be similar between the two groups and the two facilitators.*

Explore potential baseline predictors of benefit and risks of participation in *On the Move* program to facilitate informed patient decision making. *We will be able to identify combinations of baseline physical, psychosocial and demographic factors associated with each of the treatment response and adverse events outcomes.*

2 BACKGROUND AND RATIONALE

Disability is a common, costly problem in older adults. Walking difficulty in older adults contributes to loss of independence, higher rates of morbidity and increased mortality.(1),(2-5) Mobility loss is also a sentinel predictor of other disabilities that restrict independent living.(6) Compared to older adults without self-reported walking difficulty, those who developed mild walking difficulty over one year had higher healthcare costs (mean $1,128 per person). Extrapolated to the estimated 22% of older adults who develop walking difficulty annually, the cost to society is an additional 3.6 billion dollars per year.(7) Therefore, preventing or delaying the onset of walking difficulty might have a substantial impact on older adults' independence and their healthcare costs.

Exercise intervention studies have neglected to include disability outcomes. Difficulty walking is associated with reduced activity and participation and a loss of independence.(8),(9, 10) Exercise

interventions for older adults have focused on improving walking as a means to reduce or delay physical disability.(11, 12) These exercise interventions have included strength, balance and endurance activities in order to reduce impairments and improve physiologic capacity for walking. These studies have resulted in only modest gains in walking ability (i.e. an approximate 5% increase in speed) with only one study reporting disability outcomes.(11, 13-21) Definitive evidence that exercise that improves walking also reduces disability is lacking; therefore the need for the ongoing Lifestyle Interventions and Independence for Elders (LIFE) study.

Exercise interventions fail to include an important component of exercise to improve walking, the timing and coordination of movement. National recommendations and interventions to prevent walking difficulty, such as the Lifestyle Interventions and Independence for Elders (LIFE) study have overlooked an important component of exercise that is critical for walking, the timing and coordination of movement.(20, 22) The ongoing Lifestyle Interventions and Independence for Elders (LIFE) study examines a standard walking endurance, strength, static balance and flexibility intervention on the prevention of disability in community-dwelling older adults. The LIFE pilot study, using the same intervention, demonstrated significant but only modest effects.(20) We have preliminary data to suggest that a novel exercise program that includes timing and coordination exercise is superior to a standard strength and endurance program for improving walking in older adults.(23)

Timing and coordination training improve walking in older adults. We conducted two pilot studies, involving contrasting subject groups, to examine the impact of a timing and coordination exercise program on walking. The first study (RESTORE) included older adults with slow (gait speed < 1.0 m/s) and variable gait and has been published.(23) The second study (PRIME) has just recently been completed. It included older adults with near normal gait speed (gait speed > 1.0 m/s) but with difficulty with aspects of the timing and coordination of walking (i.e. Figure of 8 test time > 8.0 s- see choice of outcomes section below for details of this measure). Participant retention for the 12 week post-tests was over 95%.

In the RESTORE study, 50 subjects (mean age 77.2±5.5 years, 65% women) were randomly assigned to either a standard exercise program (endurance, strength and typical static balance training) or a timing and coordination program, for one hour, 2 times per week for 12 weeks, with baseline and 12 week follow up assessments. Of the 50 who entered, 47 (94%) completed the study. Both groups increased gait speed (timing and coordination by 0.21 m/s and standard by 0.14 m/s). The timing and coordination group reduced the energy cost of walking 0.10±0.03mL/kg/m more than the standard group (p=0.0002), had a 9.8±3.5 point greater gain in reported walking confidence (i.e. Gait Efficacy Scale) than the standard group (p=0.008), had a 1.5±0.6 point greater reduction in gait abnormalities (i.e. GARSM) than the standard group (p=0.02), and had a 3.5±1.7 (p=0.04) point greater gain than the standard group in basic lower extremity function (LLFDI) and a 2.6±1.7 (p=0.12) point greater gain than the standard group in advanced LE function (LLFDI).(23)

In the PRIME study, 38 subjects (mean age 78.5±5.6 years, 65% women) were randomly assigned to either a standard endurance and strength exercise program or a timing and coordination plus strengthening program, 2 times per week for 12 weeks, with assessments at baseline and immediately following the 12 week intervention. Preliminary analyses indicate that

the timing and coordination group had greater improvements in gait speed, figure of 8 walk, and challenging gait tasks than the standard group. Both groups had improvements in the 6MWT ($p<0.05$); however, the timing and coordination group had marginally greater improvements ($p=0.14$). Only the timing and coordination group demonstrated improvement in self-reported disability and neither group decreased perceived walking difficulty (LLFDI – total function). However, this group had very high initial LLFDI function scores; reflecting low levels of baseline perceived walking difficulty, so may have been vulnerable to ceiling effects. Since our proposed sample will be more impaired, we anticipate that there is more room for change and that treatment group differences or declines of perceived walking difficulty will be detectable.

Our preliminary data demonstrate the benefit of timing and coordination training and support the concept that timing and coordination training provides something distinct from standard training, with potential effects on self-reported function, disability and mobility. Note that these pilot studies examined the effects of the timing coordination program delivered on a one on one basis. In the current proposal we will be testing the timing and coordination training delivered as a group program (i.e. On the Move group exercise program).

Exercise programs offered in senior housing are inadequate. Recognizing the importance of exercise for promoting physical and mental health many senior housing facilities offer exercise programs for the older adults. Though available, participation rates and participant satisfaction are low. Older adults feel the seated group exercise programs that are offered, are not challenging or beneficial so they stop participation. Providers, such as UPMC Senior Communities, are looking for viable alternatives. Older adults are interested in exercise interventions that will improve their mobility and help maintain their independence (focus group information). Exercise programs currently offered in senior living settings are inadequate because they 1) are often conducted in seated position and do not challenge the older adult and 2) exclude an important component of exercise that is critical to walking, the timing and coordination of movement. There is a need for a more challenging, evidence-based exercise program that is designed to improve walking and promote independence in older adults.

We developed a challenging, evidence-based group exercise program, On the Move, to improve walking in older adults. Though effective, an individualized, physical therapist led exercise program (described above as timing and coordination training) is not a cost effective model for the prevention of walking difficulty. A group exercise program is a cost efficient alternative which would likely promote adherence through socialization. Therefore, in collaboration with UPMC Community Provider Services, we developed a novel timing and coordination group-based exercise program entitled “On the Move”. The On the Move program differs from current group exercise programs in that 1) it contains timing and coordination exercises based on the biomechanics and motor control of walking (i.e. specificity of training), 2) the majority of the program consists of standing and walking exercises which challenge the older adult, and 3) it was developed with input from older adults.

UPMC Community Provider Services is interested in the sustainability of the exercise program. A main priority of our stakeholder, UPMC Community Provider Services, is the sustainability of the exercise program. Often scientists conduct research within their facilities and when the research is over, the scientists move on to another project and the program does not continue.

Recognizing this as an issue, we incorporated a sustainability component into our Aging Institute pilot described above. During the 12 week exercise program, the exercise leader is training the activity staff personnel to conduct the program. The activity staff personnel is currently assisting with the 12 week program. After 6 weeks of exercise classes we surveyed the activity staff personnel to determine her level of confidence in conducting the program. The activity staff responded that she was very confident that she could identify participants who were doing the exercises correctly and/or incorrectly and she was confident that she could lead the exercise program and progress the exercises. The activity staff personnel stated that a "cheat sheet" of listed exercises would be very useful when leading the exercise class. At the conclusion of the first 12 week exercise leader program, the staff activity personnel will start a new 12 week program with residents who were placed on the wait list.

Summary

The evidence-based On the Move program was developed based on our past research and with input from older adults. In our initial pilot study, we demonstrate that the program is feasible and acceptable to the older adult and that activity staff personnel have confidence in their ability to lead the program. In our current pilot we demonstrate our ability to engage management from other community sites and the ability to recruit from different populations. The next key step is to examine the benefits and risks of the On the Move program in a larger, more diverse sample of older adults and to assess the sustainability of the program. Specifically, we are interested in determining if the challenging group-based program improves mobility and prevents functional decline and disability without any increased risk to the older adult and if the activity staff personnel can lead the On the Move exercise program obtaining similar results to the exercise leader delivered program.

3 STUDY DESIGN

To address our research questions, we will conduct a cluster randomized single-blind two arm intervention trial to compare the effects on function, disability and mobility of a standard group exercise program and a novel *On the Move* group exercise program in 560 community-dwelling older adults who reside in 8 independent living facilities and 20 senior housing sites. Group exercise classes are twice weekly for 12 weeks and will be delivered by exercise leaders and activity staff personnel. Function, disability and mobility are assessed at baseline and post intervention **(Figure 1).**

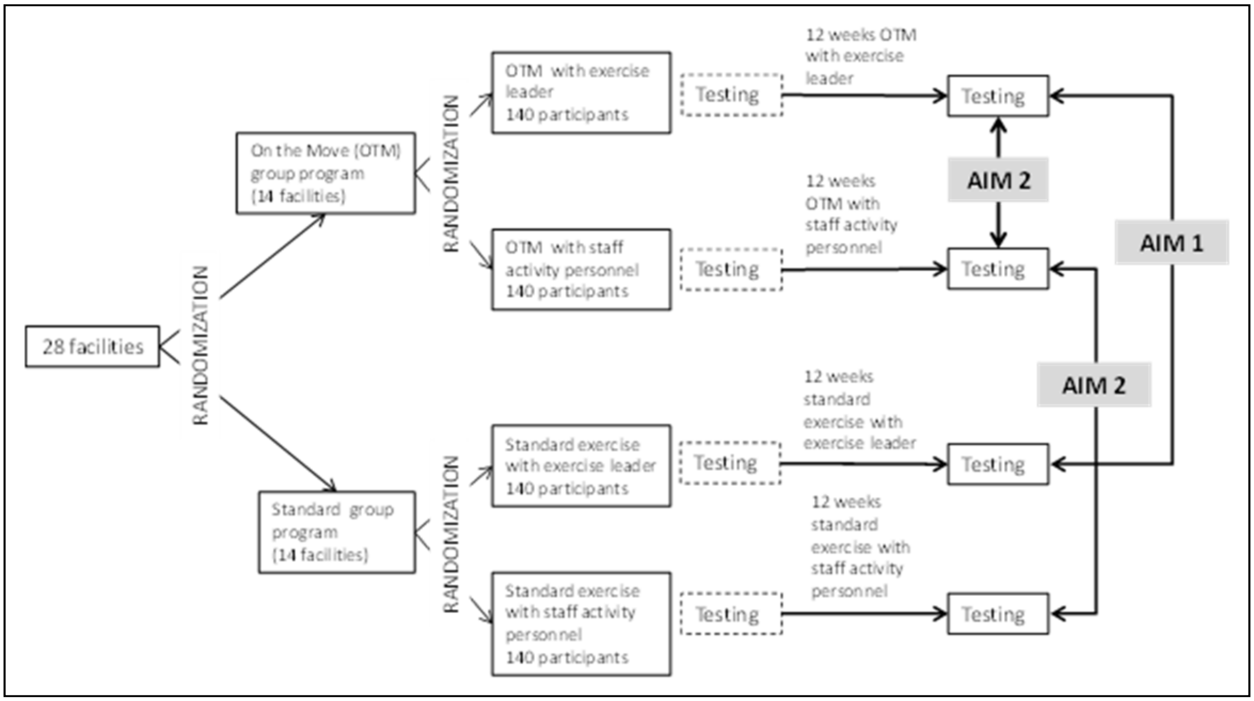

<u>Choice of comparators</u>

There are two main comparisons being examined, 1) the type of exercise program (i.e. *On the Move* versus a standard group exercise program) and 2) the delivery mode (i.e. exercise leaders versus staff activity personnel).

Exercise program comparison (Aim 1). The novel challenging *On the Move* exercise program will be compared to a standard group exercise program. Both exercise programs (*On the Move* and standard) will be group-based and led by a person. Our older adult participants have stressed the importance of having the exercise program led by a person instead of viewing a videotape. They feel the person is more enjoyable and they like the idea of the instructor providing feedback about their performance throughout the exercise class. Both programs will be delivered by trained exercise leaders or trained staff activity personnel (sustainability component). The frequency and duration of the programs are identical (50 minutes, 2 times a week for 12 weeks). From our past research we have determined that a frequency of 2 times per week for 12 weeks is acceptable to the older adult participants and is an adequate dose to obtain meaningful outcomes.(23) The main difference between the standard and the *On the Move* group exercise programs is the program content which is described below.

The <u>*On the Move* exercise program</u> is based on principles of motor learning that enhance "skill" or smooth and automatic movement control.(24-29) The program contains a warm-up (5 minutes), stepping patterns (15 minutes), walking patterns (15 minutes), strengthening exercises (10 minutes), and cool-down exercises (5 minutes). The warm-up and cool down contain gentle range of motion exercises and stretches for the lower extremities and trunk. The stepping and walking patterns are goal-oriented progressively more difficult patterns which promote the

timing and coordination of stepping, integrated with the phases of the gait cycle.(25, 26, 28, 29) Conceptually, the exercise is intended to achieve its effects by shifting the center of pressure posteriolateral then forward, encouraging hip extension prior to stepping, loading the trailing limb, coordinating activation of the abductors of the soon to be swing leg with adductors of the stance limb, and shifting the center of pressure in medial stance to unload the stepping limb.(30-32) Progression is based on first separately increasing the speed, amplitude or accuracy of performance prior to undertaking a more complex task.(33) For example, the progression of stepping patterns is, 1) self-paced step forward and across, 2) increase stepping speed, 3) alternate side of stepping, 4) alternate forward with backward stepping. In the group class, exercises can be individualized by having some older adults use upper extremity support while others do not hold on during the exercises. Also, some subjects will step in all one direction while others will do the more challenging alternating left and right steps. Walking patterns incorporate patterns of muscle coordination and interlimb timing into walking. Walking patterns progress by altering speed, amplitude (e.g. narrowing oval width), or accuracy of performance (e.g. without straying from the desired path), and then to complex walking patterns involving walking past others and with upper extremity object manipulation tasks, such as carrying or bouncing a ball.(29) Walking and stepping patterns (i.e. timing and coordination training) were used in both of our pilot studies.(23) The strengthening exercises are conducted in sitting and standing and target the lower extremity muscles. The majority of the program will be conducted in standing (40 minutes) with only a small portion conducted in sitting (10 minutes). We have conducted two pilot exercise classes with older adults with varying levels of ability. We were able to successfully individualize the group program in that participants reported feeling both challenged and safe. Please see the appendix for example exercises and progressions.

The standard group exercise program is based on exercise programs that are currently being conducted at the facilities (i.e. standard of care). The operationally defined program contains a warm-up (5 minutes), upper and lower extremity strength and flexibility exercises (30 minutes), static balance exercises (10 minutes) and a cool-down (5 minutes). The majority of the program will be conducted in sitting (40 minutes) with only a small portion (10 minutes) conducted in standing. The active control group (i.e. standard exercise program) will aid in adherence and retention in that subjects who volunteer to participate in the study will be looking for exercise options.

Sustainability of the program: exercise leader and staff activity personnel comparison (Aim 2). The sustainability of the program will be evaluated by assessing the effectiveness of *On the Move* compared to a standard program when delivered by staff activity personnel and by comparing outcomes obtained by the *On the Move* program delivered by exercise leaders and staff activity personnel **(Figure 1)**. Exercise leaders are individuals with training and experience in administering exercise programs, such as exercise physiologists, physical therapists, physical therapy assistants, etc. Staff activity personnel are employees of the Independent Living Faciltities and Senior High Rises that are involved in providing services to the residents. They could be activity directors, social workers, outreach coordinators, care coordinators, etc. These individuals are not specifically trained to deliver exercise interventions as part of their job. We will work with Holly Rolt, Missy Sovak and Sandra Gilmore to identify staff activity personnel at each of the sites who would be involved in this research study. All exercise leaders and identified staff activity personnel will be trained in the delivery of the exercise program prior to

leading any exercises classes. The staff activity personnel will participate in a one hour training sessions in which the rationale and the general format of the program will be explained. During these training sessions the activity staff personnel will participate in a sample exercise class. In addition to the 2 training sessions, the activity staff personnel will be able to observe the 12 week exercise session delivered by the exercise leader at their facility. They will be given the opportunity to observe all 24 sessions if they would like. In addition to training the exercise leaders and activity staff personnel, we will provide printed materials explaining the exercise program that we developed in our pilot work (see appendix). A study investigator (Dr. Brach or Dr. VanSwearingen) will meet with the staff activity personnel or email them periodically (i.e. during weeks 1, 3, 6, and 10 of the 12 week exercise program) to observe the intervention, monitor treatment fidelity, and to answer any questions.

4 SELECTION AND ENROLLMENT OF PARTICIPANTS

We will recruit participants from the UPMC Independent Living Facilities (ILFs) and the Senior Housing sites. There are eight different ILFs with over 700 residents. UPMC is affiliated with 32 different Senior Housing sites that house over 2,000 residents. We anticipate enrolling subjects from all 8 ILFs and from 20 of the Senior Housing sites for a total of 28 sites. We plan to enroll 20 subjects from each site or 160 (20%) from the ILFs and 400 (21%) from the Senior Housing Sites.

4.1 Inclusion Criteria

Participants must meet all of the following inclusion criteria to participate in the study.

1) 65 years of age or older
2) Resident of a UPMC ILF or Senior Housing site
3) Ambulate independently for household distances with or with a straight cane,
4) Usual gait speed greater than or equal to 0.60 m/s

4.2 Exclusion Criteria

Potential participants who meet any of the following exclusion criteria at baseline will be excluded from study participation.

1) Non English speaking,
2) Impaired cognition, defined as inability to follow 2 step commands or understand the informed consent process,
3) Plans to leave the area for an extended period of time over the next 4 months,
4) Progressive neuromuscular disorder such as Parkinson's or Multiple Sclerosis,
5) Any acute illness or medical condition that is not stable,
6) Inappropriate response to the 6 minute walk test (i.e. exercise heart rate >= 120 bpm, exercise SBP >= 220 or a drop in SBP > 10 mmHg, or DBP >= 110 mm Hg).

4.3 Study Enrollment Procedures

We will hold information sessions at each of the sites. The study PI (Dr. Brach) will visit each site and describe the study to the residents. Subjects who are interested in hearing more will be asked to place their name and phone number on a sign-up sheet. Research staff will then contact the subject to explain the study and conduct the initial phone screen if the subject is interested.

Subject eligibility will be determined from a phone screen and an in-person clinical screen. Researchers will contact potential participants by phone to determine their eligibility. After obtaining verbal consent, a structured screening questionnaire (see attached) will be administered to determine the presence and absence of the inclusion/exclusion criteria. Individuals who meet the criteria will be scheduled for an in-person screening visit that will take place at the ILF or senior housing site.

At the in-person screening visit, the first task will be to obtain informed consent for participation in the study. Once informed consent is obtained, the screening exam will take place. The screening exam will be conducted by trained research personnel (physical therapists, exercise physiologists, physical therapy assistants, or physical therapy students) who have experience working with older adults and conducting such screening measures. ACSM Guidelines regarding exercise participation will be available to all staff during testing. At all screening sessions at least 1 physical therapist will be available as a resource for the other research staff conducting screening procedures.

The purpose of the physical examination is to identify potential exclusion issues that are not identified by self-report. The exam includes a review of systems, current medications, vital signs, lower extremity range of motion and strength testing, and visual screening. A standard demographics questionnaire will be used to determine age, gender, race, marital status and work history in order to adequately describe the research participants. As a screening for exercise participation, all subjects will complete a six minute walk test (additional detail under experimental procedures). Participants will be asked to walk as far as they can in six minutes (without jogging or running) in a hallway. Participants are permitted to stop and rest during this test, if needed, and the number and duration of rest breaks are recorded. Blood pressure, heart rate and level of fatigue (using the rate of perceived exertion (RPE) scale) are recorded before and after the test. Based on the ACSM guidelines, subjects who have an inappropriate exercise response (i.e. exercise heart rate >= 120 bpm, exercise SBP >= 220 or a drop in SBP > 10 mmHg, or DBP >= 110 mm Hg) will be referred to their primary care physician for clearance before they can be enrolled in the study.(34) Subjects who have an appropriate exercise response and are eligible based on the other screening criteria will then complete the outcome measures of function, disability and walking ability described below. All subjects will sign a “Liability Waiver Release for Participation in an Exercise Program” prior to starting the exercise program.

5 STUDY INTERVENTIONS

5.1 Overview

The exercise interventions will be delivered in 2 phases. The first phase or 12 week class will be delivered by the exercise leader. The second phase or 12 week class will begin once the first phase is completed and will be delivered by staff activity personnel. Participants who are randomized to the second phase class (i.e. delivered by staff activity personnel) will repeat the baseline testing prior starting the exercise class.

The facilities will be randomized (described above) to receive either the "On the Move" or a standard group exercise program. Both exercise programs (On the Move and standard) will be group-based and led by a person. Both programs will be delivered by trained exercise leaders or trained staff activity personnel (sustainability component). The frequency and duration of the programs are identical (50 minutes, 2 times a week for 12 weeks). From our past research we have determined that a frequency of 2 times per week for 12 weeks is acceptable to the older adult participants and is an adequate dose to obtain meaningful outcomes.(23) The main difference between the standard and the On the Move group exercise programs is the program content which is described below.

5.2 On the Move

The On the Move exercise program is based on principles of motor learning that enhance “skill” or smooth and automatic movement control.(24-29) The program contains a warm-up (5 minutes), stepping patterns (15 minutes), walking patterns (15 minutes), strengthening exercises (10 minutes), and cool-down exercises (5 minutes). The warm-up and cool down contain gentle range of motion exercises and stretches for the lower extremities and trunk. The stepping and walking patterns are goal-oriented progressively more difficult patterns which promote the timing and coordination of stepping, integrated with the phases of the gait cycle.(25, 26, 28, 29) Conceptually, the exercise is intended to achieve its effects by shifting the center of pressure posteriolateral then forward, encouraging hip extension prior to stepping, loading the trailing limb, coordinating activation of the abductors of the soon to be swing leg with adductors of the stance limb, and shifting the center of pressure in medial stance to unload the stepping limb.(30-32) Progression is based on first separately increasing the speed, amplitude or accuracy of performance prior to undertaking a more complex task.(33) For example, the progression of stepping patterns is, 1) self-paced step forward and across, 2) increase stepping speed, 3) alternate side of stepping, 4) alternate forward with backward stepping. In the group class, exercises can be individualized by having some older adults use upper extremity support while others do not hold on during the exercises. Also, some subjects will step in all one direction while others will do the more challenging alternating left and right steps. Walking patterns incorporate patterns of muscle coordination and interlimb timing into walking. Walking patterns progress by altering speed, amplitude (e.g. narrowing oval width), or accuracy of performance (e.g. without straying from the desired path), and then to complex walking patterns involving walking past others and with upper extremity object manipulation tasks, such as carrying or bouncing a ball.33 Walking and stepping patterns (i.e. timing and coordination training) were used in both of our pilot studies.5 the strengthening exercises are conducted in sitting and

standing and target the lower extremity muscles. The majority of the program will be conducted in standing (40 minutes) with only a small portion conducted in sitting (10 minutes). We have conducted two pilot exercise classes with older adults with varying levels of ability. We were able to successfully individualize the group program in that participants reported feeling both challenged and safe. Please see the appendix for example exercises and progressions.

5.3 Standard

The standard group exercise program is based on exercise programs that are currently being conducted at the facilities (i.e. standard of care). The operationally defined program contains a warm-up (5 minutes), upper and lower extremity strength and flexibility exercises (20 minutes), cardiovascular exercises (20 minutes) and a cool-down (5 minutes). The majority of the program will be conducted in sitting (>40 minutes) with only a small portion (<10 minutes) conducted in standing. The active control group (i.e. standard exercise program) will aid in adherence and retention in that subjects who volunteer to participate in the study will be looking for exercise options.

5.4 Monitoring of vital signs

Vital signs will be monitored before and after the exercise class as needed. Vitals signs will be monitored more at the beginning of the program, and at any time the participant reports or displays signs and symptoms (i.e. shortness of breath, lightheadedness, racing heart, etc). We will once again follow the ACSM guidelines for stopping of exercise.

5.5 Program monitoring

Periodically (approximately 1-2 times) throughout the exercise program we will be videotaping the exercise sessions. These videos will be used primarily to monitor the quality and consistency of the exercise program and to further develop the training manual for the exercise instructors. The videos may be used to train future exercise instructors.

5.6 Adherence Assessment

A roster of participants will be developed for each group exercise class. At the beginning of each class, attendance will be recorded by the exercise leader or staff activity personnel. Reasons for missed classes will be recorded when available. Attendance rate ([number of sessions attended by the participant/total number of classes offered, i.e 24] X100%) will be calculated for each participant and will be the main indicator of adherence.

6 STUDY PROCEDURES

6.1 Schedule of Evaluations

Measure	Clinic Screen	Baseline 1<br><br>Pre-intervention	Baseline 2<br><br>Pre-intervention Activity staff group	12 week<br><br>Post-intervention
Demographics Questionnaire	X			
Physical exam screen (BP, strength, 2 step command)	X			
6 MWT	X		X	X
Screening gait speed	X			
Comorbidities Index		X		
Fall history		X	X	X
Anthropometric Measurements		X		
Gait Measures				
Gait speed – Zeno Walkway		X	X	X
Complex walk (Shumway-Cook)		X	X	X
SPPB		X	X	X
Figure 8		X	X	X
GES		X	X	X
Physical Function				
LLFDI		X	X	X
Global items		X	X	X
Potential confounders				
PHQ-9		X	X	X
Digit Symbol Substitution Test		X	X	X
Program Evaluation				
Satisfaction survey				X

6.2 **Description of Evaluations**

6.2.1 Baseline Assessments

If after the clinic screen the participant is eligible (i.e. meets all inclusion/exclusion criteria) they will undergo baseline testing. All baseline testing will be performed by research staff trained in the testing procedures. All testing will be completed at the ILFs and Senior Housing Sites.

Our main outcomes, function, disability, and walking ability are highly associated with independence and are extremely important to the older adult. Our primary measure of function and disability is the Late Life Function and Disability Instrument (LLFDI) and our main measures of walking ability are Six Minute Walk Test (6MWT) and gait speed. We will also examine confidence in walking (Gait Efficacy Scale), walking under challenging conditions (challenging gait tasks and figure of 8 walk), gait variability and the Short Physical Performance Battery (SPPB) as additional measures of walking ability. We will also collect a measure of cognition (Digit Symbol Substitution Test) and mood (PHQ-9).

6.2.1.1 Function and Disability

Late Life Function and Disability Instrument (LLFDI).(35, 36) Our primary function and disability outcome will be the LLFDI. The LLFDI is a pair of self-report measures targeted for assessing physical function and disability in older adults with acute or chronic problems, and designed to be more sensitive to change than similar measures. The two components of the LLFDI correspond to the activity (LLFDI – function) and participation (LLFDI – disability) components of the World health Organization's International Classification of Function, Disability and Health model. The LLFDI function component has 32 items in three dimensions, basic lower extremity (BLE), advance lower extremity (ALE) and upper extremity (UE) and the LLFDI disability component has 16 items representing two dimensions, frequency of performance and limitation in performance of life tasks. We've selected the LLFDI because 1) it measures both function and disability which are critical components of independence, 2) it includes a wide variety of life tasks in various social areas thus extending beyond the traditional focus of just activities of daily living, 3) the scale was designed with sufficient breadth of items and increments of rating in order to minimize ceiling and floor effects and maximize the scale's ability to detect change over time, and 4) it is a continuous outcome which gives us greater power than a dichotomous outcome to detect change over time. We will focus our analyses on the LLFDI function and disability dimension scores (i.e. BLE function, ALE function, UE function, disability frequency and disability limitation). We will also examine the disability domain scores (social role, personal role, instrumental role and management role) since they may provide insight into the impact of the disability on frequency of performance and perceived limitations.(36) The LLFDI function and disability scales have established known groups validity and the test-retest reliability is moderate to high for the disability component (ICCs range from 0.68 to 0.82) and extremely high for the function component (ICCs range from 0.91-0.98 for the dimensions). Scores range from 0-100; higher scores represent less difficulty and less disability.

6.2.1.2 Walking Ability

Six-Minute Walk Test (6MWT). One of the main walking ability outcomes is the Six-Minute Walk Test (6MWT) of distance walked (meters) in six minutes, including time for rest as needed.(37) We have selected the 6MWT because it is 1) a performance-based measure of walking ability and walking ability is an important component of independence, 2) an indicator of community ambulation (i.e. the ability to walk 300m in 6 minutes),(38, 39) 3) a continuous outcome which gives us greater power than a dichotomous outcome to detect change over time,(40) and 4) a widely used measure of mobility that is included in the NIH PROMIS project to establish measures of clinical assessment. The 6MWT has established psychometric properties, test-retest reliability (Pearson r=.95) in older adults,(41, 42) construct validity for graded exercise test and functional classification.(43) The Six-Minute walk test will be completed as part of the clinic screen (described earlier) and also used as an outcome measure. Participants will only complete the 6MWT once at baseline. Participants will be asked to walk as far as they can in six minutes (without jogging or running) in a hallway. Participants are permitted to stop and rest during this test, if needed, and the number and duration of rest breaks are recorded. Blood pressure, heart rate and level of fatigue (using the rate of perceived exertion (RPE) scale) are recorded before and after the test. Based on the ACSM guidelines, subjects who have an inappropriate exercise response (i.e. exercise heart rate >= 120 bpm, exercise SBP >= 220 or a drop in SBP > 10 mmHg, or DBP >= 110 mm Hg) will be referred to their primary care physician for clearance before they can be enrolled in the study.(34) Greater distance covered during six minutes is better.

Gait Speed. The second main walking ability outcome is gait speed. We have selected gait speed because it is a strong indicator/predictor of morbidity and mortality in the older adult.(2, 3, 5) Gait speed is assessed in usual walking with an instrumented walkway. After explanation, the participant completes 2 practice walks to become accustomed to walking on the walkway. The subject then completes 4 passes at their usual, self-selected walking speed. Gait speed will be averaged over the 4 passes. The test-retest reliability of gait speed measured using instrumented walkways by ICC is 0.98.(44) A faster speed is better.

6.2.1.3 Additional Mobility Measures

Gait Efficacy Scale. In order to determine if changes in walking ability are associated with changes in confidence in walking, confidence will be assessed using the Gait Efficacy Scale.(45-47) The items include a range of gait activities such as walking over different surfaces, up and down curbs, and negotiating stairs. Each item has a 10 point Likert scale scoring option, with the total score for the 10 items, ranging from 0-100. A higher score represents greater confidence.

Figure of 8 Walk.(48) The Figure of 8 Walk was designed to measure motor skill in walking. The test involves walking a figure of eight pattern about two markers placed 5 feet apart. Performance is scored based on the time to complete the figure 8 walk and the number of steps. Faster and fewer steps are better.

Challenging Gait Tasks. Challenging gait tasks are used to examine an individual’s ability to adapt their gait to different environmental conditions.(49)53 Subjects will complete two, 12 meter trials of each challenging condition, obstacle, curved path, and narrow path.(49) The time

to complete each task, averaged over two trials, is the summary indicator of gait during challenging tasks. In a sample of 40 community-dwelling older adults, the 1-week test re-test reliability of the timed measures of challenging gait ranged from ICC = 0.70 to 0.94. The marginal additional time for completing each challenging task compared to usual gait is the main indicator and lower marginal cost is better.

Gait Variability. Gait variability, defined as fluctuations in gait characteristics from one step to the next,(50) is an important indicator of impaired mobility in older adults.(9) Gait variability is quantified using established measures of temporal and spatial gait characteristics including stance time, step length, and step width. Variability will be calculated as the standard deviation of the set of steps recorded over 4 passes on the instrumented walkway (described above). Approximately 32 steps will be collected from 4 passes on the mat which will be more than adequate to achieve a stable measure of gait variability. Our prior work has shown that 20 steps are sufficient to achieve a reliability of 0.75 and 30 are sufficient for 0.80.(51) In general, lower variability is better although there are exceptions.(9, 52)

Short Physical Performance Battery (SPPB). The SPPB of lower extremity function was designed and used in the Epidemiologic Studies of the Elderly (EPESE) to assess lower-extremity function of individuals 65 years of age and older. It combined measures of gait speed, balance, and timed chair rise to develop the SPPB for lower-extremity function. The three components are: gait speed over 4-meters, standing balance and chair stands are timed and these times are converted to scores from 0-4 (0=unable, 4=fastest time) for each component. Total scores on the Battery range from 0-12, ranges defined for relative risks of disability.(4)

6.2.1.4 Cognition and Mood

Digit Symbol Substitution Test.(53)A measure of processing speed, the Digit Symbol Substitution test will be used to gather information about both motor and cognitive processing speed. The DSST is a paper and pencil task from the WAIS III that provides normed measures of both motor and cognitive processing speed. The DSST has been widely used in studies of physical and cognitive performance of older adults.

Patient Health Questionnaire (PHQ-9).(54) The PHQ-9 is the 9 item depression scale of the Patient Health Questionnaire. It asks participants to describe how they have been feeling over the past 2 weeks. It has been used successfully in older adults.

6.2.2 Baseline 2 Assessments

Individuals who are randomized to the second exercise session taught by the staff activity personnel we complete a second baseline assessment prior to initiating the exercise class. This will be approximately 12 weeks after the initial baseline assessment. Baseline 2 Assessment will be identical to Baseline testing.

6.2.3 Post-Intervention Assessment

At the completion of the 12 week exercise program, all participants will undergo a post-intervention assessment. The assessment will include all measures from the baseline assessment (LLFDI, 6MWT, gait speed, Gait Efficacy Scale, walking under challenging conditions (challenging gait tasks and figure of 8 walk), gait variability, SPPB, DSST, PHQ-9. Post-intervention testing will be completed at the sites (ILFs and senior housing sites) by research personnel masked to the intervention group and trained in the testing procedures.

In addition, we will also assess participant satisfaction. Participant satisfaction will be assessed using in-depth interviews and a satisfaction survey. In-depth phone interviews will be used to assess satisfaction in a subsample of participants (approximately 20%). We will randomly select a diverse sample of older adults representing the different intervention arms (On the Move, Standard, exercise leader, activity staff personnel) and the different sites (ILF and senior housing). We selected in-depth interviews instead of focus groups because we are more likely to draw people out, they are less intimidated, and more likely to be truthful. The interview will be developed by The Evaluation Institute under the direction of Dr. Edmund Ricci (Co-Investigator) and with input from the other investigators and stakeholders (Community Advisory Board). The interview will include a mixture of closed and open-ended questions. Sample topics to be included in the interviews include perceived benefits and risks, satisfaction with the program, facilities, and instructor, and amount of individualized instruction. The interview will be pilot tested and modified accordingly prior to administration in the study. Pilot testing will ensure that the questions are understandable to our target population and that we are obtaining the information and feedback that we hope to obtain.

Satisfaction will also be assessed by surveys in all participants at the conclusion of the exercise program. The satisfaction survey includes five items measured on a Likert scale and three yes/no questions. The Likert items will assess degree of satisfaction with various components of the exercise program (i.e. the exercises, instructor, music, space, and the overall program). We will also use a series of yes/no questions to determine if the program has meet the participants' expectations, if they feel they have benefited from the program, and if they would recommend the program to others.

7 SAFETY ASSESSMENTS

7.1 Expected Adverse Experiences

1. Major risks such as a cardiac event or a fall are expected to be rare – expected to occur in less than 1% of people (less than 1 out of 100 people).(55) Gardner et al, 2000,(55) reviewed controlled clinical trials of exercise interventions for older adults at-risk for falling. No cardiac events or falls were reported in the 12 clinical trials reviewed. The at-risk older adults in the studies reviewed have slightly poorer physical performance than the older persons we expect to recruit for our study. In our recent MOBILE study, a 1 year cohort study of 120 older adults participating in 3 clinic visits over a 1 year period there were no cardiac events or falls reported. In all conditions of testing in which the

participant is standing and/or walking (eg conditions with a potential risk for falling), the participant will be directly supervised by the trained tester. The trained tester is present for all testing sessions. We expect this level of supervision reduces the risk of falling to an even greater degree.

2. Less severe risks of participation such as such as muscle soreness, fatigue, or minor sprains or strains with each assessment, are expected to be infrequent – expected to occur in 1-10% of people (1-10 out of 100 people). Gardner et al, 2000,(55) reviewed controlled clinical trials of exercise interventions for older adults at-risk for falling, finding reports of such side effects of the intervention reported in only 4 of the 12 studies reviewed. The side effects were not a reason for dropout from the study and were described as soreness or musculoskeletal symptoms, but no injuries.
3. There is a rare risk that confidentiality could be breached. All of the research records will be kept in a locked file cabinet and/or password protected files. All of the investigators and staff that assist with the management of the files are trained in the privacy and confidentiality regulations that govern research.

7.2 Minimizing Risk during Assessments and Interventions.

All assessments approved in this study are considered to be a part of everyday clinical practice. We have minimized risks we believe by applying usual safeguards for the assessment of gait. Assessment side effects, such as muscle soreness, fatigue, or minor sprains or strains with each assessment, will be recorded by the physical therapist and monitored by Dr. Brach, the Principal Investigators in consultation with the physician investigator, Dr. Nadkarni. Based upon the existing literature and our own clinical experience, we anticipate the frequency of these side effects to be extremely low.(55)

We will maximize the safety of our subjects with the following procedures.

1. Individuals with absolute contraindications to testing will be excluded based on the inclusion/exclusion criteria.
2. Testing will be carefully monitored by trained testers and will be adjusted according to the American College of Sports Medicine Guidelines for Exercise Testing and Prescription.(34)
3. In all conditions of testing in which the participant is standing and/or walking (eg conditions with a potential risk for falling), the participant will be directly supervised by the trained tester.

7.3 Confidentiality.

Participant's confidentiality will be protected in the data collection process. All personnel involved with the research have read and signed a Confidentiality statement, and approval is being obtained from the University of Pittsburgh Biomedical Institutional Review Board. Consent forms and data collection forms that identify the participant by name will be stored in a locked cabinet. All computers are password protected. If the data are used in scholarly presentations or journal articles, the investigators will protect the anonymity of individual

participants and will report only aggregate data (eg group means) where appropriate. The Principal Investigator will review data confidentiality processes monthly or as indicated with the project staff. The Investigators are all certified in Research Practice Fundamentals, Human Subjects Research Module**.**

7.4 **Participant Education about Potential Risks**

Potential risks associated with study-related activities and interventions will be explained to each participant by trained study personnel during the informed consent process. Each participant will be instructed to report the occurrence of an AE to appropriate study staff at scheduled data collection times, to PTs administering the intervention, or spontaneously at any other time. Participants also will be encouraged to report concerns about the safety of participating in the study to any research staff.

7.5 **Adverse Events and Serious Adverse Events**

If an adverse event occurs during testing or intervention, staff or activity personnel will assess the situation and provide immediate assistance to the participant if necessary. If a medical emergency should occur, staff will immediately contact 911. They will describe the incident to the 911 operator and provide their location. They will also contact the facility director to make them aware of the situation. In each testing/exercise space, we will post a copy of the emergency plan which will include the number to call (911) and the address of the facility. All staff will be informed of the emergency procedures prior to starting the interventions and they will be reviewed every 6 months. Once the emergency situation is under control, the staff will contact the study PI to notify them of the situation. The PI will report unexpected or adverse events in accordance with the University of Pittsburgh IRB guidelines. As a group the PI and Co-Is review the adverse events to determine if they are expected vs unexpected and serious vs not serious. All events are reported to the irb who will also review the events. The study coordinator (Ms. Betts), study biostatistician (Dr. Subashan Perera) and study physician (Dr. Neelesh Nadkarni) who are unblinded will track the events to determine if there are an unequal number of events between the groups.

A clinical complication form will be completed any time an incident (accident, injury, illness, problems with medication, etc.) occurs or a subject reports an episode. Complications will be categorized as study related, possibly study related, or not study related. Any complications that are categorized as either study related or possibly study related will be reported to the IRB per protocol if the event is an unexpected event, and/or is of serious or moderate to severe in nature as defined by the IRB. A separate clinical complication form will be completed for each incident that may occur in each subject. The form will be kept in the subject's research record.

7.5.1 Classifying Adverse Events (AE)

An AE is any unfavorable or unintended medical occurrence in a human study participant that has taken place during the course of a research project, including any abnormal sign, symptom, or disease, whether or not related to participation in the research.

For the purposes of this study, any event that meets the criteria for a severe adverse event (SAE), is unexpected, or results in injury to the participant while he/she is under the supervision of study related personnel will be classified as a reportable adverse event (RAE). Adequate review, assessment, and monitoring of RAEs require they be classified as to severity, expectedness, and potential relatedness to the study intervention.

7.5.2 Severity

The following guidelines will be used to determine level of severity:

Mild: Awareness of signs and symptoms, but easily tolerated and causing no loss of time from normal activities. No specific medical attention is required.

Moderate: Discomfort enough to cause a low level of inconvenience or concern to the participant and may interfere with daily activities. Symptoms may require minimal, local or noninvasive medical intervention.

Severe: Events interrupt the participant's normal daily activities and are usually incapacitating. Significant symptoms may require hospitalization or invasive medical intervention.

Life-threatening/Disabling: Events that may involve acute, life-threatening metabolic or cardiovascular complications (such as circulatory failure, hemorrhage, sepsis) or life – threatening physiological consequences. Intensive care or emergent invasive procedure is required.

Death: Causing death.

Severity is not synonymous with seriousness. A severe headache is not necessarily an RAE. However, mild chest pain may results in a day's hospitalization and thus would be classified as a RAE.

7.5.3 Expectedness

AEs will be assigned as to whether they were expected or unexpected based on current knowledge. Categories are defined as follows:

<u>Expected:</u> An AE that is anticipated on the basis of prior experience with the intervention under investigation; an event that can be attributed to the underlying condition of the participant being studied; or an event that can be attributed to the patient population being studied (see section--- Expected AEs). Expected AEs are captured in a standardized way by study personnel.

Unexpected: An AE that was not anticipated on the basis of prior experience with the underlying intervention under investigation; an event that can be attributed to the underlying condition of the participant being studied; or to the patient population being studied or an expected event whose frequency or severity exceeds what is anticipated. Unexpected events are reportable.

7.5.4 Relatedness

The PI in consultation with the Co-Investigators and an independent safety monitor will determine the degree to which RAEs are related to study procedures using the criteria below.

Definitely related: The adverse event is clearly related to the investigational procedure – i.e., an event that follows a reasonable temporal sequence from administration of the study intervention, follows a known or expected pattern of response to the study intervention, that is confirmed by improvement on stopping and reappearance of the event on repeated exposure, and that could not be reasonably explained by the known characteristics of the participant's clinical state.

Possibly related: An adverse event that follows a reasonable temporal sequence from administration of the study intervention of that follows a known or expected pattern of response to the study intervention, but that could readily have been produced by a number of other factors.

Unrelated: The adverse event is clearly not related to the investigational procedure (i.e. another cause of the event is most plausible; and/or a clinically plausible temporal sequence is inconsistent with the onset of the event and the study intervention and/or a causal relationship is considered biologically implausible).

7.6 Expected AEs

Expected adverse events (AEs) will be captured through interviews at 12, 24 and 36 weeks, based on the Health Status Update questionnaire. The following are expected adverse events that have been listed in the informed consent form:

- Muscle soreness
- Fatigue
- Chest pain
- Breathing problems
- Cardiac event
- Fall (with or without injury)

7.7 Reportable AEs (RAEs)

Reportable AEs are events that have potential implications for participant safety and that require individual reporting. RAEs will be defined as events that fall into at least one of the following categories:

1. Serious adverse event (SAEs) - SAEs will be defined as any adverse event that results in death, is life threatening, or places the participant at immediate risk of death from the event as it occurred, requires or prolongs hospitalization, causes persistent or significant

disability or incapacity, results in congenital abnormalities or birth defects, or is another condition which investigators judge to represent significant hazards.

2. Unexpected AEs - An unexpected AE is defined as medical events that occur during study participation, but do not commonly occur in the study population and which are not listed in the informed consent document or study protocol.
3. AEs related or possibly related to the research intervention – defined as any AE which in the opinion of the principal investigator, the incident, experience or outcome more likely than not was caused by the procedures involved in the research.

Events that cannot be clearly defined as "reportable" will be discussed with the study physician and the PI to determine if they should be reported. All reportable events will be captured on an Adverse Event form which will be filed in the participant binder and then reported using the following guidelines.

7.8 Reporting of Events

The study PI has primary responsibility for the safety of participants as it relates to the study protocol. The study coordinator will be responsible for reviewing adverse events and assuring accurate and timely reporting of the adverse events. The co-investigators (including study physician) will review, evaluate and classify adverse events and provide follow-up for events until they are resolved. The PI will be responsible for reporting study-defined AEs and SAEs to the University of Pittsburgh institutional review board (IRB) according to their timeline and format.

8 INTERVENTION DISCONTINUATION

8.1 Interruption to Exercise Participation

Attendance will be taken at all exercise session and will be documented on the attendance sheet. Participants who miss three consecutive classes will be contacted by phone to determine the reason for the absences. Participants who miss three or more consecutive classes due to illness or injury will be required to obtain medical clearance from their physician prior to returning to the exercise class. Participants who miss three consecutive classes for other reasons (vacation, ill spouse, caregiving responsibilities, transportation etc.) will be encouraged to attend as many classes as possible.

8.2 Intervention Discontinuation

At any time, the study team may recommend discontinuation of any component of the intervention or intervention group of the study for any of the following reasons:

1. Compelling evidence from this or any other study of an adverse effect of the study intervention(s) that is sufficient to override the potential benefit of the interventions to the target population
2. Compelling evidence from this or any other study of a significant beneficial effect of the study intervention(s), such that it is continued denial to other study group(s) would be unethical
3. A very low probability of addressing the study goals within a feasible timeframe.

The research team may decide at any time to remove a participant from the research study if the team feels the participant is unsafe to continue. Participation in the research study will be discontinued if a participant's walking ability worsens; for example, if a subject who has osteoarthritis of the knees exhibits a significant increase in pain that negatively affects their walking ability. Participants can be removed from the study if they have unstable vital signs as a response to exercise. A participant can also be removed from the study if any new medical condition, injury or illness is discovered during the course of treatment that would make the participant unsafe to continue.

8.3 Voluntary Participation

The participants' participation in this research study is completely voluntary. The participant may withdraw, at any time, their consent for participation in this research study. Any identifiable research information recorded for, or resulting from, their participation in this research study prior to the date that they formally withdrew their consent may continue to be used and disclosed by the investigators. To formally withdraw consent for participation in this research study the participant should provide a written and dated notice of this decision to the principal investigator of this research study. If the participant withdraws from the intervention, study staff will ask permission to continue to follow the participant for follow-up assessment. If participation is discontinued for medical reasons and the participant is unable to complete the performance-based testing, all attempts will be made to obtain the self-reported outcomes.

9 STATISTICAL CONSIDERATIONS

9.1 General Design Issues

A randomized trial is needed to control for confounding factors that affect the outcome. There are no ethical issues regarding a randomized trial since both groups will be receiving an active exercise intervention delivered by trained personnel. We carefully considered the advantages and disadvantages of randomizing at the level of the facility and the resident. Given the amount of interaction that occurs between subjects within a facility, it is imperative that we conduct a cluster randomize trial, and randomize by facility to exercise programs. If we randomize at the resident, participants would discuss details of their intervention and cause cross-contamination between the intervention arms. Unlike a traditional trial in which participants are randomized as they are being recruited, a cluster randomized trial also affords the additional benefit of

examining the facility characteristics such as type (independent living/senior high rise) and size, and ensuring a balance in those characteristics is achieved by design rather than chance. Once the facilities are randomized to exercise program, we will then randomized within facility for delivery mode (i.e. subjects will be randomized to either an exercise leader or staff activity person led exercise program). Randomization for delivery mode will occur after baseline testing. We will use a commercially available high quality pseudo-random deviate generator such as that available in SAS® (SAS Institute, Inc., Cary, North Carolina) known to be free of serial correlations(56) to randomize facilities to the two arms in a 1:1 ratio, stratified by facility type and size. In addition, the proposed design reaps the advantages of a paired comparison for the sustainability hypothesis (Aim 2) as both exercise leaders and staff activity personnel will be delivering On the Move in the same set of facilities.

The main outcomes are self-reported function and disability (Late Life Function and Disability Index/LLFDI) and walking ability (6-minute walk test/6MWT and gait speed). We will also examine satisfaction, attendance rates and adverse event (falls, soft tissue injuries, muscle soreness, etc) rates.

9.2 **Sample Size**

We base our sample size justification on prior data from our RESTORE and PRIME pilot studies (see Part A. Background and Significance above), two-tailed tests conducted at the α=0.05 level, a conservative attrition rate of 10% between baseline and follow-up assessments, practical consideration of a class size of 10 participants for group exercise, computational techniques that match our study design and proposed analytical approach as much as possible within the constraints of already published methodologies and commercially available sample size and power software (PASS 2002®, Number Cruncher Statistical Systems, Inc., Kayesville, Utah), and ability to detect differences that correspond to published meaningful change criteria(57) or moderate Cohen's effect sizes of *d*=0.5.(58)

The numbers needed to enroll in order to detect statistical significance of intervention effect when delivered by an exercise leader (Aim 1) and staff activity personnel (Aim 2), are presented in **Table 3.** We take into account the clustering of participants by facility by assuming an intracluster correlation of 0.1 and a resulting design effect of 1.90 to appropriately inflate the sample size accordingly.(59) In summary, a total of 560 participants with 140 in each arm will allow us to detect statistical significance in intervention effects in all main outcomes with at least 80% statistical power.

Table 3. Number needed to enroll for detecting effects of intervention incorporating clustered design (x1.90), attrition (10%), and rounded up to class size of 10.

Outcome	Prior Information			Estimated Sample Size	
	Baseline Standard Deviation (SD)	SD in Baseline-Follow up Change	Meaningful Difference Targeted (Source)	Completers Needed Per Arm assuming No Clustering	Number Needed to Enroll Per Arm
LLFDI Overall Function	6.15	4.86	3.08 (*d*=0.5)	41	90
LLFDI Disability Frequency	6.35	4.76	3.18 (*d*=0.5)	37	80
Gait Speed (m/s)	0.13	0.20	0.10[65]	64	140
6MWD (m)	62.0	42.8	50[65]	13	30

The numbers needed to enroll in order to detect statistical significance of difference in gains attributable to *On the Move* when delivered by exercise leaders and staff activity personnel (sustainability hypothesis in Aim 2) are in **Table 4**. We use 95% statistical power (rather than the customary 80%) to reduce the likelihood of a type II error, thereby minimizing the probability of a finding in favor of sustainability being due to lack of statistical power rather than equivalence. We take into account the clustering of participants by facility assuming an intracluster correlation of 0.1, the advantages reaped by the pairing nature of clusters, and the resulting design effect of 0.90 to appropriately adjust the sample size.(60, 61) In summary, the sample size requirements for assessing the intervention effects above are also adequate for assessing the sustainability hypothesis with at least 95% statistical power.

Table 4. Number needed to enroll for establishing sustainability of *On the Move*, incorporating clustered design (x0.90), attrition (10%), and rounded up to class size of 10.

Outcome	Prior Information			Estimated Sample Size	
	Baseline Standard Deviation (SD)	SD in Baseline-Follow up Change	Meaningful Difference Targeted (Source)	Completers Needed Per Arm assuming No Clustering	Number Needed to Enroll Per Arm
LLFDI Overall Function	6.15	4.86	3.08 (*d*=0.5)	66	70
LLFDI Disability Frequency	6.35	4.76	3.18 (*d*=0.5)	60	60
Gait Speed (m/s)	0.13	0.20	0.10[65]	105	110
6MWD (m)	62.0	42.8	50[65]	21	30

9.3 **Data Analyses**

9.3.1 Overview

All statistical analyses will be performed or overseen by Dr. Perera using SAS® version 9 (SAS Institute, Inc., Cary, North Carolina) and Salford Predictive Miner® (Salford Systems, Inc., San Diego, California) based on the **intention-to-treat** philosophy. We will begin by summarizing data by arm and time point as well as pre- to post-intervention change using appropriate descriptive statistics for continuous (mean, standard deviation, median, range) and categorical (frequencies, percentages) to elicit information about general data quality and their distributional characteristics. Next, we will perform the modeling and inferential analyses to address the main hypotheses. First, the baseline participant characteristics will be compared between the two arms (see Avoidance of Bias below). Although no significant differences are expected, any significant differences will be noted and accounted for as covariates in the main analyses. Second, main analyses to address Aims 1-3 will be performed as outlined below. If residuals reveal violations of linear models assumptions, we will Box-Cox transform(62) the response variables. The two-step protected test approach will be used to control the experimentwise type I error rate from multiple outcomes, and multiple imputation(63, 64) will be used to account for any missing data in the main analysis. Third, we will perform the exploratory analyses to address Aim 4, using a data mining philosophy. Finally, we will perform a set of exploratory analyses to potentially extend our findings and generate new hypotheses, as well as a set of sensitivity analyses to assess the robustness of our findings.

9.3.2 Main Analysis for Aims 1-3

First, we will perform a multivariate Hotelling *t*-test to simultaneously compare the baseline to follow-up change in the three primary outcomes between the arms to protect the type I error rate from multiplicity. If significant, subsequent analyses will be performed without further multiplicity adjustment. If not, subsequent comparisons will be performed with a conservative Bonferroni correction at the $\alpha=0.05/4=0.0125$ level. This protected test approach has been recommended in the statistical literature(65) and used in other exercise intervention trials with multiple outcomes.(66)

Second, we will fit a series of linear mixed models(67) using the SAS® MIXED procedure with baseline to follow-up change in each of the continuous outcomes (LLFDI function/disability, walking ability, other measures of mobility performance) as the dependent variable; intervention arm (standard/*On the Move*), delivery mode (by exercise leader/staff activity personnel) and their interaction as fixed effects of interest; baseline value of outcome and any other measures found to be different between arms or deemed important as additional fixed effects covariates; and a facility random effect to account for greater similarity of participants from the same facility compared to different facilities and resulting non-independence of observations within facility (ie. clustering). We will construct appropriate means contrasts to estimate difference in gains in the two interventions when delivered by exercise leaders (Aim 1); difference in gains in the two interventions when delivered by staff exercise personnel (Aim 2 effectiveness hypothesis); and difference in gains attributable to *On the Move* intervention when delivered by exercise leaders and staff activity personnel. Statistical significance of the estimates will serve as formal tests hypotheses.

Third, we will fit a series of generalized estimating equations (GEE) models(68) using the SAS® GENMOD procedure with each of the dichotomous adverse events, adherence (21+ sessions or ≥90%) and satisfaction outcomes as the dependent variable; a binomial distribution for the outcome and a logit canonical link function; intervention arm, delivery model and their interaction as effects of interest; baseline value of outcome and any other measures found to be different between arms or deemed important as additional fixed effects covariates; and an exchangeable working correlation structure to account for clustering due to facility. We will appropriately construct contrasts to test hypotheses of differential proportions with adverse events based on intervention and delivery mode (Aim 3).

9.3.3 Aim 4 exploratory Analysis

We will perform the exploratory analyses to identify combinations of baseline predictors of treatment response and risks of participating in *On the Move* program. We do not anticipate differences in outcomes of On the Move program based on delivery mode (Aim 2), and thus propose to combine *On the Move* groups led by exercise leaders and staff activity personnel in the present analysis to maximize sample size and amount of information available for this analysis (140+140=280). In the unlikely event of differences due to delivery mode, we will stratify the Aim 4 analysis by delivery mode.

Adverse events and markers of response to treatment in walking ability have readily available dichotomous operational definitions based on simply presence/absence of an event or evidenced based criteria for having achieved meaningful change in gait speed (0.1+ m/s) and 6MWT (50m).(57) For such dichotomous markers of benefit and harm, we will fit a series of logistic regression models using the SAS® LOGISTIC procedure and classification tree models(69, 70) using Salford Predictive Miner® software with each measure of whether there was a benefit/harm from *On the Move* as the dichotomous response variable; and baseline physical, psychosocial and demographic characteristics as predictors. Logistic regression models are more efficient when associations are linear, and hard-to-discover higher order interactions and non-linearity and/or multicollinearities among predictors do not exist, while classification tree models are more efficient when they do exist. In terms of practically applying prediction criteria, results from logistic regression models require substituting values of predictors to a regression equation while classification tree model produces a flowchart that can be used as is in a clinical setting when a new individual is presented with known predictors. Thus we will use both methods to obtain areas under the receiver operator characteristic curve (AUROCC) to quantify predictive accuracy, and use the results from the method with a greater AUROCC. If the AUROCCs are not substantially different, we will favor the classification tree results due to ease of interpretation and utility in the clinical setting (see **Figure 2** for a hypothetical template for summarization of results). For logistic regression modeling, we will include all available baseline information simultaneously in the model as predictors, and use the backward elimination stepwise procedure to guard against model over-fitting to obtain a parsimonious model with a small number of most relevant predictors to facilitate interpretation, communication and clinical utility of the model. For the classification tree models, we will use all predictors available and use a minimum misclassification-complexity cost tree to achieve the same objectives. We will use the method of DeLong(71) to obtain the statistical significance of the improvement in predictive accuracy, as measured by AUROCC.

Our LLFDI function and disability outcomes do not have the same level of evidence based for creating intuitively appealing dichotomous measures of treatment response. Thus we will consider pre- to post-intervention change as a continuous variable, and perform analogous analysis to that for dichotomous outcomes but with appropriate statistical models. Specifically, we will fit standard multiple linear regression models using the SAS® REG procedure and regression tree models(69, 70) using Salford Predictive Miner®; and use proportion of explained variance (R^2) to quantify predictive accuracy.

9.3.4 Additional Exploratory/Sensitivity and Compliance/Dropout Analyses

Figure 2. Hypothetical template for summarization of Aim 4 results.

Figure 1. Predictors of treatment response using age, gait speed, and fear of falling. Area under ROC curve=0.797

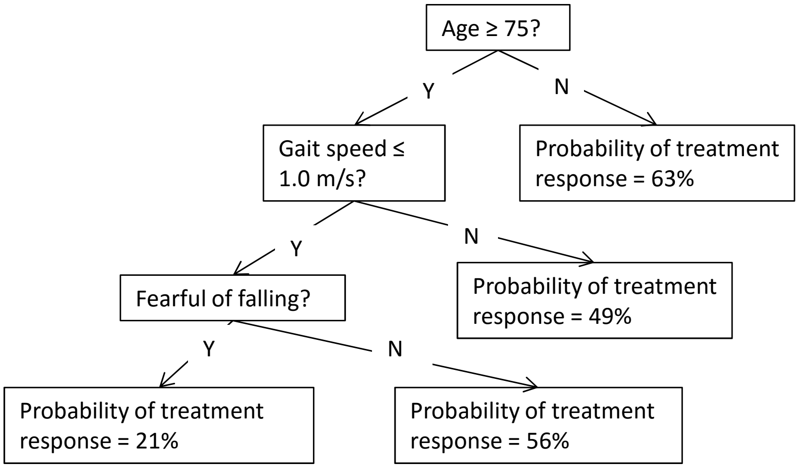

We will perform additional analyses to extend our findings, generate new hypotheses, assess robustness and potentially refine conclusions. They will include using an alternative threshold besides 90% to operationally define high level of adherence/compliance calculating proportion missing each session to describe the pattern of adherence/compliance over time; assessing intervention effects using as-treated instead of intention-to-treat philosophy; and alternative operational definitions of treatment response such as combinations of walking ability, function and/or disability, and reaching a published threshold such as 0.4(0.8) m/s in gait speed for limited(full) community ambulation.(72)

10 DATA COLLECTION AND QUALITY ASSURANCE

10.1 Data Collection Forms

Data collection will consist of paper forms. Data collected on paper forms will be entered into the electronic database by the research staff.

Screening and baseline data collection, which will occur prior to randomization, will be conducted by research staff trained in the outcomes and may include the study coordinator if necessary. Outcome assessments post-intervention will only be conducted by research staff trained in the outcomes and who are blinded to the intervention group assignment.

Participants' confidentiality will be protected in the data collection process. All study personnel are certified in Research Practice Fundamentals, Human Subjects Research Module. Consent forms and paper data collection forms will be stored in locked file cabinets. All computers are password protected. Only authorized team members will have access to personal information needed for tracking and informed consent.

10.2 **Data Management**

An electronic tracking system will monitor enrollment, track follow-up rates and the data entry process, providing up-to-date status reports. All completed data collection forms will be entered into a secure relational database located in a local network, and stored in a secure location. To improve accuracy, the data entry screens are identical in appearance to the paper forms. The data entry system includes automatic and routine data quality checks for out-of-range and extreme values, and automatic enforcement of skip patterns. In addition, functionality will be built in to the data entry system to facilitate double data entry and comparison of two versions so that discrepancies can be resolved against the authoritative paper forms. All screened subjects will be assigned unique subject identifiers that will appear on all data collection forms and files and serve as an index in database tables. The database will have access restricted to only those study personnel who need it and the level of access (read/write) will depend on the specific role. All files will be backed-up daily and archived weekly, including storage of back-up copies in an off-site location.

10.3 **Quality Assurance**

10.3.1 Intervention

Program monitoring: Periodically throughout the exercise program we will be videotaping the exercise sessions. These videos will be used primarily to monitor the quality and consistency of the exercise program and to further develop the training manual for the exercise instructors. The videos may be used to train future exercise instructors.

At each session, the exercise leaders and activity staff personnel will complete the exercise class log (see 9.3 above). Dr. VanSwearingen will review the exercise logs monthly to make sure the interventionists are following the protocol and progressing the exercise class appropriately. If deficiencies are notes, we will review the program with the interventionist and discuss potential modifications to the administration of the program.

10.3.2 Protocol Deviation Tracking

Protocol deviations may occur in the randomization process, exercise intervention protocols, the timing or completion of testing sessions, and in the completion of data forms. A Protocol Deviation Form has been developed for the study and will be completed, dated, and signed for each protocol deviation that may occur for each subject. This form will be kept in a folder for

protocol deviations. The protocol deviation will also be noted in the progress section of the subject's research record.

10.3.3 Subject Termination

If subject participation in the study is terminated for any reason (death, self-withdrawal, lost-to-follow-up, or change in health) a Study Termination form will be completed by the trial coordinator and placed in the subject's research record.

11 PARTICIPANT RIGHTS AND CONFIDENTIALITY

11.1 **Institutional Review Board (IRB) Review**

The study protocol, the informed consent document and any subsequent modifications will be reviewed and approved by the University of Pittsburgh IRB.

11.2 **Informed Consent Forms**

All potential participants will be adults (65 years of age or older) who are capable of providing direct consent for their participation in the study. Written informed consent will be obtained at the clinic screening visit prior to performing any of the clinic screening procedures. One of the study investigators will explain the study and the participant will be given a copy of the consent form to read. The consent form will describe the purpose of the study, the procedures to be followed, and the risks and benefits of participation. The investigator will answer any questions that the participant may have about the study. Finally, the participant will be asked to sign and date the consent form. The participant will be given a copy of the consent form for their records.

11.3 **Participant Confidentiality**

Research assessments and interventions are conducted in the community/activities room. This space includes several small tables in different portions of the room that can be used to privately conduct questionnaires and simple physical examination measures. Many of the walking assessments are done in the open area. Participants may be screened individually, which further protects their privacy. Participants are informed via the consent process that the treatment programs are conducted in groups. Although not completely private, this level of exposure to others during exercise is similar to what one might experience at a physical therapy appointment or during an exercise class at a public gym.

Participant's confidentiality will be protected in the data collection process. All personnel involved with the research have read and signed a Confidentiality statement. All study related data will be maintained in secure locked hard copy files and password protected computer files. To facilitate referring to our older adult subjects by name throughout all testing sessions and to minimize errors that could occur while multiple testers collect data on multiple participants in the same research space at the same time, data sets will not be de-identified. Subjects' names and emergency contact information will be maintained in both the hard copy and computer files, and subjects will be made aware of this during the informed consent process.

If data are used in scholarly presentations or journal articles, the investigators will protect the anonymity of individual participants and will report only aggregate data (eg group means) where appropriate. The Principal Investigator will review data confidentiality process monthly or as indicated with the research staff. The Investigators are all certified in Research Practice Fundamentals, Human Subjects Research Module.

11.4 Study Discontinuation

The study may be discontinued at any time by the IRB, the NIA, the OHRP, or other government agencies as part of their duties to ensure that research participants are protected.

12 STUDY TIMELINE

In this three year project we will conduct a single-blind cluster randomized intervention trial. The trial will be conducted in 28 facilities (ILFs and Senior High Rises) and will include 560 community-dwelling older adults. We will train 28 staff activity personnel and will conduct 56, 12 week group exercise sessions. The table below contains the timeline for all research activities.

Table - Project Timeline.

Research Activity	Year 1				Year 2				Year 3			
Hire and train research staff	X	X										
Develop manual of operations	X	X										
Assemble Advisory Board	X											
Train staff activity personnel			X	X	X	X	X	X				
Finalize data collection forms	X	X										
Construct database		X	X									
Recruitment			X	X	X	X	X	X				
Baseline testing			X	X	X	X	X	X				
Conduct exercise programs				X	X	X	X	X				
Post testing				X	X	X	X	X	X			
In-depth satisfaction interviews				X	X	X	X	X	X			
In-depth interviews of Community Advisory Board					X	X						
Data entry				X	X	X	X	X	X	X		
Analysis										X	X	
Review and interpret results										X	X	X
Dissemination												X

Meetings												
Advisory Board meetings	X		X		X		X		X		X	
Research staff	X	X	X	X	X	X	X	X	X	X	X	X
Data Safety Monitoring Meeting		X				X				X		

Year 1

In the first 6 months of funding, we will hire and train research staff, develop the manual of operations, assemble to Advisory Board, finalize the data collection forms, and construct the database. In the second 6 months of Year 1 we will initiate recruitment and baseline testing of research subjects. The group exercise programs will begin in a minimum of 4 facilities in months 10-12 of Year 1. The deliverables for Year 1 are outlined in the milestone schedule.

Year 2

At the beginning of Year 2 we will conduct the in-depth interviews of the Community Advisory Board members. The majority of the group exercises classes will be conducted in Year 2. We will continue to train the staff activity personnel (months 1-9 Year 2) as we include additional facilities in the research study. Our goal is that every quarter we would introduce the exercise program to 6 facilities and train the staff activity personnel to conduct the exercise program. In Year 2 we will recruit subjects to participate, baseline test the subjects, conduct the 12 week exercise program, and conduct the post testing. By the end of year 2, we will have trained 24 staff activity personnel to conduct the exercise program and completed forty-eight 12 week exercise sessions within 24 facilities. The deliverables for Year 2 are outlined in the milestone schedule.

Year 3

In Year three we will complete the exercise group sessions and complete all post testing. The majority of Year 3 will be dedicated to data entry, data analysis, review and interpretation of results and dissemination of research findings. See the milestone schedule for Year 3 deliverables.

13 <u>PUBLICATION OF RESEARCH FINDINGS</u>

Publications will be operationally defined as manuscripts for publications; abstracts for platform or poster presentation at scientific meetings and other professional meetings; slides for presentation at scientific and other meetings; doctoral dissertations; and master’s theses.

The goal of the publication policy is to encourage and facilitate publication of study results. The purposes of this policy are to ensure the following:

- On the Move publications will be of the highest scientific quality
- On the Move will be described in a consistent manner across publications
- Measures are reported in consistent ways across publications
- Proper acknowledgements are included
- Appropriate authorship credit is determined prior to submission of manuscripts for publication consideration.

Publications from On the Move will be overseen by the PI and Co-Investigators.

14 REFERENCES

1. Guralnik J, Ferrucci L, Simonsick E, Salive M, Wallace R**.** Lower extremity function in persons over the age of 70 years as a predictor of subsequent disability. New Engl J Med. 1995;332:556-61.
2. Cesari M, Kritchevsky S, Bauer D, Visser M, Rubin S, Harris T, et al. Prognostic value of usual gait speed in well-functioning older people--results from the Health, Aging and Body Composition Study. J Am Geriatr Soc. 2005;53:1675-80.
3. Guralnik J, Ferrucci L, Pieper C, Leveille S, Markides K, Ostir G, et al. Lower extremity function and subsequent disability: consistency across studies, predictive models, and value of gait speed alone compared with the short physical performance battery. J Gerontol Med Sci. 2000;55A:M221-M31.
4. Guralnik J, Simonsick E, Ferrucci L, Glynn R, Berkman L, Blazer D, et al. A short physical performance battery assessing lower extremity function: Association with self-reported disability and prediction of mortality and nursing home admission. J Gerontol. 1994;49:M85-M94.
5. Studenski S, Perera S, Patel K, Rosano C, Faulkner K, Inzitari M, et al. Gait speed and survival in older adults. JAMA. 2011;305(1):50-8.
6. Fried L, Bandeen-Roche K, Chaves P, Johnson B**.** Preclinical Mobility Disability Predicts Incident Mobility Disability in Older Women. Journal of Gerontology. 2000;55A(1):M43-M52.
7. Hoffman J, Ciol M, Huynh M, Chan L**.** Estimating transitions probabilities in mobility and total costs for Medicare beneficiaries. Arch Phys Med Rehabil. 2010;91:1849-55.
8. Guralnik J, Ferrucci L, Balfour J, Volpato S, Di I, A**.** Progressive versus catastrophic loss of the abilit to walk: Implications for the prevention of mobility loss. J Am Geriatr Soc. 2001;49:1463-70.
9. Brach J, Studenski S, Perera S, VanSwearingen J, Newman A**.** Gait variability and the risk of incident mobility disability. J Gerontol Med Sci. 2007;62A:983-8.
10. Hausdorff J, Rios D, Edelberg H**.** Gait variability and fall risk in community-living older adults: a 1-year prospective study. Arch Phys Med Rehabil. 2001;82:1050-6.
11. Judge J, Underwood M, Gennosa T**.** Exercise to improve gait velocity in older adults. Arch Phys Med Rehabil. 1993;74:400-6.
12. Mian O, Thom J, Ardigo L, Morse C, Narici M, Minetti A**.** Effect of a 12-month physical conditioning programme on the metabolic cost of walking in healthy older adults. Eur J Appl Physiol. 2007;100:499-505.
13. Brown M, Holloszy J**.** Effects of a low intensity exercise program on selected physical performance characteristics of 60- to 71-year olds. Aging (Milano). 1991;3:129-39.

14. Manini T, Marko M, VanArnam T, Cook S, Fernhall B, Burke J, et al. Efficacy of resistance and task-specific exercise in older adults who modify tasks of everyday life. J Gerontol Med Sci. 2007;62A:616-23.
15. Wolf S, O'Grady M, Easley K, Guo Y, Kressig R, Kutner M**.** The influence of intense Tai Chi training on physical performance and hemodynamic outcomes in transitionally frail, older adults. J Gerontol Med Sci. 2006;61A:184-9.
16. Helbostad J, Sletvold O, Moe-Nilssen R**.** Home training with and without additional group training in physically frail older people living at home: effect on health-related quality of life and ambulation. Clinical Rehabilitation. 2004;18:498-508.
17. Buchner D, Cress M, de L, BJ, Esselman P, Margherita A, Price R, et al. A comparison of the effects of three types of endurance training on balance and other fall risk factors in older adults. Aging Clin Exp Res. 1997;9:112-9.
18. Buchner D, Cress M, de L, BJ, Esselman P, Margherita A, Price R, et al. The effects of strength and endurance training on gait, balance, fall risk, and health services use in community-living older adults. Journal of Gerontology: Medical Sciences. 1997;52A(4):M218-M24.
19. Bean J, Herman S, Kiely D, Frey I, Leveille S, Fielding R, et al. Increased velocity exercise specific to task training: a pilot study exploring effects on leg power, balance, and mobility in community dwelling older women. J Am Geriatr Soc. 2004;52(5):799-804.
20. LIFE S, Investigators**.** Effects of a physical activity intervention on measures of physical performance: results of the Lifestyle Interventions and independence for elders pilot (LIFE-P) study. J Gerontol Med Sci. 2006;61A:1157-65.
21. Liu C, Latham N**.** Progressive resistance strength training for improving physical function in older adults. Cochrane Database of Systematic Reviews. 2009(3).
22. Nelson M, Rejeski W, Blair S, Duncan P, Judge J, King A, et al. Physical activity and public health in older adults: recommendation from the American College of Sports Medicine and the American Heart Association. Med Sci Sports. 2007;39(8):1435-45.
23. VanSwearingen J, Perera S, Brach J, Cham R, Rosano C, Studenski S**.** A randomized trial of two forms of therapeutic activity to improve walking: effect on the energy cost of walking. J Gerontol A Biol Sci Med Sc. 2009;64A:1190-8.
24. Nelson W**.** Physical principles for economies of skilled movements. Biol Cybernetics. 1983;46:135-47.
25. Daly J, Ruff R**.** Construction of efficacious gait and upper limb functional interventions based on brain plasticity evidence and model-based measures for stroke patients. The Scientific World Journal. 2007;7:2031-45.
26. Lay B, Sparrow W, Hughes K, O'Dwyer N**.** Practice effects on coordination and control, metabolic energy expenditure, and muscle activation. Human Movement Science. 2002;21:807-30.
27. Newman M, Dawes H, van d, Berg, M, Wade D, Burridge J, Izadi H**.** Can aerobic treadmill training reduce the effort of walking and fatigue in people with multiple sclerosis: a pilot study. Multiple Sclerosis. 2007;13:113-9.
28. Brooks V**.** The Neural Basis of Motor Control. New York: Oxford University Press; 1986.

29. Gentile A. Skill acquisition: action, movement, and neuromotor processes. In: JH C, RB S, J G, AM G, JM H, eds. Movement Sciences. 1 ed. Rockville: Aspen Publishers; 1987:93-154.
30. Polcyn A, Lipsitz L, Kerrigan C, Collins J. Age-related changes in the initiation of gait: degradation of central mechanisms for momentum generation. Arch Phys Med Rehabil. 1998;79:1582-9.
31. Capaday C. The special nature of human walking and its neural control. Trends in Neurosciences. 2002;25(7):370-6.
32. Alexander R. Walking made simple. Science. 2005;308:58-9.
33. Schmidt R. Organizing and Scheduling Prcatice. In: RA S, ed. Motor Learning and Practice: From Principles to Practice. Champaign, IL: Human Kinetics Books; 1991:199-225.
34. ACSM's Guidelines for Exercise Testing and Prescription. 5th ed. Baltimore, MD: Williams & Wilkins; 1995.
35. Haley S, Jette A, Coster W, Kooyoomijian J, Levenson S, Heeren T, et al. Late life function and disability instrument:II. Development and evaluation of the function component. J Gerontol. 2002;57A:M217-M22.
36. Jette A, Haley S, Coster W, Kooyoomijian J, Levenson S, Heeren T, et al. Late life function and disability instrument: I. Development and evaluation of the disability component. J Gerontol. 2002;57A:M209-M16.
37. Butland R, Pang J, Gross E, Woodcock A, Geddes D. Two-, six-, and 12-minute walking tests in reespiratory disease. BMJ. 1982;284:1607-8.
38. Lerner-Frankiel M, Vargas S, Brown M, Krusel L, Schoneberger W. Functional community ambulation: what are your criteria? Clinincal Management. 1986;6((2)):12-5.
39. Robinett C, Vondran M. Functional ambulation velocity and distance requirements in rural and urban communities. Phys Ther. 1988;68(9):1371-3.
40. Solway S, Brooks D, Lacasse Y, Tomas S. A qualitative, systematic overview of the measurement properties of the functional walk tests used in the cardioresepiratory domain. Chest. 2001;119:256-70.
41. Harada N, Chiu V, Damron-Rodriguez J, Fowler E, Siu A, Reuben D. Screening for balance and mobility impairment in elderly individuals living in residential care facilities. Physical Therapy. 1995;75(6):462-9.
42. Harada N, Chiu V, Stewart A. Mobility-related function in older adults: assessment with a 6-minute walk test. Arch Phys Med Rehabil. 1999;80:837-41.
43. Guyatt G, Sullivan M, Thompson P. The 6-minute walk: a new measure of exercise capacity in patients with chronic heart failure. Can Med Assoc. 1985;132:919-23.
44. Brach J, Perera S, Studenski S, Newman A. Reliability and validity of measures of gait variability in community-dwelling older adults. Arch Phys Med Rehabil. 2008;89:2293-6.
45. McAuley E, Mihalko S, Rosengren K. Self-efficacy and balance correlates of fear of falling in the elderly. J Aging Phys Activity. 1997;5:329-40.
46. Rosengren K, McAuley E, Mihalko S. Gait adjustments in older adults: Activity and efficacy influences. Psychology and Aging. 1998;13:375-80.
47. Newell A, VanSwearingen J, Hile E, Brach J. The modified gait efficacy scale: establishing the psychometric properties in older adults. Phys Ther. 2012;92:318-28.
48. Hess R, Brach J, Piva S, VanSwearingen J. Walking skill can be assessed in older adults: Validity of figure-of-8 walk test. Phys Ther. 2010;90:89-99.

49. Brach J, Perera S, VanSwearingen J, Hile E, Wert D, Studenski S. Challenging gait conditions predict 1-year decline in gait speed in older adults with apparently normal gait. Phys Ther. 2011;91:1857-64.
50. Gabell A, Nayak U. The effect of age and variability in gait. Journal of Gerontology. 1984;39(6):662-6.
51. Perera S, Brach J, Talkowski J, Wert d, Studenski S. Measuring stride time variability: estimating test-retest reliability and required walk length using bootstrapping. Program & Abstracts of the ISPGR 18th International Conference. 2007:55-6.
52. Brach J, Berlin J, VanSwearingen J, Newman A, Studenski S. Too much or too little step width variability is associated with a fall history in older persons who walk at or near normal gait speed. J Neuroengineering Rehabil. 2005;2(21).
53. Madden DJ, Whiting WL, Cabeza R, Huettel SA. Age-related preservation of top-down attentional guidance during visual search. Psychol Aging. 2004;19(2):304-9.
54. Kroenke K, Spitzer RL, Williams JB. The PHQ-9: validity of a brief depression severity measure. J Gen Intern Med. 2001;16(9):606-13.
55. Gardner M, Robertson C, Campbell A. Exercise in preventing falls and fall related injuries in older people: a review of randomized controlled trials. Br J Sports Med. 2000;34:7-17.
56. Meinert C. Clinical Trials. New York: Oxford University Press; 1986.
57. Perera S, Mody S, Woodman R, Studenski S. Meaningful change and responsiveness in common physical performance measures in older adults. J Am Geriatr Soc. 2006;54:743-9.
58. Cohen J. Satistical Power Analysis for the Behavioral Sciences. New York: Academic Press; 1977.
59. Eldridge S, Ashby D, Kerry S. Sample size for cluster randomized trials: effects of coefficient of variation of cluster size and analysis method. International Journal of Epidemiology. 2006;35:1292-300.
60. Vierron E, Giraudeau B. Sample size calculation for multicenter randomized trial: taking the center effect into account. Contemp Clin Trials. 2007;28(4):451-8.
61. Vierron E, Giraudeau B. Design effect in multicenter studies: gain or loss of power? BMC Medical Research Methodology. 2009;9:39.
62. Box G, Cox D. An analysis of transformations. Journal of the Royal Statistical Society-Series B. 1964;26:211-43.
63. Rubin D. Multiple Imputation for Nonresponse in Surveys: John Wiley and Sons; 1987.
64. Rubin D. Multiple imputation after 18+ years. Statistics in Medicine. 1991;14:1913-25.
65. Johnson D. Applied Multivariate Statistics. Belmont, CA: Duxbury Press; 1998.
66. Duncan P, Studenski S, Richards L, Gollub S, Lai S, Reker D, et al. Randomized clinical trial of therapeutic exercise in subacute stroke. Stroke. 2003;34(9):2173-80.
67. Milliken G, Johnoson D. Analysis of Messy Data Volume 1: Designed Experiments. New York: Van Nostrand Reinhold; 1992.
68. Diggle P, Liang K, Zeger S. Analysis of Longitudinal Data. Oxford: Clarendon Press; 1994.
69. Breiman L, Friedman J, Stone C, Olshen R. Classification and Regression Trees: CRC Press; 1984.

70. Strobl C, Malley J, Tutz G. An introduction to recursive partioning: rationale, application, and characteristics of classification and regression trees, bagging, and random forests. Psychological Methods. 2009;14(4):323-48.

71. DeLong E, DeLong D, Clarke-Pearson D. Comparing the areas under two or more correlated receiver operating characteristic curves: a nonparametric approach. Biometrics. 1988;44:837-45.

72. Schmid A, Duncan P, Studenski S, Lai S, Richards L, Perera S, et al. Improvements in speed-based gait classifications are meaningful. Stroke. 2007;38(7):2096-100.

ON THE MOVE: OPTIMIZING PARTICIPATION IN GROUP EXERCISE TO PREVENT WALKING DIFFICULTY IN AT-RISK OLDER ADULTS

Principal Investigator:

Jennifer S. Brach, PhD, PT

Supported by:

PCORI

CE-1304-6301

Version 2.0
October 29, 2015

Summary of Modifications to Protocol Version 1.0 for Version 2.0

**Version (date)**	**Section**	**Brief Summary of Modification**
Version 2.0 (12/03/2015)	Précis	We have updated the précis with a focus on Aim 1 and have specified the sample size by Aim. The sample size for Aim 1 is the same (n=280). We will continue enrolling subjects into Aim 2 until the sample size is met for Aim 1. We anticipate enrolling approximately 120 subjects in Aim 2.
Version 2.0 (12/03/2015)	Section 1.2 Secondary Objectives	Modified Aims 2 and 3 to make them more exploratory aims given our recent experience in the study and the barriers we have encountered with identifying and training facility staff to run the program.
Version 2.0 (12/03/2015)	Section 3 Study Design	• Added text to emphasize that comparisons of delivery mode (exercise leaders vs staff activity personnel) depend on the identification of staff activity personnel.<br>• Clarified that exercise leaders will determine if the staff activity personnel are trained to safely run the program.<br>• Clarified that if staff activity personnel is unable to lead the class, the second session will be led by an exercise leader.<br>• Removed names of specific individuals and replaced with a general description of the person responsible for the task.<br>• Updated #facilities from 28 to 34 and sample size in the staff activity personnel arms from 140 per group to 60 per group in the study figure.
Version 2.0 (12/03/2015)	Section 4 Selection and Enrollment of Participants	• Increased the number of sites<br>• Clarified that enrollment will continue until the sample size is met for Aim 1 (i.e. n=280)
Version 2.0 (12/03/2015)	Section 6.2.2 Baseline 2 Assessments	Clarified that if staff activity personnel is unable to lead the class, the second

		session will be led by an exercise leader.
Version 2.0 (12/03/2015)	Section 8.2 Intervention Discontinuation	Added that the research team may discontinue training of staff activity personnel or classes led by staff activity personnel if they believe the staff activity personnel is unsafe to lead the class.
Version 2.0 (12/03/2015)	Section 9.1 General Design Issues	Updated to reflect the exploratory nature of Aim 2
Version 2.0 (12/03/2015)	Section 9.2 Sample Size	• Made changes to indicate that sample size is focused on Aim 1.<br>• Aim 2 is now exploratory – and does not have a fixed sample size. We estimate that we will enroll approximately 120 participants into Aim 2 and have indicated the power for each outcome given the fixed sample size.
Version 2.0 (12/03/2015)	Section 9.3 Data Analysis	Updated analyses to reflect changes to the aims. Have separated the analysis into 2 main sections to reflect the change in Aim 2 to a more exploratory aim.
Version 2.0 (12/03/2015)	Section 12 Study Timeline	Updated timeline to reflect that recruitment will continue until March 2016 which will also extend baseline testing, intervention, and post-testing

TABLE OF CONTENTS

Page

PRÉCIS 7

STUDY TEAM ROSTER 8

1 Study objectives 9

1.1 Primary Objective 9
1.2 Secondary Objectives 9

2 BACKGROUND AND RATIONALE 9

3 STUDY DESIGN 12

4 SELECTION AND ENROLLMENT OF PARTICIPANTS 15

4.1 Inclusion Criteria 15
4.2 Exclusion Criteria 16
4.3 Study Enrollment Procedures 16

5 STUDY INTERVENTIONS 17

5.1 Overview 17
5.2 On the Move 17
5.3 Standard 18
5.4 Monitoring of vital signs 18
5.5 Program monitoring 18
5.6 Adherence Assessment 18

6 STUDY PROCEDURES 19

6.1 Schedule of Evaluations 20
6.2 Description of Evaluations 21
6.2.1 Baseline Assessments 21
6.2.2 Baseline 2 Assessments 23
6.2.3 Post-Intervention Assessment 24

7 SAFETY ASSESSMENTS 24

7.1 Expected Adverse Experiences 24

7.2 Minimizing Risk during Assessments and Interventions ... 25
7.3 Confidentiality. ... 25
7.4 Participant Education about Potential Risks ... 26
7.5 Adverse Events and Serious Adverse Events ... 26
7.5.1 Classifying Adverse Events (AE) ... 27
7.5.2 Severity ... 27
7.5.3 Expectedness ... 27
7.5.4 Relatedness ... 28
7.6 Expected AEs ... 28
7.7 Reportable AEs (RAEs) ... 28
7.8 Reporting of Events ... 29

8 INTERVENTION DISCONTINUATION ... 29

8.1 Interruption to Exercise Participation ... 29
8.2 Intervention Discontinuation ... 29
8.3 Voluntary Participation ... 30

9 STATISTICAL CONSIDERATIONS ... 31

9.1 General Design Issues ... 31
9.2 Sample Size ... 31
9.3 Data Analyses ... 34
9.3.1 Overview ... 34
9.3.2 Main Analysis for Aims 1and 3a ... 34
9.3.3 Aims 2, 3(b) and 4 exploratory Analyses ... 35
9.3.4 Additional Exploratory/Sensitivity and Compliance/Dropout Analyses ... 37

10 DATA COLLECTION AND QUALITY ASSURANCE ... 37

10.1 Data Collection Forms ... 37
10.2 Data Management ... 38
10.3 Quality Assurance ... 38
10.3.1 Intervention ... 38
10.3.2 Protocol Deviation Tracking ... 38
10.3.3 Subject Termination ... 39

11 PARTICIPANT RIGHTS AND CONFIDENTIALITY ... 39

11.1 Institutional Review Board (IRB) Review ... 39
11.2 Informed Consent Forms ... 39

11.3 Participant Confidentiality ... 39
11.4 Study Discontinuation ... 40
12 STUDY TIMELINE ... 40
13 PUBLICATION OF RESEARCH FINDINGS ... 41
14 REFERENCES ... 42

PRÉCIS

Community-dwelling older adults fear loss of independence and nursing home placement more than death. Walking difficulty often leads to loss of independence. Exercise is beneficial to physical and mental health and may prevent walking difficulty and promote independence. Recognizing the importance of exercise, senior housing facilities offer exercise programs to their residents. The exercise programs are often group-based, seated range of motion exercises that do not challenge the older adult; consequently participation rates and resident satisfaction are low. If the goal is to improve walking to promote independence than the exercise program should specifically target walking. Therefore, we developed a challenging, group exercise program entitled "On the Move" which focuses on the fundamentals of walking. In this research study we will determine if the On the Move program is better than a standard program at improving walking and promoting independence and if the same benefits can be obtained if the On the Move program is delivered by staff of the senior living facilities instead of an exercise leader. To answer these questions, community-dwelling older adults living in different Independent Living Facilities and Senior High Rises and attending senior centers will be randomly assigned to either the 12 week On the Move group exercise program or the standard group exercise program delivered by either an exercise leader or staff activity personnel. We will enroll 280 community-dwelling older adults into the exercise leader arm (Aim 1). We will enroll older adults into the staff activity personnel arm until the sample size is met for Aim 1. Given the number of barriers to identifying, training and maintaining the staff activity personnel (experienced after the study began) we will likely be unable to enroll 280 older adults into this arm. We will document barriers to identifying and training staff activity personnel. Participants' walking and reported ability to carry out everyday activities (functional ability) will be assessed before and after the 12 week program. We will also assess participant safety and satisfaction with the exercise program and instructor.

The findings from this research study will provide evidence for the value of the On the Move group exercise program and will better inform patient choices regarding participation in exercise programs. If successful in improving walking and promoting independence and acceptable to the older adult, the On the Move program could be incorporated into exercise programming for older adults in community centers, health clubs, and senior residences across the country.

STUDY TEAM ROSTER

<u>Principal Investigator:</u>

Jennifer S. Brach, PhD, PT
Bridgeside Point 1
100 Technology Drive
Pittsburgh, PA 15219-3130
Phone: 412-383-6533
Fax: 412-648-5970
jbrach@pitt.edu
Main responsibilities/Key roles: Oversees and is responsible for all aspects of the study

<u>Co-Investigators:</u>

**Deborah Brodine, MHA, MBA**<br>UPMC Community Provider Services<br>Forbes Tower Suite 10055<br>200 Lothrop St<br>Pittsburgh, PA 15213<br>Phone: (412) 647-0548<br>brodineds@umpmc.edu<br>Main responsibilities: Provider Stakeholder	**Sandra Gilmore, RN, MS**<br>UPMC Community Provider Services<br>101 Orchard Drive<br>Suite 104<br>Trafford, PA 15085<br>Phone: (412)380-8750<br>gilmoresl@upmc.edu<br>Main responsibilities: Provider Stakeholder, community liaison. Identify community sites.
**Neelesh Nadkarni, MD, PhD**<br>Division of Geriatric Medicine<br>Kaufmann Building, Suite 500<br>Pittsburgh, PA<br>Phone: (412) 692-2383<br>nkn3@pitt.edu<br>Main responsibilities: Study physician	**Subashan Perera, PhD**<br>Division of Geriatric Medicine<br>Kaufmann Building, Suite 500<br>Pittsburgh, PA<br>Phone: (412) 692-2365<br>Ksp9@pitt.edu<br>Main responsibilities: Randomization, data management and study statistician
**Jessie VanSwearingen, PhD, PT**<br>Bridgeside Point 1<br>100 Technology Drive<br>Pittsburgh, PA 15219-3130<br>Phone: 412-383-6533<br>jessievs@pitt.edu<br>Main responsibilities/Key roles: Quality control of the intervention	**Edmund Ricci, PhD**<br>207A Parran Hall<br>Graduate School of Public Health<br>University of Pittsburgh<br>130 DeSoto Street<br>Pittsburgh, PA 15261<br>Phone: 412-624-6393<br>emricci@pitt.edu<br>Main Responsibilities: Program evaluation

1 STUDY OBJECTIVES

1.1 Primary Objective

Aim 1: Compare the effects of the *On the Move* group exercise program to a standard program on self-reported function and disability and walking ability. *The On the Move program will produce greater gains in self-reported function and disability (Late Life Function and Disability Index/LLFDI) and walking ability (6-minute walk test/6MWT and gait speed) when delivered by an exercise leader.*

1.2 Secondary Objectives

Aim 2: When feasible to be delivered by staff activity personnel, explore the effectiveness of On the Move compared to a standard program; and sustainability compared to delivery by exercise leaders (when feasible). Explore barriers to identifying and training staff activity personnel. *On the Move delivered by staff activity personnel (when feasible) will produce gains in above outcomes that are greater than the standard program; and comparable to when delivered by an exercise leader.*

Aim 3: Explore the acceptability and the risks of the On the Move and standard exercise programs delivered by (a) exercise leaders and (b) staff activity personnel (when feasible). *On the Move will result in greater satisfaction and higher attendance rates than the standard program. Attendance rates and satisfaction will be similar for exercise leader and staff activity personnel led programs when feasible to recruit staff personnel. Adverse event (falls, soft tissue injuries, muscle soreness, etc.) rates during exercise will be similar between the two groups and the two facilitators when feasible to recruit staff personnel.*

Aim 4: Explore potential baseline predictors of benefit and risks of participation in *On the Move* program to facilitate informed patient decision making. *We will be able to identify combinations of baseline physical, psychosocial and demographic factors associated with each of the treatment response and adverse events outcomes.*

2 BACKGROUND AND RATIONALE

Disability is a common, costly problem in older adults. Walking difficulty in older adults contributes to loss of independence, higher rates of morbidity and increased mortality.(1),(2-5) Mobility loss is also a sentinel predictor of other disabilities that restrict independent living.(6) Compared to older adults without self-reported walking difficulty, those who developed mild walking difficulty over one year had higher healthcare costs (mean $1,128 per person). Extrapolated to the estimated 22% of older adults who develop walking difficulty annually, the cost to society is an additional 3.6 billion dollars per year.(7) Therefore, preventing or delaying the onset of walking difficulty might have a substantial impact on older adults' independence and their healthcare costs.

Exercise intervention studies have neglected to include disability outcomes. Difficulty walking is associated with reduced activity and participation and a loss of independence.(8),(9, 10) Exercise interventions for older adults have focused on improving walking as a means to reduce or delay physical disability.(11, 12) These exercise interventions have included strength, balance and endurance activities in order to reduce impairments and improve physiologic capacity for walking. These studies have resulted in only modest gains in walking ability (i.e. an approximate 5% increase in speed) with only one study reporting disability outcomes.(11, 13-21) Definitive evidence that exercise that improves walking also reduces disability is lacking; therefore the need for the ongoing Lifestyle Interventions and Independence for Elders (LIFE) study.

Exercise interventions fail to include an important component of exercise to improve walking, the timing and coordination of movement. National recommendations and interventions to prevent walking difficulty, such as the Lifestyle Interventions and Independence for Elders (LIFE) study have overlooked an important component of exercise that is critical for walking, the timing and coordination of movement.(20, 22) The ongoing Lifestyle Interventions and Independence for Elders (LIFE) study examines a standard walking endurance, strength, static balance and flexibility intervention on the prevention of disability in community-dwelling older adults. The LIFE pilot study, using the same intervention, demonstrated significant but only modest effects.(20) We have preliminary data to suggest that a novel exercise program that includes timing and coordination exercise is superior to a standard strength and endurance program for improving walking in older adults.(23)

Timing and coordination training improve walking in older adults. We conducted two pilot studies, involving contrasting subject groups, to examine the impact of a timing and coordination exercise program on walking. The first study (RESTORE) included older adults with slow (gait speed < 1.0 m/s) and variable gait and has been published.(23) The second study (PRIME) has just recently been completed. It included older adults with near normal gait speed (gait speed > 1.0 m/s) but with difficulty with aspects of the timing and coordination of walking (i.e. Figure of 8 test time > 8.0 s- see choice of outcomes section below for details of this measure). Participant retention for the 12 week post-tests was over 95%.

In the RESTORE study, 50 subjects (mean age 77.2±5.5 years, 65% women) were randomly assigned to either a standard exercise program (endurance, strength and typical static balance training) or a timing and coordination program, for one hour, 2 times per week for 12 weeks, with baseline and 12 week follow up assessments. Of the 50 who entered, 47 (94%) completed the study. Both groups increased gait speed (timing and coordination by 0.21 m/s and standard by 0.14 m/s). The timing and coordination group reduced the energy cost of walking 0.10±0.03mL/kg/m more than the standard group (p=0.0002), had a 9.8±3.5 point greater gain in reported walking confidence (i.e. Gait Efficacy Scale) than the standard group (p=0.008), had a 1.5±0.6 point greater reduction in gait abnormalities (i.e. GARSM) than the standard group (p=0.02), and had a 3.5±1.7 (p=0.04) point greater gain than the standard group in basic lower extremity function (LLFDI) and a 2.6±1.7 (p=0.12) point greater gain than the standard group in advanced LE function (LLFDI).(23)

In the PRIME study, 38 subjects (mean age 78.5±5.6 years, 65% women) were randomly assigned to either a standard endurance and strength exercise program or a timing and

coordination plus strengthening program, 2 times per week for 12 weeks, with assessments at baseline and immediately following the 12 week intervention. Preliminary analyses indicate that the timing and coordination group had greater improvements in gait speed, figure of 8 walk, and challenging gait tasks than the standard group. Both groups had improvements in the 6MWT ($p<0.05$); however, the timing and coordination group had marginally greater improvements ($p=0.14$). Only the timing and coordination group demonstrated improvement in self-reported disability and neither group decreased perceived walking difficulty (LLFDI – total function). However, this group had very high initial LLFDI function scores; reflecting low levels of baseline perceived walking difficulty, so may have been vulnerable to ceiling effects. Since our proposed sample will be more impaired, we anticipate that there is more room for change and that treatment group differences or declines of perceived walking difficulty will be detectable.

Our preliminary data demonstrate the benefit of timing and coordination training and support the concept that timing and coordination training provides something distinct from standard training, with potential effects on self-reported function, disability and mobility. Note that these pilot studies examined the effects of the timing coordination program delivered on a one on one basis. In the current proposal we will be testing the timing and coordination training delivered as a group program (i.e. On the Move group exercise program).

Exercise programs offered in senior housing are inadequate. Recognizing the importance of exercise for promoting physical and mental health many senior housing facilities offer exercise programs for the older adults. Though available, participation rates and participant satisfaction are low. Older adults feel the seated group exercise programs that are offered, are not challenging or beneficial so they stop participation. Providers, such as UPMC Senior Communities, are looking for viable alternatives. Older adults are interested in exercise interventions that will improve their mobility and help maintain their independence (focus group information). Exercise programs currently offered in senior living settings are inadequate because they 1) are often conducted in seated position and do not challenge the older adult and 2) exclude an important component of exercise that is critical to walking, the timing and coordination of movement. There is a need for a more challenging, evidence-based exercise program that is designed to improve walking and promote independence in older adults.

We developed a challenging, evidence-based group exercise program, On the Move, to improve walking in older adults. Though effective, an individualized, physical therapist led exercise program (described above as timing and coordination training) is not a cost effective model for the prevention of walking difficulty. A group exercise program is a cost efficient alternative which would likely promote adherence through socialization. Therefore, in collaboration with UPMC Community Provider Services, we developed a novel timing and coordination group-based exercise program entitled “On the Move”. The On the Move program differs from current group exercise programs in that 1) it contains timing and coordination exercises based on the biomechanics and motor control of walking (i.e. specificity of training), 2) the majority of the program consists of standing and walking exercises which challenge the older adult, and 3) it was developed with input from older adults.

UPMC Community Provider Services is interested in the sustainability of the exercise program. A main priority of our stakeholder, UPMC Community Provider Services, is the sustainability of

the exercise program. Often scientists conduct research within their facilities and when the research is over, the scientists move on to another project and the program does not continue. Recognizing this as an issue, we incorporated a sustainability component into our Aging Institute pilot described above. During the 12 week exercise program, the exercise leader is training the activity staff personnel to conduct the program. The activity staff personnel is currently assisting with the 12 week program. After 6 weeks of exercise classes we surveyed the activity staff personnel to determine her level of confidence in conducting the program. The activity staff responded that she was very confident that she could identify participants who were doing the exercises correctly and/or incorrectly and she was confident that she could lead the exercise program and progress the exercises. The activity staff personnel stated that a "cheat sheet" of listed exercises would be very useful when leading the exercise class. At the conclusion of the first 12 week exercise leader program, the staff activity personnel will start a new 12 week program with residents who were placed on the wait list.

Summary

The evidence-based On the Move program was developed based on our past research and with input from older adults. In our initial pilot study, we demonstrate that the program is feasible and acceptable to the older adult and that activity staff personnel have confidence in their ability to lead the program. In our current pilot we demonstrate our ability to engage management from other community sites and the ability to recruit from different populations. The next key step is to examine the benefits and risks of the On the Move program in a larger, more diverse sample of older adults and to assess the sustainability of the program. Specifically, we are interested in determining if the challenging group-based program improves mobility and prevents functional decline and disability without any increased risk to the older adult and if the activity staff personnel can lead the On the Move exercise program obtaining similar results to the exercise leader delivered program.

3 STUDY DESIGN

To address our research questions, we will conduct a cluster randomized single-blind two arm intervention trial to compare the effects on function, disability and mobility of a standard group exercise program and a novel *On the Move* group exercise program in 400 community-dwelling older adults who reside in independent living facilities and senior housing sites or who attend senior community centers. Group exercise classes are twice weekly for 12 weeks and will be delivered by exercise leaders and activity staff personnel. Function, disability and mobility are assessed at baseline and post intervention **(Figure 1).**

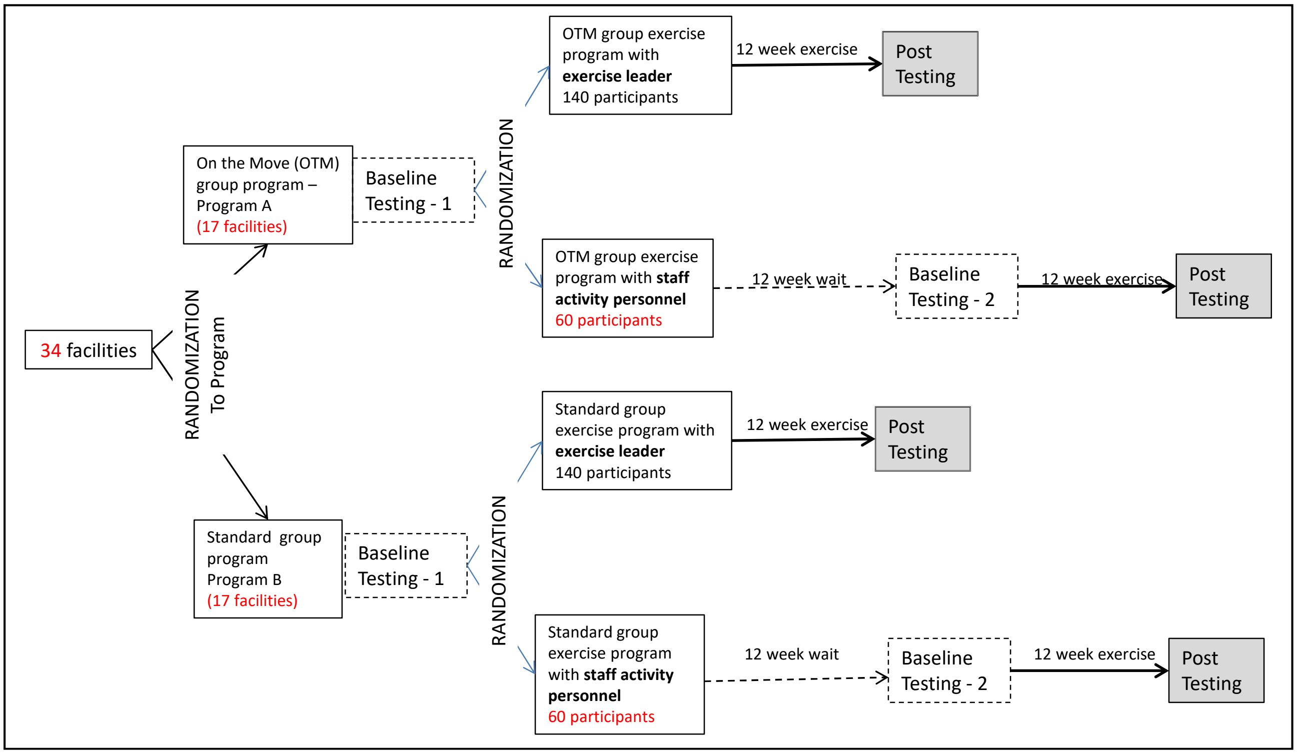

Figure 1.

Choice of comparators

There are two main comparisons being examined, 1) the type of exercise program (i.e. *On the Move* versus a standard group exercise program) and 2) the delivery mode (i.e. exercise leaders versus staff activity personnel), when a suitable staff activity person is available.

Exercise program comparison (Aim 1). The novel challenging *On the Move* exercise program will be compared to a standard group exercise program. Both exercise programs (*On the Move* and standard) will be group-based and led by a person. Our older adult participants have stressed the importance of having the exercise program led by a person instead of viewing a videotape. They feel the person is more enjoyable and they like the idea of the instructor providing feedback about their performance throughout the exercise class. Both programs will be delivered by trained exercise leaders or trained staff activity personnel (sustainability component; only when such a person is available). When a suitable staff activity person is not available, the program will be delivered by one of our exercise leaders. Class size will be set at a maximum of 10 participants. If there are more than 10 participants at any site, multiple classes will be scheduled. The frequency and duration of the programs are identical (50 minutes, 2 times a week for 12 weeks). From our past research we have determined that a frequency of 2 times per week for 12 weeks is acceptable to the older adult participants and is an adequate dose to obtain meaningful outcomes.(23) The main difference between the standard and the *On the Move* group exercise programs is the program content which is described below.

The <u>*On the Move* exercise program</u> is based on principles of motor learning that enhance "skill" or smooth and automatic movement control.(24-29) The program contains a warm-up (5 minutes), stepping patterns (15 minutes), walking patterns (15 minutes), strengthening exercises (10 minutes), and cool-down exercises (5 minutes). The warm-up and cool down contain gentle range of motion exercises and stretches for the lower extremities and trunk. The stepping and walking patterns are goal-oriented progressively more difficult patterns which promote the timing and coordination of stepping, integrated with the phases of the gait cycle.(25, 26, 28, 29) Conceptually, the exercise is intended to achieve its effects by shifting the center of pressure posteriolateral then forward, encouraging hip extension prior to stepping, loading the trailing limb, coordinating activation of the abductors of the soon to be swing leg with adductors of the stance limb, and shifting the center of pressure in medial stance to unload the stepping limb.(30-32) Progression is based on first separately increasing the speed, amplitude or accuracy of performance prior to undertaking a more complex task.(33) For example, the progression of stepping patterns is, 1) self-paced step forward and across, 2) increase stepping speed, 3) alternate side of stepping, 4) alternate forward with backward stepping. In the group class, exercises can be individualized by having some older adults use upper extremity support while others do not hold on during the exercises. Also, some subjects will step in all one direction while others will do the more challenging alternating left and right steps. Walking patterns incorporate patterns of muscle coordination and interlimb timing into walking. Walking patterns progress by altering speed, amplitude (e.g. narrowing oval width), or accuracy of performance (e.g. without straying from the desired path), and then to complex walking patterns involving walking past others and with upper extremity object manipulation tasks, such as carrying or bouncing a ball.(29) Walking and stepping patterns (i.e. timing and coordination training) were used in both of our pilot studies.(23) The strengthening exercises are conducted in sitting and standing and target the lower extremity muscles. The majority of the program will be conducted in standing (40 minutes) with only a small portion conducted in sitting (10 minutes). We have conducted two pilot exercise classes with older adults with varying levels of ability. We were able to successfully individualize the group program in that participants reported feeling both challenged and safe. Please see the appendix for example exercises and progressions.

The <u>standard group exercise program</u> is based on exercise programs that are currently being conducted at the facilities (i.e. standard of care). The operationally defined program contains a warm-up (5 minutes), upper and lower extremity strength and flexibility exercises (30 minutes), static balance exercises (10 minutes) and a cool-down (5 minutes). The majority of the program will be conducted in sitting (40 minutes) with only a small portion (10 minutes) conducted in standing. The active control group (i.e. standard exercise program) will aid in adherence and retention in that subjects who volunteer to participate in the study will be looking for exercise options.

Sustainability of the program: exercise leader and staff activity personnel comparison (Aim 2). The sustainability of the program will be evaluated by assessing the effectiveness of *On the Move* compared to a standard program when delivered by staff activity personnel and by comparing outcomes obtained by the *On the Move* program delivered by exercise leaders and staff activity personnel **(Figure 1)**. Exercise leaders are individuals with training and experience in administering exercise programs, such as exercise physiologists, physical therapists, physical therapy assistants, etc. Staff activity personnel are employees of the Independent Living

Faciltities and Senior High Rises that are involved in providing services to the residents. They could be activity directors, social workers, outreach coordinators, care coordinators, etc. These individuals are not specifically trained to deliver exercise interventions as part of their job. We will work with our community stakeholders and the building managers to identify staff activity personnel at each of the sites who would be involved in this research study. All exercise leaders and identified staff activity personnel will be trained in the delivery of the exercise program prior to leading any exercises classes. The staff activity personnel will participate in a one hour training sessions in which the rationale and the general format of the program will be explained. During these training sessions the activity staff personnel will participate in a sample exercise class. In addition to the 2 training sessions, the activity staff personnel will be able to observe the 12 week exercise session delivered by the exercise leader at their facility. They will be given the opportunity to observe all 24 sessions if they would like. In addition to training the exercise leaders and activity staff personnel, we will provide printed materials explaining the exercise program that we developed in our pilot work (see appendix). After training, the exercise leader will determine if the staff activity personnel is ready to lead the exercise class. The exercise leader will consider the following when determining if the individual is ready to lead the class: 1) attendance at training sessions, 2) attendance at exercise sessions, 3) interactions with research participants, and 4) understanding of the exercise program including all safety considerations. A study investigator (Dr. Brach or Dr. VanSwearingen or one of the exercise leaders) will meet with the staff activity personnel or email them periodically (i.e. during weeks 1, 3, 6, and 10 of the 12 week exercise program) to observe the intervention, monitor treatment fidelity, and to answer any questions. If the study team believes the staff activity person is not ready to lead a class safely and per protocol at a particular site, their class will also be led by an exercise leader.

4 <u>SELECTION AND ENROLLMENT OF PARTICIPANTS</u>

We will recruit participants from the UPMC Independent Living Facilities (ILFs), other Independent Living Facilities, Senior Housing sites, and Community Centers. Within UPMC there are eight different ILFs with over 700 residents. UPMC is affiliated with 32 different Senior Housing sites that house over 2,000 residents. We anticipate enrolling subjects from 10 ILFs and from approximately 24 of the Senior Housing sites for a total of approximately 34 sites. However, the number of participants per site will vary and we will continue adding sites and enrolling participants until the planned sample size is met for the primary Aim (i.e. n=280).

4.1 Inclusion Criteria

Participants must meet all of the following inclusion criteria to participate in the study.

1) 65 years of age or older
2) Resident of a UPMC ILF or Senior Housing site
3) Ambulate independently for household distances with or with a straight cane,
4) Usual gait speed greater than or equal to 0.60 m/s

4.2 Exclusion Criteria

Potential participants who meet any of the following exclusion criteria at baseline will be excluded from study participation.

1) Non English speaking,
2) Impaired cognition, defined as inability to follow 2 step commands or understand the informed consent process,
3) Plans to leave the area for an extended period of time over the next 4 months,
4) Progressive neuromuscular disorder such as Parkinson's or Multiple Sclerosis,
5) Any acute illness or medical condition that is not stable,
6) Inappropriate response to the 6 minute walk test (i.e. exercise heart rate >= 120 bpm, exercise SBP >= 220 or a drop in SBP > 10 mmHg, or DBP >= 110 mm Hg).

4.3 Study Enrollment Procedures

We will hold information sessions at each of the sites. The study PI (Dr. Brach) will visit each site and describe the study to the residents. Subjects who are interested in hearing more will be asked to place their name and phone number on a sign-up sheet. Research staff will then contact the subject to explain the study and conduct the initial phone screen if the subject is interested.

Subject eligibility will be determined from a phone screen and an in-person clinical screen. Researchers will contact potential participants by phone to determine their eligibility. After obtaining verbal consent, a structured screening questionnaire (see attached) will be administered to determine the presence and absence of the inclusion/exclusion criteria. Individuals who meet the criteria will be scheduled for an in-person screening visit that will take place at the ILF or senior housing site.

At the in-person screening visit, the first task will be to obtain informed consent for participation in the study. Once informed consent is obtained, the screening exam will take place. The screening exam will be conducted by trained research personnel (physical therapists, exercise physiologists, physical therapy assistants, or physical therapy students) who have experience working with older adults and conducting such screening measures. ACSM Guidelines regarding exercise participation will be available to all staff during testing. At all screening sessions at least 1 physical therapist will be available as a resource for the other research staff conducting screening procedures.

The purpose of the physical examination is to identify potential exclusion issues that are not identified by self-report. The exam includes a review of systems, current medications, vital signs, lower extremity range of motion and strength testing, and visual screening. A standard demographics questionnaire will be used to determine age, gender, race, marital status and work history in order to adequately describe the research participants. As a screening for exercise participation, all subjects will complete a six minute walk test (additional detail under experimental procedures). Participants will be asked to walk as far as they can in six minutes (without jogging or running) in a hallway. Participants are permitted to stop and rest during this test, if needed, and the number and duration of rest breaks are recorded. Blood pressure, heart rate and level of fatigue (using the rate of perceived exertion (RPE) scale) are recorded before

and after the test. Based on the ACSM guidelines, subjects who have an inappropriate exercise response (i.e. exercise heart rate >= 120 bpm, exercise SBP >= 220 or a drop in SBP > 10 mmHg, or DBP >= 110 mm Hg) will be referred to their primary care physician for clearance before they can be enrolled in the study.(34) Subjects who have an appropriate exercise response and are eligible based on the other screening criteria will then complete the outcome measures of function, disability and walking ability described below. All subjects will sign a “Liability Waiver Release for Participation in an Exercise Program” prior to starting the exercise program.

5 STUDY INTERVENTIONS

5.1 Overview

The exercise interventions will be delivered in 2 phases. The first phase or 12 week class will be delivered by the exercise leader. The second phase or 12 week class will begin once the first phase is completed and will be delivered by staff activity personnel. Participants who are randomized to the second phase class (i.e. delivered by staff activity personnel) will repeat the baseline testing prior starting the exercise class.

The facilities will be randomized (described above) to receive either the "On the Move" or a standard group exercise program. Both exercise programs (On the Move and standard) will be group-based and led by a person. Both programs will be delivered by trained exercise leaders or trained staff activity personnel (sustainability component). The frequency and duration of the programs are identical (50 minutes, 2 times a week for 12 weeks). From our past research we have determined that a frequency of 2 times per week for 12 weeks is acceptable to the older adult participants and is an adequate dose to obtain meaningful outcomes.(23) The main difference between the standard and the On the Move group exercise programs is the program content which is described below.

5.2 On the Move

The On the Move exercise program is based on principles of motor learning that enhance “skill” or smooth and automatic movement control.(24-29) The program contains a warm-up (5 minutes), stepping patterns (15 minutes), walking patterns (15 minutes), strengthening exercises (10 minutes), and cool-down exercises (5 minutes). The warm-up and cool down contain gentle range of motion exercises and stretches for the lower extremities and trunk. The stepping and walking patterns are goal-oriented progressively more difficult patterns which promote the timing and coordination of stepping, integrated with the phases of the gait cycle.(25, 26, 28, 29) Conceptually, the exercise is intended to achieve its effects by shifting the center of pressure posteriolateral then forward, encouraging hip extension prior to stepping, loading the trailing limb, coordinating activation of the abductors of the soon to be swing leg with adductors of the stance limb, and shifting the center of pressure in medial stance to unload the stepping limb.(30-32) Progression is based on first separately increasing the speed, amplitude or accuracy of performance prior to undertaking a more complex task.(33) For example, the progression of stepping patterns is, 1) self-paced step forward and across, 2) increase stepping speed, 3) alternate side of stepping, 4) alternate forward with backward stepping. In the group class,

exercises can be individualized by having some older adults use upper extremity support while others do not hold on during the exercises. Also, some subjects will step in all one direction while others will do the more challenging alternating left and right steps. Walking patterns incorporate patterns of muscle coordination and interlimb timing into walking. Walking patterns progress by altering speed, amplitude (e.g. narrowing oval width), or accuracy of performance (e.g. without straying from the desired path), and then to complex walking patterns involving walking past others and with upper extremity object manipulation tasks, such as carrying or bouncing a ball.33 Walking and stepping patterns (i.e. timing and coordination training) were used in both of our pilot studies.5 the strengthening exercises are conducted in sitting and standing and target the lower extremity muscles. The majority of the program will be conducted in standing (40 minutes) with only a small portion conducted in sitting (10 minutes). We have conducted two pilot exercise classes with older adults with varying levels of ability. We were able to successfully individualize the group program in that participants reported feeling both challenged and safe. Please see the appendix for example exercises and progressions.

5.3 Standard

The standard group exercise program is based on exercise programs that are currently being conducted at the facilities (i.e. standard of care). The operationally defined program contains a warm-up (5 minutes), upper and lower extremity strength and flexibility exercises (20 minutes), cardiovascular exercises (20 minutes) and a cool-down (5 minutes). The majority of the program will be conducted in sitting (>40 minutes) with only a small portion (<10 minutes) conducted in standing. The active control group (i.e. standard exercise program) will aid in adherence and retention in that subjects who volunteer to participate in the study will be looking for exercise options.

5.4 Monitoring of vital signs

Vital signs will be monitored before and after the exercise class as needed. Vitals signs will be monitored more at the beginning of the program, and at any time the participant reports or displays signs and symptoms (i.e. shortness of breath, lightheadedness, racing heart, etc). We will once again follow the ACSM guidelines for stopping of exercise.

5.5 Program monitoring

Periodically (approximately 1-2 times) throughout the exercise program we will be videotaping the exercise sessions. These videos will be used primarily to monitor the quality and consistency of the exercise program and to further develop the training manual for the exercise instructors. The videos may be used to train future exercise instructors.

5.6 Adherence Assessment

A roster of participants will be developed for each group exercise class. At the beginning of each class, attendance will be recorded by the exercise leader or staff activity personnel. Reasons for missed classes will be recorded when available. Attendance rate ([number of sessions attended

by the participant/total number of classes offered, i.e 24] X100%) will be calculated for each participant and will be the main indicator of adherence.

6 STUDY PROCEDURES

6.1 Schedule of Evaluations

Measure	Clinic Screen	Baseline 1<br>Pre-intervention	Baseline 2<br>Pre-intervention Activity staff group	12 week<br>Post-intervention
Demographics Questionnaire	X			
Physical exam screen (BP, strength, 2 step command)	X			
6 MWT	X		X	X
Screening gait speed	X			
Comorbidities Index		X		
Fall history		X	X	X
Anthropometric Measurements		X		
Gait Measures				
Gait speed – Zeno Walkway		X	X	X
Complex walk (Shumway-Cook)		X	X	X
SPPB		X	X	X
Figure 8		X	X	X
GES		X	X	X
Physical Function				
LLFDI		X	X	X
Global items		X	X	X
Potential confounders				
PHQ-9		X	X	X
Digit Symbol Substitution Test		X	X	X
Program Evaluation				
Satisfaction survey				X

6.2 **Description of Evaluations**

6.2.1 Baseline Assessments

If after the clinic screen the participant is eligible (i.e. meets all inclusion/exclusion criteria) they will undergo baseline testing. All baseline testing will be performed by research staff trained in the testing procedures. All testing will be completed at the ILFs and Senior Housing Sites.

Our main outcomes, function, disability, and walking ability are highly associated with independence and are extremely important to the older adult. Our primary measure of function and disability is the Late Life Function and Disability Instrument (LLFDI) and our main measures of walking ability are Six Minute Walk Test (6MWT) and gait speed. We will also examine confidence in walking (Gait Efficacy Scale), walking under challenging conditions (challenging gait tasks and figure of 8 walk), gait variability and the Short Physical Performance Battery (SPPB) as additional measures of walking ability. We will also collect a measure of cognition (Digit Symbol Substitution Test) and mood (PHQ-9).

6.2.1.1 Function and Disability

Late Life Function and Disability Instrument (LLFDI).(35, 36) Our primary function and disability outcome will be the LLFDI. The LLFDI is a pair of self-report measures targeted for assessing physical function and disability in older adults with acute or chronic problems, and designed to be more sensitive to change than similar measures. The two components of the LLFDI correspond to the activity (LLFDI – function) and participation (LLFDI – disability) components of the World health Organization's International Classification of Function, Disability and Health model. The LLFDI function component has 32 items in three dimensions, basic lower extremity (BLE), advance lower extremity (ALE) and upper extremity (UE) and the LLFDI disability component has 16 items representing two dimensions, frequency of performance and limitation in performance of life tasks. We've selected the LLFDI because 1) it measures both function and disability which are critical components of independence, 2) it includes a wide variety of life tasks in various social areas thus extending beyond the traditional focus of just activities of daily living, 3) the scale was designed with sufficient breadth of items and increments of rating in order to minimize ceiling and floor effects and maximize the scale's ability to detect change over time, and 4) it is a continuous outcome which gives us greater power than a dichotomous outcome to detect change over time. We will focus our analyses on the LLFDI function and disability dimension scores (i.e. BLE function, ALE function, UE function, disability frequency and disability limitation). We will also examine the disability domain scores (social role, personal role, instrumental role and management role) since they may provide insight into the impact of the disability on frequency of performance and perceived limitations.(36) The LLFDI function and disability scales have established known groups validity and the test-retest reliability is moderate to high for the disability component (ICCs range from 0.68 to 0.82) and extremely high for the function component (ICCs range from 0.91-0.98 for the dimensions). Scores range from 0-100; higher scores represent less difficulty and less disability.

6.2.1.2 Walking Ability

Six-Minute Walk Test (6MWT). One of the main walking ability outcomes is the Six-Minute Walk Test (6MWT) of distance walked (meters) in six minutes, including time for rest as needed.(37) We have selected the 6MWT because it is 1) a performance-based measure of walking ability and walking ability is an important component of independence, 2) an indicator of community ambulation (i.e. the ability to walk 300m in 6 minutes),(38, 39) 3) a continuous outcome which gives us greater power than a dichotomous outcome to detect change over time,(40) and 4) a widely used measure of mobility that is included in the NIH PROMIS project to establish measures of clinical assessment. The 6MWT has established psychometric properties, test-retest reliability (Pearson r=.95) in older adults,(41, 42) construct validity for graded exercise test and functional classification.(43) The Six-Minute walk test will be completed as part of the clinic screen (described earlier) and also used as an outcome measure. Participants will only complete the 6MWT once at baseline. Participants will be asked to walk as far as they can in six minutes (without jogging or running) in a hallway. Participants are permitted to stop and rest during this test, if needed, and the number and duration of rest breaks are recorded. Blood pressure, heart rate and level of fatigue (using the rate of perceived exertion (RPE) scale) are recorded before and after the test. Based on the ACSM guidelines, subjects who have an inappropriate exercise response (i.e. exercise heart rate >= 120 bpm, exercise SBP >= 220 or a drop in SBP > 10 mmHg, or DBP >= 110 mm Hg) will be referred to their primary care physician for clearance before they can be enrolled in the study.(34) Greater distance covered during six minutes is better.

Gait Speed. The second main walking ability outcome is gait speed. We have selected gait speed because it is a strong indicator/predictor of morbidity and mortality in the older adult.(2, 3, 5) Gait speed is assessed in usual walking with an instrumented walkway. After explanation, the participant completes 2 practice walks to become accustomed to walking on the walkway. The subject then completes 4 passes at their usual, self-selected walking speed. Gait speed will be averaged over the 4 passes. The test-retest reliability of gait speed measured using instrumented walkways by ICC is 0.98.(44) A faster speed is better.

6.2.1.3 Additional Mobility Measures

Gait Efficacy Scale. In order to determine if changes in walking ability are associated with changes in confidence in walking, confidence will be assessed using the Gait Efficacy Scale.(45-47) The items include a range of gait activities such as walking over different surfaces, up and down curbs, and negotiating stairs. Each item has a 10 point Likert scale scoring option, with the total score for the 10 items, ranging from 0-100. A higher score represents greater confidence.

Figure of 8 Walk.(48) The Figure of 8 Walk was designed to measure motor skill in walking. The test involves walking a figure of eight pattern about two markers placed 5 feet apart. Performance is scored based on the time to complete the figure 8 walk and the number of steps. Faster and fewer steps are better.

Challenging Gait Tasks. Challenging gait tasks are used to examine an individual’s ability to adapt their gait to different environmental conditions.(49)53 Subjects will complete two, 12

meter trials of each challenging condition, obstacle, curved path, and narrow path.(49) The time to complete each task, averaged over two trials, is the summary indicator of gait during challenging tasks. In a sample of 40 community-dwelling older adults, the 1-week test re-test reliability of the timed measures of challenging gait ranged from ICC = 0.70 to 0.94. The marginal additional time for completing each challenging task compared to usual gait is the main indicator and lower marginal cost is better.

Gait Variability. Gait variability, defined as fluctuations in gait characteristics from one step to the next,(50) is an important indicator of impaired mobility in older adults.(9) Gait variability is quantified using established measures of temporal and spatial gait characteristics including stance time, step length, and step width. Variability will be calculated as the standard deviation of the set of steps recorded over 4 passes on the instrumented walkway (described above). Approximately 32 steps will be collected from 4 passes on the mat which will be more than adequate to achieve a stable measure of gait variability. Our prior work has shown that 20 steps are sufficient to achieve a reliability of 0.75 and 30 are sufficient for 0.80.(51) In general, lower variability is better although there are exceptions.(9, 52)

Short Physical Performance Battery (SPPB). The SPPB of lower extremity function was designed and used in the Epidemiologic Studies of the Elderly (EPESE) to assess lower-extremity function of individuals 65 years of age and older. It combined measures of gait speed, balance, and timed chair rise to develop the SPPB for lower-extremity function. The three components are: gait speed over 4-meters, standing balance and chair stands are timed and these times are converted to scores from 0-4 (0=unable, 4=fastest time) for each component. Total scores on the Battery range from 0-12, ranges defined for relative risks of disability.(4)

6.2.1.4 Cognition and Mood

Digit Symbol Substitution Test.(53)A measure of processing speed, the Digit Symbol Substitution test will be used to gather information about both motor and cognitive processing speed. The DSST is a paper and pencil task from the WAIS III that provides normed measures of both motor and cognitive processing speed. The DSST has been widely used in studies of physical and cognitive performance of older adults.

Patient Health Questionnaire (PHQ-9).(54) The PHQ-9 is the 9 item depression scale of the Patient Health Questionnaire. It asks participants to describe how they have been feeling over the past 2 weeks. It has been used successfully in older adults.

6.2.2 Baseline 2 Assessments

Individuals who are randomized to the second exercise session taught by the staff activity personnel (or by the exercise leader if staff person not ready to lead it) will complete a second baseline assessment prior to initiating the exercise class. This will be approximately 12 weeks after the initial baseline assessment. Baseline 2 Assessment will be identical to Baseline testing.

6.2.3 Post-Intervention Assessment

At the completion of the 12 week exercise program, all participants will undergo a post-intervention assessment. The assessment will include all measures from the baseline assessment (LLFDI, 6MWT, gait speed, Gait Efficacy Scale, walking under challenging conditions (challenging gait tasks and figure of 8 walk), gait variability, SPPB, DSST, PHQ-9. Post-intervention testing will be completed at the sites (ILFs and senior housing sites) by research personnel masked to the intervention group and trained in the testing procedures.

In addition, we will also assess participant satisfaction. Participant satisfaction will be assessed using in-depth interviews and a satisfaction survey. In-depth phone interviews will be used to assess satisfaction in a subsample of participants (approximately 20%). We will randomly select a diverse sample of older adults representing the different intervention arms (On the Move, Standard, exercise leader, activity staff personnel) and the different sites (ILF and senior housing). We selected in-depth interviews instead of focus groups because we are more likely to draw people out, they are less intimidated, and more likely to be truthful. The interview will be developed by The Evaluation Institute under the direction of Dr. Edmund Ricci (Co-Investigator) and with input from the other investigators and stakeholders (Community Advisory Board). The interview will include a mixture of closed and open-ended questions. Sample topics to be included in the interviews include perceived benefits and risks, satisfaction with the program, facilities, and instructor, and amount of individualized instruction. The interview will be pilot tested and modified accordingly prior to administration in the study. Pilot testing will ensure that the questions are understandable to our target population and that we are obtaining the information and feedback that we hope to obtain.

Satisfaction will also be assessed by surveys in all participants at the conclusion of the exercise program. The satisfaction survey includes five items measured on a Likert scale and three yes/no questions. The Likert items will assess degree of satisfaction with various components of the exercise program (i.e. the exercises, instructor, music, space, and the overall program). We will also use a series of yes/no questions to determine if the program has meet the participants' expectations, if they feel they have benefited from the program, and if they would recommend the program to others.

7 <u>SAFETY ASSESSMENTS</u>

7.1 Expected Adverse Experiences

1. Major risks such as a cardiac event or a fall are expected to be rare – expected to occur in less than 1% of people (less than 1 out of 100 people).(55) Gardner et al, 2000,(55) reviewed controlled clinical trials of exercise interventions for older adults at-risk for falling. No cardiac events or falls were reported in the 12 clinical trials reviewed. The at-risk older adults in the studies reviewed have slightly poorer physical performance than the older persons we expect to recruit for our study. In our recent MOBILE study, a 1 year cohort study of 120 older adults participating in 3 clinic visits over a 1 year period there were no cardiac events or falls reported. In all conditions of testing in which the

participant is standing and/or walking (eg conditions with a potential risk for falling), the participant will be directly supervised by the trained tester. The trained tester is present for all testing sessions. We expect this level of supervision reduces the risk of falling to an even greater degree.

2. Less severe risks of participation such as such as muscle soreness, fatigue, or minor sprains or strains with each assessment, are expected to be infrequent – expected to occur in 1-10% of people (1-10 out of 100 people). Gardner et al, 2000,(55) reviewed controlled clinical trials of exercise interventions for older adults at-risk for falling, finding reports of such side effects of the intervention reported in only 4 of the 12 studies reviewed. The side effects were not a reason for dropout from the study and were described as soreness or musculoskeletal symptoms, but no injuries.
3. There is a rare risk that confidentiality could be breached. All of the research records will be kept in a locked file cabinet and/or password protected files. All of the investigators and staff that assist with the management of the files are trained in the privacy and confidentiality regulations that govern research.

7.2 Minimizing Risk during Assessments and Interventions.

All assessments approved in this study are considered to be a part of everyday clinical practice. We have minimized risks we believe by applying usual safeguards for the assessment of gait. Assessment side effects, such as muscle soreness, fatigue, or minor sprains or strains with each assessment, will be recorded by the physical therapist and monitored by Dr. Brach, the Principal Investigators in consultation with the physician investigator, Dr. Nadkarni. Based upon the existing literature and our own clinical experience, we anticipate the frequency of these side effects to be extremely low.(55)

We will maximize the safety of our subjects with the following procedures.

1. Individuals with absolute contraindications to testing will be excluded based on the inclusion/exclusion criteria.
2. Testing will be carefully monitored by trained testers and will be adjusted according to the American College of Sports Medicine Guidelines for Exercise Testing and Prescription.(34)
3. In all conditions of testing in which the participant is standing and/or walking (eg conditions with a potential risk for falling), the participant will be directly supervised by the trained tester.

7.3 Confidentiality.

Participant’s confidentiality will be protected in the data collection process. All personnel involved with the research have read and signed a Confidentiality statement, and approval is being obtained from the University of Pittsburgh Biomedical Institutional Review Board. Consent forms and data collection forms that identify the participant by name will be stored in a locked cabinet. All computers are password protected. If the data are used in scholarly presentations or journal articles, the investigators will protect the anonymity of individual

participants and will report only aggregate data (eg group means) where appropriate. The Principal Investigator will review data confidentiality processes monthly or as indicated with the project staff. The Investigators are all certified in Research Practice Fundamentals, Human Subjects Research Module**.**

7.4 Participant Education about Potential Risks

Potential risks associated with study-related activities and interventions will be explained to each participant by trained study personnel during the informed consent process. Each participant will be instructed to report the occurrence of an AE to appropriate study staff at scheduled data collection times, to PTs administering the intervention, or spontaneously at any other time. Participants also will be encouraged to report concerns about the safety of participating in the study to any research staff.

7.5 Adverse Events and Serious Adverse Events

If an adverse event occurs during testing or intervention, staff or activity personnel will assess the situation and provide immediate assistance to the participant if necessary. If a medical emergency should occur, staff will immediately contact 911. They will describe the incident to the 911 operator and provide their location. They will also contact the facility director to make them aware of the situation. In each testing/exercise space, we will post a copy of the emergency plan which will include the number to call (911) and the address of the facility. All staff will be informed of the emergency procedures prior to starting the interventions and they will be reviewed every 6 months. Once the emergency situation is under control, the staff will contact the study PI to notify them of the situation. The PI will report unexpected or adverse events in accordance with the University of Pittsburgh IRB guidelines. As a group the PI and Co-Is review the adverse events to determine if they are expected vs unexpected and serious vs not serious. All events are reported to the irb who will also review the events. The study coordinator (Ms. Betts), study biostatistician (Dr. Subashan Perera) and study physician (Dr. Neelesh Nadkarni) who are unblinded will track the events to determine if there are an unequal number of events between the groups.

A clinical complication form will be completed any time an incident (accident, injury, illness, problems with medication, etc.) occurs or a subject reports an episode. Complications will be categorized as study related, possibly study related, or not study related. Any complications that are categorized as either study related or possibly study related will be reported to the IRB per protocol if the event is an unexpected event, and/or is of serious or moderate to severe in nature as defined by the IRB. A separate clinical complication form will be completed for each incident that may occur in each subject. The form will be kept in the subject’s research record.

7.5.1 Classifying Adverse Events (AE)

An AE is any unfavorable or unintended medical occurrence in a human study participant that has taken place during the course of a research project, including any abnormal sign, symptom, or disease, whether or not related to participation in the research.

For the purposes of this study, any event that meets the criteria for a severe adverse event (SAE), is unexpected, or results in injury to the participant while he/she is under the supervision of study related personnel will be classified as a reportable adverse event (RAE). Adequate review, assessment, and monitoring of RAEs require they be classified as to severity, expectedness, and potential relatedness to the study intervention.

7.5.2 Severity

The following guidelines will be used to determine level of severity:

Mild: Awareness of signs and symptoms, but easily tolerated and causing no loss of time from normal activities. No specific medical attention is required.

Moderate: Discomfort enough to cause a low level of inconvenience or concern to the participant and may interfere with daily activities. Symptoms may require minimal, local or noninvasive medical intervention.

Severe: Events interrupt the participant's normal daily activities and are usually incapacitating. Significant symptoms may require hospitalization or invasive medical intervention.

Life-threatening/Disabling: Events that may involve acute, life-threatening metabolic or cardiovascular complications (such as circulatory failure, hemorrhage, sepsis) or life – threatening physiological consequences. Intensive care or emergent invasive procedure is required.

Death: Causing death.

Severity is not synonymous with seriousness. A severe headache is not necessarily an RAE. However, mild chest pain may results in a day's hospitalization and thus would be classified as a RAE.

7.5.3 Expectedness

AEs will be assigned as to whether they were expected or unexpected based on current knowledge. Categories are defined as follows:

<u>Expected:</u> An AE that is anticipated on the basis of prior experience with the intervention under investigation; an event that can be attributed to the underlying condition of the participant being studied; or an event that can be attributed to the patient population being studied (see section---Expected AEs). Expected AEs are captured in a standardized way by study personnel.

Unexpected: An AE that was not anticipated on the basis of prior experience with the underlying intervention under investigation; an event that can be attributed to the underlying condition of the participant being studied; or to the patient population being studied or an expected event whose frequency or severity exceeds what is anticipated. Unexpected events are reportable.

7.5.4 Relatedness

The PI in consultation with the Co-Investigators and an independent safety monitor will determine the degree to which RAEs are related to study procedures using the criteria below.

Definitely related: The adverse event is clearly related to the investigational procedure – i.e., an event that follows a reasonable temporal sequence from administration of the study intervention, follows a known or expected pattern of response to the study intervention, that is confirmed by improvement on stopping and reappearance of the event on repeated exposure, and that could not be reasonably explained by the known characteristics of the participant's clinical state.

Possibly related: An adverse event that follows a reasonable temporal sequence from administration of the study intervention of that follows a known or expected pattern of response to the study intervention, but that could readily have been produced by a number of other factors.

Unrelated: The adverse event is clearly not related to the investigational procedure (i.e. another cause of the event is most plausible; and/or a clinically plausible temporal sequence is inconsistent with the onset of the event and the study intervention and/or a causal relationship is considered biologically implausible).

7.6 Expected AEs

Expected adverse events (AEs) will be captured through interviews at 12, 24 and 36 weeks, based on the Health Status Update questionnaire. The following are expected adverse events that have been listed in the informed consent form:

- Muscle soreness
- Fatigue
- Chest pain
- Breathing problems
- Cardiac event
- Fall (with or without injury)

7.7 Reportable AEs (RAEs)

Reportable AEs are events that have potential implications for participant safety and that require individual reporting. RAEs will be defined as events that fall into at least one of the following categories:

1. Serious adverse event (SAEs) - SAEs will be defined as any adverse event that results in death, is life threatening, or places the participant at immediate risk of death from the event as it occurred, requires or prolongs hospitalization, causes persistent or significant

disability or incapacity, results in congenital abnormalities or birth defects, or is another condition which investigators judge to represent significant hazards.

2. Unexpected AEs - An unexpected AE is defined as medical events that occur during study participation, but do not commonly occur in the study population and which are not listed in the informed consent document or study protocol.
3. AEs related or possibly related to the research intervention – defined as any AE which in the opinion of the principal investigator, the incident, experience or outcome more likely than not was caused by the procedures involved in the research.

Events that cannot be clearly defined as "reportable" will be discussed with the study physician and the PI to determine if they should be reported. All reportable events will be captured on an Adverse Event form which will be filed in the participant binder and then reported using the following guidelines.

7.8 Reporting of Events

The study PI has primary responsibility for the safety of participants as it relates to the study protocol. The study coordinator will be responsible for reviewing adverse events and assuring accurate and timely reporting of the adverse events. The co-investigators (including study physician) will review, evaluate and classify adverse events and provide follow-up for events until they are resolved. The PI will be responsible for reporting study-defined AEs and SAEs to the University of Pittsburgh institutional review board (IRB) according to their timeline and format.

8 INTERVENTION DISCONTINUATION

8.1 Interruption to Exercise Participation

Attendance will be taken at all exercise session and will be documented on the attendance sheet. Participants who miss three consecutive classes will be contacted by phone to determine the reason for the absences. Participants who miss three or more consecutive classes due to illness or injury will be required to obtain medical clearance from their physician prior to returning to the exercise class. Participants who miss three consecutive classes for other reasons (vacation, ill spouse, caregiving responsibilities, transportation etc.) will be encouraged to attend as many classes as possible.

8.2 Intervention Discontinuation

At any time, the study team may recommend discontinuation of any component of the intervention or intervention group of the study for any of the following reasons:

1. Compelling evidence from this or any other study of an adverse effect of the study intervention(s) that is sufficient to override the potential benefit of the interventions to the target population
2. Compelling evidence from this or any other study of a significant beneficial effect of the study intervention(s), such that it is continued denial to other study group(s) would be unethical
3. A very low probability of addressing the study goals within a feasible timeframe.

The research team may decide at any time to remove a participant from the research study if the team feels the participant is unsafe to continue. Participation in the research study will be discontinued if a participant's walking ability worsens; for example, if a subject who has osteoarthritis of the knees exhibits a significant increase in pain that negatively affects their walking ability. Participants can be removed from the study if they have unstable vital signs as a response to exercise. A participant can also be removed from the study if any new medical condition, injury or illness is discovered during the course of treatment that would make the participant unsafe to continue.

The research team may decide at any time to discontinue training of a staff activity person if the teams feels the staff activity person is unsafe to continue. If training of a staff activity person is discontinued, an exercise leader will take over as instructor for the exercise class to keep our obligation of the exercise class to the community. If such a change becomes necessary after the class has begun, we will exclude that particular class from all analyses. If such a change becomes apparent before the class begins, and the entire class is taught by an exercise leader, we will include the class in Aim 1 analysis.

8.3 Voluntary Participation

The participants' participation in this research study is completely voluntary. The participant may withdraw, at any time, their consent for participation in this research study. Any identifiable research information recorded for, or resulting from, their participation in this research study prior to the date that they formally withdrew their consent may continue to be used and disclosed by the investigators. To formally withdraw consent for participation in this research study the participant should provide a written and dated notice of this decision to the principal investigator of this research study. If the participant withdraws from the intervention, study staff will ask permission to continue to follow the participant for follow-up assessment. If participation is discontinued for medical reasons and the participant is unable to complete the performance-based testing, all attempts will be made to obtain the self-reported outcomes.

9 STATISTICAL CONSIDERATIONS

9.1 General Design Issues

A randomized trial is needed to control for confounding factors that affect the outcome. There are no ethical issues regarding a randomized trial since both groups will be receiving an active exercise intervention delivered by trained personnel. We carefully considered the advantages and disadvantages of randomizing at the level of the facility and the resident. Given the amount of interaction that occurs between subjects within a facility, it is imperative that we conduct a cluster randomize trial, and randomize by facility to exercise programs. If we randomize at the resident, participants would discuss details of their intervention and cause cross-contamination between the intervention arms. Unlike a traditional trial in which participants are randomized as they are being recruited, a cluster randomized trial also affords the additional benefit of examining the facility characteristics such as type (independent living/senior high rise) and size, and ensuring a balance in those characteristics is achieved by design rather than chance. Once the facilities are randomized to exercise program, we will then randomized within facility for delivery mode (i.e. subjects will be randomized to either an exercise leader or staff activity person led exercise program). Randomization for delivery mode will occur after baseline testing. We will use a commercially available high quality pseudo-random deviate generator such as that available in SAS® (SAS Institute, Inc., Cary, North Carolina) known to be free of serial correlations(56) to randomize facilities to the two arms in a 1:1 ratio, stratified by facility type. In addition, the proposed design reaps the advantages of a paired comparison for the exploratory hypothesis (Aim 2) as both exercise leaders and staff activity personnel will be delivering On the Move in the same set of facilities, where feasible to train a staff person ready to lead the class safely and per protocol.

The main outcomes are self-reported function and disability (Late Life Function and Disability Index/LLFDI) and walking ability (6-minute walk test/6MWT and gait speed). We will also examine satisfaction, attendance rates and adverse event (falls, soft tissue injuries, muscle soreness, etc) rates.

9.2 Sample Size

We base our sample size justification on prior data from our RESTORE and PRIME pilot studies, two-tailed tests conducted at the $\alpha=0.05$ level, a conservative attrition rate of 10% between baseline and follow-up assessments, practical consideration of a class size of 10 participants for group exercise, computational techniques that match our study design and proposed analytical approach as much as possible within the constraints of already published methodologies and commercially available sample size and power software (PASS 2002®, Number Cruncher Statistical Systems, Inc., Kayesville, Utah), and ability to detect differences that correspond to published meaningful change criteria(57) or moderate Cohen's effect sizes of $d=0.5$.(58)

The numbers needed to enroll in order to detect statistical significance of intervention effect when delivered by an exercise leader (Aim 1), are presented in **Table 1.** We take into account the clustering of participants by facility by assuming an intracluster correlation of 0.1 and a resulting design effect of 1.90 to appropriately inflate the sample size accordingly.(59) In

summary, a total of 280 participants with 140 in each arm will allow us to detect statistical significance in intervention effects in all main outcomes with at least 80% statistical power.

Table 1. Number needed to enroll for detecting effects of intervention incorporating clustered design (x1.90), attrition (10%), and rounded up to class size of 10.

Outcome	Prior Information			Estimated Sample Size	
	Baseline Standard Deviation (SD)	SD in Baseline-Follow up Change	Meaningful Difference Targeted (Source)	Completers Needed Per Arm assuming No Clustering	Number Needed to Enroll Per Arm
LLFDI Overall Function	6.15	4.86	3.08 (*d*=0.5)	41	90
LLFDI Disability Frequency	6.35	4.76	3.18 (*d*=0.5)	37	80
Gait Speed (m/s)	0.13	0.20	0.10[65]	64	140
6MWD (m)	62.0	42.8	50[65]	13	30

Our sustainability Aim 2 depends on whether we are able to train a staff activity person ready to lead a class safety and per protocol. Further, we do not have a priori data in sufficient detail that allows us to reliably estimate the likelihood of suitably training a staff activity person in each of the main type of facility. Thus Aim 2 is exploratory in nature and we will also focus on barriers to suitably training a staff activity person. We anticipate that we will enroll approximately 120 participants into Aim 2. The power available to detect a statistical significant difference in gains attributable to *On the Move* when delivered by exercise leaders and staff activity personnel (sustainability hypothesis – Aim 2) are in **Table 4**. Power is ≥90% for all outcomes except for gait speed when comparing On the Move to Standard when delivered by facility staff in which case power is 78%.

Table 4. Number needed to enroll for establishing sustainability of *On the Move*, incorporating clustered design (x0.90), attrition (10%), and rounded up to class size of 10.

Outcome	Prior Information			Estimated Sample Size		New Aim 2 Statistical Power with Anticipated N=140+140+60+60	
	Baseline Standard Deviation (SD)	SD in Baseline-Follow up Change	Meaningful Difference Targeted (Source)	Completers Needed Per Arm assuming No Clustering	Number Needed to Enroll Per Arm	Facility Staff as Good as Exercise Leader in Delivering OTM? N=140 vs 60	OTM More Effective than Standard Even when Delivered by Facility Staff? N=60 vs 60
LLFDI Overall Function	6.15	4.86	3.08 (*d*=0.5)	66	70	98%	93%
LLFDI Disability Frequency	6.35	4.76	3.18 (*d*=0.5)	60	60	99%	95%
Gait Speed (m/s)	0.13	0.20	0.10[65]	105	110	90%	78%
6MWD (m)	62.0	42.8	50[65]	21	30	>99%	>99%

9.3 **Data Analyses**

9.3.1 Overview

All statistical analyses will be performed or overseen by Dr. Perera using SAS® version 9 (SAS Institute, Inc., Cary, North Carolina) and Salford Predictive Miner® (Salford Systems, Inc., San Diego, California) based on the **intention-to-treat** philosophy. We will begin by summarizing data by arm and time point as well as pre- to post-intervention change using appropriate descriptive statistics for continuous (mean, standard deviation, median, range) and categorical (frequencies, percentages) to elicit information about general data quality and their distributional characteristics. Next, we will perform the modeling and inferential analyses to address the main hypotheses. First, the baseline participant characteristics will be compared between the two arms (see Avoidance of Bias below). Although no significant differences are expected, any significant differences will be noted and accounted for as covariates in the main analyses. Second, main analyses to address Aims 1-3 will be performed as outlined below. If residuals reveal violations of linear models assumptions, we will Box-Cox transform(60) the response variables. The two-step protected test approach will be used to control the experimentwise type I error rate from multiple outcomes, and multiple imputation(61, 62) will be used to account for any missing data in the main analysis. Third, we will perform the exploratory analyses to address Aim 4, using a data mining philosophy. Finally, we will perform a set of exploratory analyses to potentially extend our findings and generate new hypotheses, as well as a set of sensitivity analyses to assess the robustness of our findings.

9.3.2 Main Analysis for Aims 1and 3a

Aim1:

First, we will perform a multivariate Hotelling *t*-test to simultaneously compare the baseline to follow-up change in the three primary outcomes between the arms to protect the type I error rate from multiplicity. If significant, subsequent analyses will be performed without further multiplicity adjustment. If not, subsequent comparisons will be performed with a conservative Bonferroni correction at the $\alpha=0.05/4=0.0125$ level. This protected test approach has been recommended in the statistical literature(63) and used in other exercise intervention trials with multiple outcomes.(64)

Second, we will fit a series of linear mixed models(65) using the SAS® MIXED procedure with baseline to follow-up change in each of the continuous outcomes (LLFDI function/disability, walking ability, other measures of mobility performance) as the dependent variable; intervention arm (standard/*On the Move*), as the fixed effect of primary interest; baseline value of outcome as an additional fixed effect covariate; and a facility random effect to account for greater similarity of participants from the same facility compared to different facilities and resulting non-independence of observations within facility (ie. clustering). We will construct appropriate means contrasts to estimate difference in gains in the two interventions when delivered by exercise leaders (Aim 1) whose statistical significance of the estimates will serve as formal tests of hypotheses. We will consider adding other baseline measures found to be significantly

different between the arms or deemed important as additional fixed effect covariates in the model to assess robustness of the findings. We note that waiting of participants randomized to staff activity personnel classes constitute a group of control participants, if smaller, without any intervention. Therefore, we will perform another sensitivity analysis employing a similar analytic strategy but with three intervention arms (*On the Move*/Standard/Wait List Control).

Aim 3(a):

We will fit a series of generalized estimating equations (GEE) models(66) using the SAS® GENMOD procedure with each of the dichotomous adverse events, adherence (21+ sessions or ≥90%) and satisfaction outcomes as the dependent variable; a binomial distribution for the outcome and a logit canonical link function; intervention arm, as the effect of primary interest; baseline value of outcome and any other measures found to be different between arms or deemed important as additional fixed effects covariates; and an exchangeable working correlation structure to account for clustering due to facility. We will appropriately construct contrasts to test hypotheses of differential proportions with adverse events based on intervention when delivered by exercise leaders.

9.3.3 Aims 2, 3(b) and 4 exploratory Analyses

Aim 2:

We propose an analytic strategy with an exploratory philosophy for Aim 2 sustainability aim, because of the uncertainty surrounding our ability to recruit and train a staff activity person to lead a class safely and per protocol.

We will fit a series of linear mixed models[63] using the SAS® MIXED procedure with baseline to follow-up change in each of the continuous outcomes (LLFDI function/disability, walking ability, other measures of mobility performance) as the dependent variable; intervention arm (standard/*On the Move*), delivery mode (by exercise leader/staff activity personnel) and their interaction as fixed effects of interest; baseline value of outcome and any other measures found to be different between arms or deemed important as additional fixed effects covariates; and a facility random effect to account for greater similarity of participants from the same facility compared to different facilities and resulting non-independence of observations within facility (ie. clustering). We will construct appropriate means contrasts to estimate difference in gains in the two interventions when delivered by staff exercise personnel (Aim 2 effectiveness hypothesis); and difference in gains attributable to *On the Move* intervention when delivered by exercise leaders and staff activity personnel (Aim 2 sustainability hypothesis). Statistical significance of the estimates will serve as formal tests hypotheses. We note that participants randomized to staff activity personnel had to wait 12 weeks since their baseline assessment and randomization to start the intervention, during which they may have changed; and that they underwent a second baseline assessment immediately prior to starting the exercise intervention. Therefore, we will repeat the above analysis using their second baseline assessment instead of the first one to assess the sensitivity of results.

Aim 3(b):

will fit a series of generalized estimating equations (GEE) models[64] using the SAS® GENMOD procedure with each of the dichotomous adverse events, adherence (21+ sessions or ≥90%) and satisfaction outcomes as the dependent variable; a binomial distribution for the outcome and a logit canonical link function; intervention arm, delivery model and their interaction as effects of interest; baseline value of outcome and any other measures found to be different between arms or deemed important as additional fixed effects covariates; and an exchangeable working correlation structure to account for clustering due to facility. We will appropriately construct contrasts to test hypotheses of differential proportions with adverse events based on intervention when delivered by staff activity personnel and delivery mode.

Aim 4:
We will perform the exploratory analyses to identify combinations of baseline predictors of treatment response and risks of participating in *On the Move* program. We do not anticipate differences in outcomes of On the Move program based on delivery mode when a suitable staff person can be trained (Exploratory sustainability Aim 2), and thus propose to combine *On the Move* groups led by exercise leaders and staff activity personnel in the present analysis to maximize sample size and amount of information available for this analysis. In the unlikely event of differences due to delivery mode, we will stratify the Aim 4 analysis by delivery mode.

Adverse events and markers of response to treatment in walking ability have readily available dichotomous operational definitions based on simply presence/absence of an event or evidenced based criteria for having achieved meaningful change in gait speed (0.1+ m/s) and 6MWT (50m).(57) For such dichotomous markers of benefit and harm, we will fit a series of logistic regression models using the SAS® LOGISTIC procedure and classification tree models(67, 68) using Salford Predictive Miner® software with each measure of whether there was a benefit/harm from *On the Move* as the dichotomous response variable; and baseline physical, psychosocial and demographic characteristics as predictors. Logistic regression models are more efficient when associations are linear, and hard-to-discover higher order interactions and non-linearity and/or multicollinearities among predictors do not exist, while classification tree models are more efficient when they do exist. In terms of practically applying prediction criteria, results from logistic regression models require substituting values of predictors to a regression equation while classification tree model produces a flowchart that can be used as is in a clinical setting when a new individual is presented with known predictors. Thus we will use both methods to obtain areas under the receiver operator characteristic curve (AUROCC) to quantify predictive accuracy, and use the results from the method with a greater AUROCC. If the AUROCCs are not substantially different, we will favor the classification tree results due to ease of interpretation and utility in the clinical setting (see **Figure 2** for a hypothetical template for summarization of results). For logistic regression modeling, we will include all available baseline information simultaneously in the model as predictors, and use the backward elimination stepwise procedure to guard against model over-fitting to obtain a parsimonious model with a small number of most relevant predictors to facilitate interpretation, communication and clinical utility of the model. For the classification tree models, we will use all predictors available and use a minimum misclassification-complexity cost tree to achieve the same objectives. We will use the method of DeLong(69) to obtain the statistical significance of the improvement in predictive accuracy, as measured by AUROCC.

Our LLFDI function and disability outcomes do not have the same level of evidence based for creating intuitively appealing dichotomous measures of treatment response. Thus we will consider pre- to post-intervention change as a continuous variable, and perform analogous analysis to that for dichotomous outcomes but with appropriate statistical models. Specifically, we will fit standard multiple linear regression models using the SAS® REG procedure and regression tree models(67, 68) using Salford Predictive Miner®; and use proportion of explained variance (R^2) to quantify predictive accuracy.

9.3.4 Additional Exploratory/Sensitivity and Compliance/Dropout Analyses

We will perform additional analyses to extend our findings, generate new hypotheses, assess robustness and potentially refine conclusions. They will include using an alternative threshold besides 90% to operationally define high level of adherence/compliance calculating proportion missing each session to describe the pattern of adherence/compliance over time; assessing intervention effects using as-treated instead of intention-to-treat philosophy; and alternative operational definitions of treatment response such as combinations of walking ability, function and/or disability, and reaching a published threshold such as 0.4(0.8) m/s in gait speed for limited(full) community ambulation.(70)

Figure 2. Hypothetical template for summarization of Aim 4 results.

Figure 1. Predictors of treatment response using age, gait speed, and fear of falling. Area under ROC curve=0.797

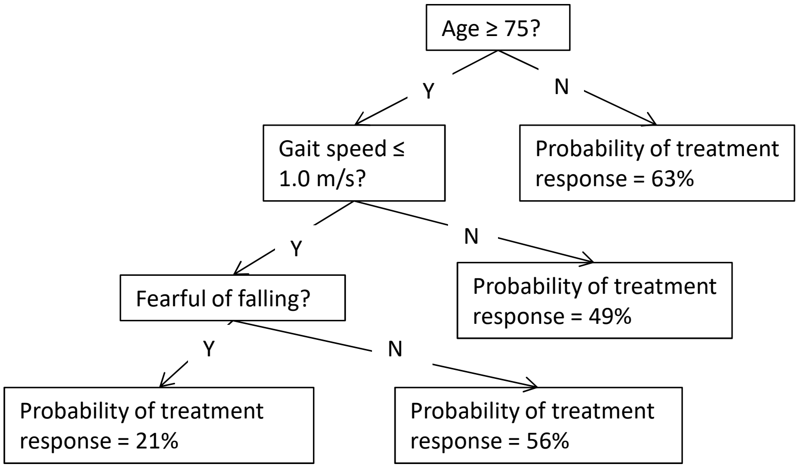

10 DATA COLLECTION AND QUALITY ASSURANCE

10.1 Data Collection Forms

Data collection will consist of paper forms. Data collected on paper forms will be entered into the electronic database by the research staff.

Screening and baseline data collection, which will occur prior to randomization, will be conducted by research staff trained in the outcomes and may include the study coordinator if necessary. Outcome assessments post-intervention will only be conducted by research staff trained in the outcomes and who are blinded to the intervention group assignment.

Participants’ confidentiality will be protected in the data collection process. All study personnel are certified in Research Practice Fundamentals, Human Subjects Research Module. Consent forms and paper data collection forms will be stored in locked file cabinets. All computers are password protected. Only authorized team members will have access to personal information needed for tracking and informed consent.

10.2 **Data Management**

An electronic tracking system will monitor enrollment, track follow-up rates and the data entry process, providing up-to-date status reports. All completed data collection forms will be entered into a secure relational database located in a local network, and stored in a secure location. To improve accuracy, the data entry screens are identical in appearance to the paper forms. The data entry system includes automatic and routine data quality checks for out-of-range and extreme values, and automatic enforcement of skip patterns. In addition, functionality will be built in to the data entry system to facilitate double data entry and comparison of two versions so that discrepancies can be resolved against the authoritative paper forms. All screened subjects will be assigned unique subject identifiers that will appear on all data collection forms and files and serve as an index in database tables. The database will have access restricted to only those study personnel who need it and the level of access (read/write) will depend on the specific role. All files will be backed-up daily and archived weekly, including storage of back-up copies in an off-site location.

10.3 **Quality Assurance**

10.3.1 Intervention

Program monitoring: Periodically throughout the exercise program we will be videotaping the exercise sessions. These videos will be used primarily to monitor the quality and consistency of the exercise program and to further develop the training manual for the exercise instructors. The videos may be used to train future exercise instructors.

At each session, the exercise leaders and activity staff personnel will complete the exercise class log (see 9.3 above). Dr. VanSwearingen will review the exercise logs monthly to make sure the interventionists are following the protocol and progressing the exercise class appropriately. If deficiencies are notes, we will review the program with the interventionist and discuss potential modifications to the administration of the program.

10.3.2 Protocol Deviation Tracking

Protocol deviations may occur in the randomization process, exercise intervention protocols, the timing or completion of testing sessions, and in the completion of data forms. A Protocol Deviation Form has been developed for the study and will be completed, dated, and signed for each protocol deviation that may occur for each subject. This form will be kept in a folder for

protocol deviations. The protocol deviation will also be noted in the progress section of the subject's research record.

10.3.3 Subject Termination

If subject participation in the study is terminated for any reason (death, self-withdrawal, lost-to-follow-up, or change in health) a Study Termination form will be completed by the trial coordinator and placed in the subject's research record.

11 PARTICIPANT RIGHTS AND CONFIDENTIALITY

11.1 Institutional Review Board (IRB) Review

The study protocol, the informed consent document and any subsequent modifications will be reviewed and approved by the University of Pittsburgh IRB.

11.2 Informed Consent Forms

All potential participants will be adults (65 years of age or older) who are capable of providing direct consent for their participation in the study. Written informed consent will be obtained at the clinic screening visit prior to performing any of the clinic screening procedures. One of the study investigators will explain the study and the participant will be given a copy of the consent form to read. The consent form will describe the purpose of the study, the procedures to be followed, and the risks and benefits of participation. The investigator will answer any questions that the participant may have about the study. Finally, the participant will be asked to sign and date the consent form. The participant will be given a copy of the consent form for their records.

11.3 Participant Confidentiality

Research assessments and interventions are conducted in the community/activities room. This space includes several small tables in different portions of the room that can be used to privately conduct questionnaires and simple physical examination measures. Many of the walking assessments are done in the open area. Participants may be screened individually, which further protects their privacy. Participants are informed via the consent process that the treatment programs are conducted in groups. Although not completely private, this level of exposure to others during exercise is similar to what one might experience at a physical therapy appointment or during an exercise class at a public gym.

Participant's confidentiality will be protected in the data collection process. All personnel involved with the research have read and signed a Confidentiality statement. All study related data will be maintained in secure locked hard copy files and password protected computer files. To facilitate referring to our older adult subjects by name throughout all testing sessions and to minimize errors that could occur while multiple testers collect data on multiple participants in the same research space at the same time, data sets will not be de-identified. Subjects' names and emergency contact information will be maintained in both the hard copy and computer files, and subjects will be made aware of this during the informed consent process.

If data are used in scholarly presentations or journal articles, the investigators will protect the anonymity of individual participants and will report only aggregate data (eg group means) where appropriate. The Principal Investigator will review data confidentiality process monthly or as indicated with the research staff. The Investigators are all certified in Research Practice Fundamentals, Human Subjects Research Module.

11.4 Study Discontinuation

The study may be discontinued at any time by the IRB, the NIA, the OHRP, or other government agencies as part of their duties to ensure that research participants are protected.

12 STUDY TIMELINE

In this three year project we will conduct a single-blind cluster randomized intervention trial. The trial will be conducted in at least 28 facilities (ILFs and Senior High Rises) and will include 280 community-dwelling older adults for Aim 1 and up to 280 community-dwelling older adults for Aim 2. We will attempt to train 28 staff activity personnel and will conduct 56, 12 week group exercise sessions. The table below contains the timeline for all research activities.

Table - Project Timeline.

Research Activity	Year 1				Year 2				Year 3			
Hire and train research staff	X	X										
Develop manual of operations	X	X										
Assemble Advisory Board	X											
Train staff activity personnel			X	X	X	X	X	X				
Finalize data collection forms	X	X										
Construct database		X	X									
Recruitment			X	X	X	X	X	X	X	X		
Baseline testing			X	X	X	X	X	X	X	X		
Conduct exercise programs				X	X	X	X	X	X	X		
Post testing				X	X	X	X	X	X	X	X	
In-depth satisfaction interviews				X	X	X	X	X	X	X		
In-depth interviews of Community Advisory Board					X	X						
Data entry				X	X	X	X	X	X	X	X	
Analysis										X	X	X
Review and interpret results										X	X	X
Dissemination												X

Meetings												
Advisory Board meetings	X		X		X		X		X		X	
Research staff	X	X	X	X	X	X	X	X	X	X	X	X
Data Safety Monitoring Meeting		X				X				X		

Year 1

In the first 6 months of funding, we will hire and train research staff, develop the manual of operations, assemble to Advisory Board, finalize the data collection forms, and construct the database. In the second 6 months of Year 1 we will initiate recruitment and baseline testing of research subjects. The group exercise programs will begin in a minimum of 4 facilities in months 10-12 of Year 1. The deliverables for Year 1 are outlined in the milestone schedule.

Year 2

At the beginning of Year 2 we will conduct the in-depth interviews of the Community Advisory Board members. The majority of the group exercises classes will be conducted in Year 2. We will continue to train the staff activity personnel (months 1-9 Year 2) as we include additional facilities in the research study. Our goal is that every quarter we would introduce the exercise program to 6 facilities and train the staff activity personnel to conduct the exercise program. In Year 2 we will recruit subjects to participate, baseline test the subjects, conduct the 12 week exercise program, and conduct the post testing. By the end of year 2, we will have trained 24 staff activity personnel to conduct the exercise program and completed forty-eight 12 week exercise sessions within 24 facilities. The deliverables for Year 2 are outlined in the milestone schedule.

Year 3

In Year three we will complete subject recruitment and the exercise group sessions and complete all post testing. The second six months of Year 3 will be dedicated to data entry, data analysis, review and interpretation of results and dissemination of research findings. See the milestone schedule for Year 3 deliverables.

13 PUBLICATION OF RESEARCH FINDINGS

Publications will be operationally defined as manuscripts for publications; abstracts for platform or poster presentation at scientific meetings and other professional meetings; slides for presentation at scientific and other meetings; doctoral dissertations; and master's theses.

The goal of the publication policy is to encourage and facilitate publication of study results. The purposes of this policy are to ensure the following:

- On the Move publications will be of the highest scientific quality
- On the Move will be described in a consistent manner across publications
- Measures are reported in consistent ways across publications
- Proper acknowledgements are included
- Appropriate authorship credit is determined prior to submission of manuscripts for publication consideration.

Publications from On the Move will be overseen by the PI and Co-Investigators.

14 REFERENCES

1. Guralnik J, Ferrucci L, Simonsick E, Salive M, Wallace R. Lower extremity function in persons over the age of 70 years as a predictor of subsequent disability. New Engl J Med. 1995;332:556-61.
2. Cesari M, Kritchevsky S, Bauer D, Visser M, Rubin S, Harris T, et al. Prognostic value of usual gait speed in well-functioning older people--results from the Health, Aging and Body Composition Study. J Am Geriatr Soc. 2005;53:1675-80.
3. Guralnik J, Ferrucci L, Pieper C, Leveille S, Markides K, Ostir G, et al. Lower extremity function and subsequent disability: consistency across studies, predictive models, and value of gait speed alone compared with the short physical performance battery. J Gerontol Med Sci. 2000;55A:M221-M31.
4. Guralnik J, Simonsick E, Ferrucci L, Glynn R, Berkman L, Blazer D, et al. A short physical performance battery assessing lower extremity function: Association with self-reported disability and prediction of mortality and nursing home admission. J Gerontol. 1994;49:M85-M94.
5. Studenski S, Perera S, Patel K, Rosano C, Faulkner K, Inzitari M, et al. Gait speed and survival in older adults. JAMA. 2011;305(1):50-8.
6. Fried L, Bandeen-Roche K, Chaves P, Johnson B. Preclinical Mobility Disability Predicts Incident Mobility Disability in Older Women. Journal of Gerontology. 2000;55A(1):M43-M52.
7. Hoffman J, Ciol M, Huynh M, Chan L. Estimating transitions probabilities in mobility and total costs for Medicare beneficiaries. Arch Phys Med Rehabil. 2010;91:1849-55.
8. Guralnik J, Ferrucci L, Balfour J, Volpato S, Di I, A. Progressive versus catastrophic loss of the abilit to walk: Implications for the prevention of mobility loss. J Am Geriatr Soc. 2001;49:1463-70.
9. Brach J, Studenski S, Perera S, VanSwearingen J, Newman A. Gait variability and the risk of incident mobility disability. J Gerontol Med Sci. 2007;62A:983-8.
10. Hausdorff J, Rios D, Edelberg H. Gait variability and fall risk in community-living older adults: a 1-year prospective study. Arch Phys Med Rehabil. 2001;82:1050-6.
11. Judge J, Underwood M, Gennosa T. Exercise to improve gait velocity in older adults. Arch Phys Med Rehabil. 1993;74:400-6.
12. Mian O, Thom J, Ardigo L, Morse C, Narici M, Minetti A. Effect of a 12-month physical conditioning programme on the metabolic cost of walking in healthy older adults. Eur J Appl Physiol. 2007;100:499-505.
13. Brown M, Holloszy J. Effects of a low intensity exercise program on selected physical performance characteristics of 60- to 71-year olds. Aging (Milano). 1991;3:129-39.

14. Manini T, Marko M, VanArnam T, Cook S, Fernhall B, Burke J, et al. Efficacy of resistance and task-specific exercise in older adults who modify tasks of everyday life. J Gerontol Med Sci. 2007;62A:616-23.
15. Wolf S, O'Grady M, Easley K, Guo Y, Kressig R, Kutner M**.** The influence of intense Tai Chi training on physical performance and hemodynamic outcomes in transitionally frail, older adults. J Gerontol Med Sci. 2006;61A:184-9.
16. Helbostad J, Sletvold O, Moe-Nilssen R**.** Home training with and without additional group training in physically frail older people living at home: effect on health-related quality of life and ambulation. Clinical Rehabilitation. 2004;18:498-508.
17. Buchner D, Cress M, de L, BJ, Esselman P, Margherita A, Price R, et al. A comparison of the effects of three types of endurance training on balance and other fall risk factors in older adults. Aging Clin Exp Res. 1997;9:112-9.
18. Buchner D, Cress M, de L, BJ, Esselman P, Margherita A, Price R, et al. The effects of strength and endurance training on gait, balance, fall risk, and health services use in community-living older adults. Journal of Gerontology: Medical Sciences. 1997;52A(4):M218-M24.
19. Bean J, Herman S, Kiely D, Frey I, Leveille S, Fielding R, et al. Increased velocity exercise specific to task training: a pilot study exploring effects on leg power, balance, and mobility in community dwelling older women. J Am Geriatr Soc. 2004;52(5):799-804.
20. LIFE S, Investigators**.** Effects of a physical activity intervention on measures of physical performance: results of the Lifestyle Interventions and independence for elders pilot (LIFE-P) study. J Gerontol Med Sci. 2006;61A:1157-65.
21. Liu C, Latham N**.** Progressive resistance strength training for improving physical function in older adults. Cochrane Database of Systematic Reviews. 2009(3).
22. Nelson M, Rejeski W, Blair S, Duncan P, Judge J, King A, et al. Physical activity and public health in older adults: recommendation from the American College of Sports Medicine and the American Heart Association. Med Sci Sports. 2007;39(8):1435-45.
23. VanSwearingen J, Perera S, Brach J, Cham R, Rosano C, Studenski S**.** A randomized trial of two forms of therapeutic activity to improve walking: effect on the energy cost of walking. J Gerontol A Biol Sci Med Sc. 2009;64A:1190-8.
24. Nelson W**.** Physical principles for economies of skilled movements. Biol Cybernetics. 1983;46:135-47.
25. Daly J, Ruff R**.** Construction of efficacious gait and upper limb functional interventions based on brain plasticity evidence and model-based measures for stroke patients. The Scientific World Journal. 2007;7:2031-45.
26. Lay B, Sparrow W, Hughes K, O'Dwyer N**.** Practice effects on coordination and control, metabolic energy expenditure, and muscle activation. Human Movement Science. 2002;21:807-30.
27. Newman M, Dawes H, van d, Berg, M, Wade D, Burridge J, Izadi H**.** Can aerobic treadmill training reduce the effort of walking and fatigue in people with multiple sclerosis: a pilot study. Multiple Sclerosis. 2007;13:113-9.
28. Brooks V**.** The Neural Basis of Motor Control. New York: Oxford University Press; 1986.

29. Gentile A. Skill acquisition: action, movement, and neuromotor processes. In: JH C, RB S, J G, AM G, JM H, eds. Movement Sciences. 1 ed. Rockville: Aspen Publishers; 1987:93-154.
30. Polcyn A, Lipsitz L, Kerrigan C, Collins J. Age-related changes in the initiation of gait: degradation of central mechanisms for momentum generation. Arch Phys Med Rehabil. 1998;79:1582-9.
31. Capaday C. The special nature of human walking and its neural control. Trends in Neurosciences. 2002;25(7):370-6.
32. Alexander R. Walking made simple. Science. 2005;308:58-9.
33. Schmidt R. Organizing and Scheduling Prcatice. In: RA S, ed. Motor Learning and Practice: From Principles to Practice. Champaign, IL: Human Kinetics Books; 1991:199-225.
34. ACSM's Guidelines for Exercise Testing and Prescription. 5th ed. Baltimore, MD: Williams & Wilkins; 1995.
35. Haley S, Jette A, Coster W, Kooyoomijian J, Levenson S, Heeren T, et al. Late life function and disability instrument:II. Development and evaluation of the function component. J Gerontol. 2002;57A:M217-M22.
36. Jette A, Haley S, Coster W, Kooyoomijian J, Levenson S, Heeren T, et al. Late life function and disability instrument: I. Development and evaluation of the disability component. J Gerontol. 2002;57A:M209-M16.
37. Butland R, Pang J, Gross E, Woodcock A, Geddes D. Two-, six-, and 12-minute walking tests in reespiratory disease. BMJ. 1982;284:1607-8.
38. Lerner-Frankiel M, Vargas S, Brown M, Krusel L, Schoneberger W. Functional community ambulation: what are your criteria? Clinincal Management. 1986;6((2)):12-5.
39. Robinett C, Vondran M. Functional ambulation velocity and distance requirements in rural and urban communities. Phys Ther. 1988;68(9):1371-3.
40. Solway S, Brooks D, Lacasse Y, Tomas S. A qualitative, systematic overview of the measurement properties of the functional walk tests used in the cardioresepiratory domain. Chest. 2001;119:256-70.
41. Harada N, Chiu V, Damron-Rodriguez J, Fowler E, Siu A, Reuben D. Screening for balance and mobility impairment in elderly individuals living in residential care facilities. Physical Therapy. 1995;75(6):462-9.
42. Harada N, Chiu V, Stewart A. Mobility-related function in older adults: assessment with a 6-minute walk test. Arch Phys Med Rehabil. 1999;80:837-41.
43. Guyatt G, Sullivan M, Thompson P. The 6-minute walk: a new measure of exercise capacity in patients with chronic heart failure. Can Med Assoc. 1985;132:919-23.
44. Brach J, Perera S, Studenski S, Newman A. Reliability and validity of measures of gait variability in community-dwelling older adults. Arch Phys Med Rehabil. 2008;89:2293-6.
45. McAuley E, Mihalko S, Rosengren K. Self-efficacy and balance correlates of fear of falling in the elderly. J Aging Phys Activity. 1997;5:329-40.
46. Rosengren K, McAuley E, Mihalko S. Gait adjustments in older adults: Activity and efficacy influences. Psychology and Aging. 1998;13:375-80.
47. Newell A, VanSwearingen J, Hile E, Brach J. The modified gait efficacy scale: establishing the psychometric properties in older adults. Phys Ther. 2012;92:318-28.
48. Hess R, Brach J, Piva S, VanSwearingen J. Walking skill can be assessed in older adults: Validity of figure-of-8 walk test. Phys Ther. 2010;90:89-99.

49. Brach J, Perera S, VanSwearingen J, Hile E, Wert D, Studenski S. Challenging gait conditions predict 1-year decline in gait speed in older adults with apparently normal gait. Phys Ther. 2011;91:1857-64.
50. Gabell A, Nayak U. The effect of age and variability in gait. Journal of Gerontology. 1984;39(6):662-6.
51. Perera S, Brach J, Talkowski J, Wert d, Studenski S. Measuring stride time variability: estimating test-retest reliability and required walk length using bootstrapping. Program & Abstracts of the ISPGR 18th International Conference. 2007:55-6.
52. Brach J, Berlin J, VanSwearingen J, Newman A, Studenski S. Too much or too little step width variability is associated with a fall history in older persons who walk at or near normal gait speed. J Neuroengineering Rehabil. 2005;2(21).
53. Madden DJ, Whiting WL, Cabeza R, Huettel SA. Age-related preservation of top-down attentional guidance during visual search. Psychol Aging. 2004;19(2):304-9.
54. Kroenke K, Spitzer RL, Williams JB. The PHQ-9: validity of a brief depression severity measure. J Gen Intern Med. 2001;16(9):606-13.
55. Gardner M, Robertson C, Campbell A. Exercise in preventing falls and fall related injuries in older people: a review of randomized controlled trials. Br J Sports Med. 2000;34:7-17.
56. Meinert C. Clinical Trials. New York: Oxford University Press; 1986.
57. Perera S, Mody S, Woodman R, Studenski S. Meaningful change and responsiveness in common physical performance measures in older adults. J Am Geriatr Soc. 2006;54:743-9.
58. Cohen J. Satistical Power Analysis for the Behavioral Sciences. New York: Academic Press; 1977.
59. Eldridge S, Ashby D, Kerry S. Sample size for cluster randomized trials: effects of coefficient of variation of cluster size and analysis method. International Journal of Epidemiology. 2006;35:1292-300.
60. Box G, Cox D. An analysis of transformations. Journal of the Royal Statistical Society-Series B. 1964;26:211-43.
61. Rubin D. Multiple Imputation for Nonresponse in Surveys: John Wiley and Sons; 1987.
62. Rubin D. Multiple imputation after 18+ years. Statistics in Medicine. 1991;14:1913-25.
63. Johnson D. Applied Multivariate Statistics. Belmont, CA: Duxbury Press; 1998.
64. Duncan P, Studenski S, Richards L, Gollub S, Lai S, Reker D, et al. Randomized clinical trial of therapeutic exercise in subacute stroke. Stroke. 2003;34(9):2173-80.
65. Milliken G, Johnoson D. Analysis of Messy Data Volume 1: Designed Experiments. New York: Van Nostrand Reinhold; 1992.
66. Diggle P, Liang K, Zeger S. Analysis of Longitudinal Data. Oxford: Clarendon Press; 1994.
67. Breiman L, Friedman J, Stone C, Olshen R. Classification and Regression Trees: CRC Press; 1984.
68. Strobl C, Malley J, Tutz G. An introduction to recursive partioning: rationale, application, and characteristics of classification and regression trees, bagging, and random forests. Psychological Methods. 2009;14(4):323-48.
69. DeLong E, DeLong D, Clarke-Pearson D. Comparing the areas under two or more correlated receiver operating characteristic curves: a nonparametric approach. Biometrics. 1988;44:837-45.

70. Schmid A, Duncan P, Studenski S, Lai S, Richards L, Perera S, et al. Improvements in speed-based gait classifications are meaningful. Stroke. 2007;38(7):2096-100.
