# Supplementary material for: Effectiveness of a Timing and Coordination Group Exercise Program to Improve Mobility in Community-Dwelling Older Adults: A Randomized Clinical Trial
Source: JAMA Intern Med. 2017 Oct 2;177(10):1437–44. doi: 10.1001/jamainternmed.2017.3609 (PMC5710210; doi:10.1001/jamainternmed.2017.3609)
Supplement: Supplement 2. — eMethods. Detailed description of the On the Move and Usual Care interventions eFigure 1a. Detailed CONSORT diagram part 1 eFigure 1b. Detailed CONSORT diagram part 2 [file jamainternmed-177-1437-s002.pdf]

Supplementary Online Content

Brach JS, Perera S, Gilmore S, et al. Effectiveness of a timing and coordination group exercise program to improve mobility in community-dwelling older adults: a randomized clinical trial. *JAMA Intern Med*. Published online August 14, 2017. doi:10.1001/jamainternmed.2017.3609

eMethods. Detailed description of the On the Move and Usual Care interventions
eFigure 1a. Detailed CONSORT diagram part 1
eFigure 1b. Detailed CONSORT diagram part 2

This supplementary material has been provided by the authors to give readers additional information about their work.

© 2017 Brach JS et al. *JAMA Internal Medicine.*

eMethods. Detailed description of the On the Move and Usual Care interventions

Section 1: General Concept and Structure of the On the Move Program

1.1 Overview
The On the Move (OTM) program is group-based motor learning exercise program designed to improve walking. It specifically challenges the nervous system through the use of stepping and walking patterns. In addition to stepping and walking patterns, the OTM program includes warm-up, cool-down, and strengthening components that target the musculoskeletal system. A more detailed description of each section of the program is included in section 1.3.

1.2 Space and Equipment Necessary
The OTM program is designed so that it can be administered in a variety of settings. It has been successfully administered in large fitness suites as well as smaller activities rooms. General criteria for the space required to administer the OTM program are as follows:

1) Minimum dimensions: Approximately 1200 square feet or 30 feet x 40 feet
2) Level surface clear of obstacles other than those being used for the program

In order to appropriately implement the program, the following equipment is required:

1) CD player with remote
2) On The Move CD collection
3) 11 multicolored playground balls
4) 10 multicolored cones
5) 11 sturdy chairs

1.3 Class Structure and Rationale
The OTM program consists of 5 component parts: warm-up, walking patterns, stepping, strengthening, and cool-down. Specific music playlists have been designed to be used during OTM classes. When teaching the class, it is recommended that the instructor use the remote to pause the music when providing verbal instruction. The following provides a short description and rationale for each specific section.

a. Warm-up, *less than 10 minutes (2-3 songs).* This section consists of basic weightshifting and stepping exercises to prepare the musculoskeletal and nervous systems for what is to come in the remainder of the class. Additionally, the simple movements in this section promote an appropriate cardiopulmonary response to exercise. All exercises in this section are completed in a supported or unsupported standing position.
b. Walking patterns, *approximately 10-20 minutes (3-6 songs).* This section consists of walking in a variety of pre-determined patterns. The walking patterns are designed to promote the appropriate timing and coordination of stepping during walking by enhancing proper weightshift during stepping and appropriate coordination of the legs and trunk during walking. Some of the more advanced walking patterns use a playground ball in the participants hand as a facilitative device for promoting

© 2017 Brach JS et al. *JAMA Internal Medicine.*

appropriate weight shift. Cones are used to create the various walking patterns in the exercise space.

c. Stepping, *approximately 10-20 minutes (2-4 songs).* This section consists of an extensive progression of stepping sequences. Like the walking patterns, the stepping sequences are designed to promote the appropriate timing and coordination of stepping during walking by enhancing proper weightshift during stepping and appropriate coordination of the legs and trunk during walking. All stepping patterns can be completed with or without upper extremity support. Some stepping patterns are completed with a partner and a playground ball or cone.

d. Strengthening, *approximately 10 minutes (2-3 songs).* This section consists of a series of lower extremity exercises that aim to increase muscular strength. It specifically targets the strength of lower extremity muscles that are important for walking (ex: hip abductors, ankle dorsiflexors, etc). Some of these exercises are completed in a supported standing position while others are completed sitting in a chair. A number of exercises in this section require the use of a playground ball.

e. Cool-down, *less than 5 minutes (1-2 songs).* This section consists of a brief set of lower extremity stretches that provides the body with the cues it needs to return to the resting state. During this time, the heart rate and blood pressure should return to a level that is at or close to each participant's pre-exercise level.

Section 2: On the Move Program Materials

2.1 Warm-up

1. Weightshifting: Rocking side to side
   Stand with feet shoulder width apart. Gently shift you weight onto one foot, really getting the weight onto that foot. If you feel comfortable, as you shift onto that foot, the heel on the other foot could come slightly off the ground. Then shift the weight onto the other foot. Again, if you feel comfortable, the heel of the foot without the weight on it could come up slightly off the ground.

   Variations/Progressions:
   - With one or both hands on chair
   - With no hands on chair
   - With arm movement

2. Weightshifting: Rocking back and forth with R foot in front
   Stand with feet in a comfortable position. Take a step forward with your right foot. Keeping your feet in place, shift your weight onto your front foot. If you feel comfortable, the heel of your back foot should come up slightly off the ground. Then shift your weight onto your back foot. If you feel comfortable, the toes on your front foot should come up slightly off the ground.

   Variations/Progressions:
   - With hand on chair
   - With no hands on chair
   - With arm movements

3. Weightshifting: Rocking back and forth with L foot in front
   Stand with feet in a comfortable position. Take a step forward with your left foot. Keeping your feet in place, shift your weight onto your front foot. If you feel comfortable, the heel of your back foot should come up slightly off the ground. Then shift your weight onto your back foot. If you feel comfortable, the toes on your front foot should come up slightly off the ground.

   Variations/Progressions:
   - With one hand on chair
   - With no hands on chair
   - With arm movements

4. Stepping: Forward/forward, back/back with R foot leading
   Stand with feet in a comfortable position. Take a step forward with the right foot, then bring the left foot forward to meet the right. Take a step backward with the right foot, then bring the left foot backward to meet the right. Continue this pattern, always starting with the right foot and following with the left foot.

   Variations/Progressions:
   - With one hand on chair
   - With no hands on chair

5. Stepping: Forward/forward, back/back with L foot leading
   Stand with feet in a comfortable position. Take a step forward with the left foot, then bring the right foot forward to meet the left. Take a step backward with the left foot, then bring the right foot backward to meet the left. Continue this pattern, always starting with the left foot and following with the right foot.

   Variations/Progressions:
   - With one hand on chair
   - With no hands on chair

6. Stepping: Square step with R foot leading
   (R forward/L forward, R side/L side, R back/L back, L side/R side)
   Stand with feet in a comfortable position. Take a step forward with the right foot, then bring the left foot forward to meet the right. Take a step to the side with the right foot, then take a step to the same side with the left foot. Take a step backward with the right foot, then bring the left foot backward to meet the right. Take a step to the side with the left foot, then take a step to the same side with the right foot. You should end up in the starting position. **It is possible to do this exercise with hands on a chair, but it is easier to complete without holding on.**

   Variations/Progressions:
   - With one hand on chair
   - With no hands on chair

© 2017 Brach JS et al. *JAMA Internal Medicine.*

7. Stepping: Square step with L foot leading
   (L forward/R forward, L side/R side, L back/R back, R side/L side)
   Stand with feet in a comfortable position. Take a step forward with the left foot, then bring the right foot forward to meet the left. Take a step to the side with the left foot, then take a step to the same side with the right foot. Take a step backward with the left foot, then bring the right foot backward to meet the left. Take a step to the side with the right foot, then take a step to the same side with the left foot. You should end up in the starting position. **It is possible to do this exercise with hands on a chair, but it is easier to complete without holding on.**

   Variations/Progressions:
   - With one hand on chair
   - With no hands on chair

8. Stepping: Out/out, in/in with R foot leading
   Stand with feet close together. Take a moderate step out to the right with the right foot. Take a moderate step out to the left with the left foot. Take a moderate step in (to the left) with the right foot. Take a moderate step in (to the right) with the left foot. **When taking the first step out, do not step out too far. It will make the following steps too difficult.**

   Variations/Progressions:
   - With hand(s) on chair
   - With no hands on chair

9. Stepping: Out/out, in/in with L foot leading
   Stand with feet close together. Take a moderate step out to the left with the left foot. Take a moderate step out to the right with the right foot. Take a moderate step in (to the right) with the left foot. Take a moderate step in (to the left) with the right foot. **When taking the first step out, do not step out too far. It will make the following steps too difficult.**

   Variations/Progressions:
   - With hand(s) on chair
   - With no hands on chair

2.2 Walking Patterns

1. Simple ovals
   Start by placing two cones a few feet apart at one end of the space and two cones a few feet apart at the other end of the space. Begin by walking clockwise around the outside of all four cones. At some point, turn around and walk counterclockwise around all four cones.

   Variations/Progressions:

- Wide oval
- Narrower oval (move cones at each end of space closer together)
- Single cone oval (use one cone at each end of space and walk around the two cones)
- With 10 second speed changes
- While passing ball from hand to hand

2. Concentric ovals
   Start by positioning cones in space so that there is a small oval inside of a larger oval. Split the class into two equal groups and have one group walk clockwise around the larger oval and the other group walk clockwise around the smaller oval. At some point, have each group turn around and walk counterclockwise around their oval.

   Variations/Progressions:
   - With one group walking clockwise and the other group walking counterclockwise
   - With 10 second speed changes
   - While passing ball from hand to hand

3. Adjacent ovals
   Start by positioning cones in space so that there are two ovals of the same size next to one another. Split the class into two equal groups and have one group walk clockwise around one oval and the other group walk clockwise around the other oval. At some point, have each group turn around and walk counterclockwise.

   Variations/Progressions:
   - With one group walking clockwise and the other group walking counterclockwise
   - With 10 second speed changes
   - While is passing ball from hand to hand

4. Ovals of varying sizes (Spirals)
   Start by positioning two cones next to each other at one end of the space. This will serve as the "home base" for this exercise. Place three more cones in a row, with a few feet in between each of them. If the cones are not different colors, place a different color ball or number on each of the cones. Starting at the "home base," have each person walk around the third cone and back to "home base," then the middle cone and back to "home base", then the closest cone and back to "home base." You can then have the participant turn around and repeat the pattern while walking in the opposite direction. Once the first participant has completed it, the next participant can begin.

   Variations/Progressions:
   - Smallest oval to largest oval
   - Participants follow each other through task; change leader after each rep

© 2017 Brach JS et al. *JAMA Internal Medicine.*

- Random series of ovals (ex: around first cone then around third cone)
- Increase number of cones in task
- While passing ball from hand to hand

5. Serpentine with three cones with straight-away on return
   Start by setting up 3 cones in a row. Have participants start at one end of the cones. Instruct participants to walk to the left of the first cone, to the right of the second cone, and to the left of the third cone. Then have the participants walk straight back to the start. After completing this pattern several times, instruct them to repeat while starting on the right of the first cone.

   Variations/Progressions:
   - Increase the number of cones in task (use odd number only)
   - While passing ball from hand to hand

6. Serpentine with four cones with straight-away on return
   Start by setting up 4 cones in a row. Have participants start at one end of the cones. Instruct participants to walk to the right of the first cone, to the left of the second cone, to the right of the third cone, and to the left of the fourth cone. Then have the participants walk straight back to the start. On the next cycle, the participants will start on the left side of the first cone.

   Variations/Progressions:
   - Increase the number of cones in task (use even number only)
   - While passing ball from hand to hand

8. Nine Cone Sequence: Large Loop
   Start by setting up three rows of three cones such that each row is a different color. Begin by walking around the outer edge of all nine cones in the clockwise direction. At some point, turn around and walk around the outer edge of all nine cones in the counterclockwise direction.

   Variations/Progressions:
   - Complete in conjunction with any other Nine Cone Sequence walking pattern
   - While passing ball from hand to hand

9. Nine Cone Sequence: Overlapping Loops
   Start by setting up three rows of cones such that each row is a different color (ex: red, blue, yellow). Divide the class into two groups. Instruct one group to walk clockwise around two rows of cones (ex: red, blue) while the other group walks clockwise around two rows of cones (ex: blue, yellow) as well. One row should be in common between the two groups. At some point, have all participants turn around and complete the same pattern while walking counterclockwise.

   Variations/Progressions:

© 2017 Brach JS et al. *JAMA Internal Medicine.*

- Complete in conjunction with any other Nine Cone Sequence walking pattern
- While passing ball from hand to hand

10. Nine Cone Sequence: Diagonals
    Start by setting up three cones such that each row is a different color (ex: red, blue, yellow). Divide the class into two groups. Instruct one group to walk clockwise around one diagonal (which will include one cone of each color) while the other group walks clockwise around the opposite diagonal (which will include one cone of each color). The two groups will essentially be making an "X" with their walking pattern. At some point, have all participants turn around and complete the same pattern while walking counterclockwise around the diagonals.

    Variations/Progressions:
    - Complete in conjunction with any other Nine Cone Sequence walking pattern
    - Complete one diagonal followed by one outer edge straightaway then repeat
    - While passing ball from hand to hand

2.3 Stepping

1. Step straight ahead and return leading with R foot
   Stand with feet about shoulder width apart. Take a comfortable step straight ahead with your right foot, putting the heel down first and rolling onto the toe. Shift your weight onto the right foot, causing the heel of the left foot to come off the ground. Shift your weight back onto the left foot, and take a step back with the right foot so that your feet are back in the starting position. Repeat, taking steps with the right foot only.

   Variations/Progressions:
   - With one hand on chair
   - With no hands on chair

2. Step straight ahead and return leading with L foot
   Stand with feet about shoulder width apart. Take a comfortable step straight ahead with your left foot, putting the heel down first and rolling onto the toe. Shift your weight onto the left foot, causing the heel of the right foot to come off the ground. Shift your weight back onto the right foot, and take a step back with the left foot so that your feet are back in the starting position. Repeat, taking steps with the left foot only.

   Variations/Progressions:
   - With one hand on chair
   - With no hands on chair

© 2017 Brach JS et al. *JAMA Internal Medicine.*

3. Step straight ahead and return alternating feet
   Stand with feet about shoulder width apart. Take a comfortable step straight ahead with your right foot, putting the heel down first and rolling onto the toe. Shift your weight onto the right foot, causing the heel of the left foot to come off the ground. Shift your weight back onto the left foot, and take a step back with the right foot so that your feet are back in the starting position. Now take a comfortable step straight ahead with your left foot, putting the heel down first and rolling onto the toe. Shift your weight onto the left foot, causing the heel of the right foot to come off the ground. Shift your weight back onto the right foot, and take a step back with the left foot so that your feet are back in the starting position. Repeat this pattern, alternating the stepping foot.

   Variations/Progressions:
   - With one hand on chair
   - With no hands on chair

4. Step straight back and return leading with R foot
   Stand with feet about shoulder width apart. Take a comfortable step straight backward with your right foot, putting the toes down first and rolling onto the heel. Shift your weight onto the right foot, causing the toe of the left foot to come off the ground. Shift your weight back onto the left foot, and take a step forward with the right foot so that your feet are back in the starting position. Repeat, taking steps with the right foot only.

   Variations/Progressions:
   - With one hand on chair
   - With no hands on chair

5. Step straight back and return leading with L foot
   Stand with feet about shoulder width apart. Take a comfortable step straight backward with your left foot, putting the toes down first and rolling onto the heel. Shift your weight onto the left foot, causing the toe of the right foot to come off the ground. Shift your weight back onto the right foot, and take a step forward with the left foot so that your feet are back in the starting position. Repeat, taking steps with the left foot only.

   Variations/Progressions:
   - With one hand on chair
   - With no hands on chair

6. Step straight back and return alternating feet
   Stand with feet about shoulder width apart. Take a comfortable step straight backward with your right foot, putting the toes down first and rolling onto the heel. Shift your weight onto the right foot, causing the toe of the left foot to come off the ground. Shift your weight back onto the left foot, and take a step forward with the right foot so that your feet are back in the starting position. Now take a

© 2017 Brach JS et al. *JAMA Internal Medicine.*

comfortable step straight backward with your left foot, putting the toes down first and rolling onto the heel. Shift your weight onto the left foot, causing the toe of the right foot to come off the ground. Shift your weight back onto the right foot, and take a step forward with the left foot so that your feet are back in the starting position. Repeat, alternating the stepping foot.

Variations/Progressions:
- With one hand on chair
- With no hands on chair

7. Step straight back then across forward with R foot (no return step)
Stand with feet about shoulder width apart. Take a comfortable step straight back with your right foot, putting the toe down first and rolling onto the heel. Shift your weight onto your right foot, causing the toes of the left foot to come up off the ground. Begin to shift your weight back onto your left foot, then take a full step forward and across midline with your right foot (past your left foot), putting the heel down first and rolling onto the toe. Shift your weight onto the right foot, causing the heel of the left foot to come up off the ground. Shift the weight back onto the left foot and take a full step back with your right foot (past your left foot). Repeat, taking steps with the right foot only.

Variations/Progressions:
- With one hand on chair
- With no hands on chair

8. Step straight back then across forward with L foot (no return step)
Stand with feet about shoulder width apart. Take a comfortable step straight back with your left foot, putting the toe down first and rolling onto the heel. Shift your weight onto your left foot, causing the toes of the right foot to come up off the ground. Begin to shift your weight back onto your right foot, then take a full step forward and across midline with your left foot (past your right foot), putting the heel down first and rolling onto the toe. Shift your weight onto the left foot, causing the heel of the right foot to come up off the ground. Shift the weight back onto the right foot and take a full step back with your left foot (past your right foot). Repeat, taking steps with the left foot only.

Variations/Progressions:
- With one hand on chair
- With no hands on chair

9. Step across forward and return leading with R foot
Stand with feet about shoulder width apart. With your right foot, take a comfortable step forward and across midline so that your right foot is in front of the left. Shift your weight onto the right foot, causing the heel of the left foot to come up off the ground. Now shift your weight back onto your left foot and step

© 2017 Brach JS et al. *JAMA Internal Medicine.*

back to the starting position with your right foot. Repeat, taking steps with the right foot only.

Variations/Progressions
- With one hand on chair
- With no hands on chair

10. Step across forward and return leading with L foot
Stand with feet about shoulder width apart. With your left foot, take a comfortable step forward and across midline so that your left foot is in front of the right. Shift your weight onto the left foot, causing the heel of the right foot to come up off the ground. Now shift your weight back onto your right foot and step back to the starting position with your left foot. Repeat, taking steps with the left foot only.

Variations/Progressions:
- With one hand on chair
- With no hands on chair

11. Step across forward and return alternating feet
Stand with feet about shoulder width apart. With your right foot, take a comfortable step forward and across midline so that your right foot is in front of the left. Shift your weight onto the right foot, causing the heel of the left foot to come up off the ground. Now shift your weight back onto your left foot and step back to the starting position with your right foot. Now, with your left foot, take a comfortable step forward and across midline so that your left foot is in front of the right. Shift your weight onto the left foot, causing the heel of the right foot to come up off the ground. Now shift your weight back onto your right foot and step back to the starting position with your left foot. Repeat, alternating the stepping foot.

Variations/Progressions:
- With one hand on chair
- With no hands on chair

12. Step straight back with L foot, return, step across forward, return with R foot (diagonal stepping)
Stand with feet about shoulder width apart. With your left foot, take a comfortable step straight backward. Shift your weight onto the left foot, causing the toes of the right foot to come up off the ground. Now shift your weight back onto your right foot and step forward to the starting position with your left foot. With your right foot, take a comfortable step forward and across midline so that your right foot is in front of the left. Shift your weight onto the right foot, causing the heel of the left foot to come up off the ground. Now shift your weight back onto your left foot and step back to the starting position with your right foot. Repeat, stepping backward with the left foot only and forward with the right foot only.

© 2017 Brach JS et al. *JAMA Internal Medicine.*

Variations/Progressions:
- With one hand on chair
- With no hands on chair

13. Step straight back with R foot, return, step across forward with L foot, return (diagonal stepping)
Stand with feet about shoulder width apart. With your right foot, take a comfortable step straight backward. Shift your weight onto the right foot, causing the toes of the left foot to come up off the ground. Now shift your weight back onto your left foot and step forward to the starting position with your right foot. With your left foot, take a comfortable step forward and across midline so that your left foot is in front of the right. Shift your weight onto the left foot, causing the heel of the right foot to come up off the ground. Now shift your weight back onto your right foot and step back to the starting position with your left foot. Repeat, stepping backward with the left foot only and forward with the right foot only.

Variations/Progressions:
- With one hand on chair
- With no hands on chair

14. Partner stepping: Step straight ahead and return with R foot while passing ball
Partner A will stand across from Partner B, approximately arm's length away from each other.
Partner A: Stand with feet about shoulder width apart and ball in hands. With your right foot, take a comfortable step forward while passing the ball to your partner. Shift your weight to the right foot such that the left heel comes up off the ground. Once your partner has the ball, shift your weight onto your left foot and step back to the starting position with your right foot. Now prepare yourself to receive the ball back from your partner.
Partner B: Stand with feet about shoulder width apart. Stay still while your partner steps to pass you the ball. Once you have received the ball, take a comfortable step forward with your right foot while passing the ball back to your partner. Shift your weight to the right foot such that the left heel comes up off the ground. Once your partner has the ball, shift your weight onto your left foot and step back to the starting position with your right foot.

Variations/Progressions:
- Partner A stands diagonally from Partner B. Each participant steps forward and across midline rather than straight ahead while passing the ball.

15. Partner stepping: Step straight ahead and return with L foot while passing ball
Partner A will stand across from Partner B, approximately arm's length away from each other.
Partner A: Stand with feet about shoulder width apart and ball in hands. With your left foot, take a comfortable step forward while passing the ball to your

© 2017 Brach JS et al. *JAMA Internal Medicine.*

partner. Shift your weight to the left foot such that the right heel comes up off the ground. Once your partner has the ball, shift your weight onto your right foot and step back to the starting position with your left foot. Now prepare yourself to receive the ball back from your partner.
Partner B: Stand with feet about shoulder width apart and hands ready to receive the ball. Stay still while your partner steps to pass you the ball. Once you have received the ball, take a comfortable step forward with your left foot while passing the ball back to your partner. Shift your weight to the left foot such that the right heel comes up off the ground. Once your partner has the ball, shift your weight onto your right foot and step back to the starting position with your left foot.

Variations/Progressions:

- Partner A stands diagonally from Partner B. Each participant steps forward and across midline rather than straight ahead while passing the ball.

16. Partner Stepping: Step straight ahead and return with R foot while passing ball/ Step straight back and return with L foot while receiving ball
Partner A will stand across from Partner B, approximately arm's length away from each other.
Partner A: Stand with feet about shoulder width apart and ball in hands. With your right foot, take a comfortable step forward while passing the ball to your partner. Shift your weight to the right foot such that the left heel comes up off the ground. Once your partner has the ball, shift your weight onto your left foot and step back to the starting position with your right foot. Now prepare yourself to receive the ball back from your partner.
Partner B: Stand with feet about shoulder width apart and hands ready to receive ball. As your partner steps toward you with the ball, take a comfortable step back with your left foot and receive the ball. Shift your weight to the left foot such that the toes of the right foot come up off the ground. Once you have received the ball, shift your weight to your right foot and step back to the starting position with your left foot. Now, repeat the pattern with reverse roles.

    Variations/Progressions:

    - Partner A stands diagonally from Partner B. Each participant steps across the midline rather than straight while passing/receiving the ball.

17. Partner Stepping: Step straight ahead and return with L foot while passing ball/Step straight back and return with R foot while receiving ball
Partner A will stand across from Partner B, approximately arm's length away from each other.
Partner A: Stand with feet about shoulder width apart and ball in hands. With your left foot, take a comfortable step forward while passing the ball to your partner. Shift your weight to the left foot such that the right heel comes up off the ground. Once your partner has the ball, shift your weight onto your right foot and

© 2017 Brach JS et al. *JAMA Internal Medicine.*

step back to the starting position with your left foot. Now prepare yourself to receive the ball back from your partner.
Partner B: Stand with feet about shoulder width apart and hands ready to receive ball. As your partner steps toward you with the ball, take a comfortable step back with your right foot and receive the ball. Shift your weight to the left foot such that the toes of the left foot come up off the ground. Once you have received the ball, shift your weight to your left foot and step back to the starting position with your right foot. Now, repeat the pattern with reverse roles.

Variations/Progressions:
- Partner A stands diagonally from Partner B. Each participant steps across across the midline rather than straight while passing/receiving the ball.

18. Line Stepping: Step across forward and return with R foot while passing ball (line 1)/Step across forward and return with L foot while passing ball (line 2)
Half of the class will stand in one line and the other half of the class will stand in the other line. The lines will be facing each other, but the lines should be staggered (no one should be standing directly across from another participant). All balls should start at one end of line 1. The first participant in line 1 will pick up a ball and step forward across with the right foot leading to pass the ball to the first person in line 2. The first person in line 2 will receive the ball while standing still and then step forward across with the left foot to pass the ball to the second person in line 1. The passing will continue in this pattern with each person in line 1 stepping forward and across with his right foot leading and each person in line 2 stepping forward and across with his left foot left leading. Line 1 will step with the left foot and line 2 will step with the right foot when passing balls back down the line in the opposite direction.

Variations/Progressions:
- Each participant in line 1 will step across forward with R foot while passing the ball and step across back with R foot while receiving the ball. Each participant in line 2 will step across forward with L foot while passing the ball and step across back with L foot while receiving ball.

2.4 Strengthening
1. Marching
Start seated in a chair with both feet flat on the ground. Slowly lift one knee in the marching motion, hold it for a few seconds, and lower it back to the ground. Repeat several times on the same side then repeat with the opposite leg.

Progression:
- Increase repetitions/sets completed
- Complete while tapping ball with toes (alternating feet)

2. Ball squeezes (seated hip adduction with ball)

© 2017 Brach JS et al. *JAMA Internal Medicine.*

Started seated in chair with both feet flat on the ground. Place ball in between knees and squeeze ball as if attempting to pop it. Hold for several seconds. Slowly release. Repeat.

Progression:

- Increase hold time
- Increase repetitions/sets

3. March up, out, in, and return (seated hip flexion with abduction)
   Start seated in a chair with both feet flat on the ground. Slowly lift one knee in the marching motion. While keeping the knee lifted, bring knee out to the side and then back into the middle. Repeat several times without putting foot back on the floor then repeat with the opposite leg.

   Progression:
   - Increase repetitions of knee out to the side without putting leg down; increase sets

4. Bilateral knee extension with ball between ankles
   Start seated in chair with feet flat on the ground. Place ball in between ankles and squeeze to hold in place. Slowly extend both legs to lift feet and ball off of the ground, hold it for a few seconds, then slowly bend knees and bring feet and ball back down to ground. Repeat several times.

   Progression:
   - Increase hold time
   - Increase repetitions/sets

5. Hamstring curls with ball under foot
   Start seated in chair with bottom towards the front of the seat and feet flat on the ground. Place ball under one foot. Leaning slightly forward from the trunk and pressing down into ball, slowly roll the ball out until your knee is straight and then roll in back toward you, bending your knee. Repeat several times then repeat with opposite leg.

   Progression:
   - Increase repetitions/sets

6. Mini-squats
   Start standing behind chair with hands resting on chair for support. Keeping heels flat on the ground, bend at the knees and then at the waist as if lowering to sit in a chair. Return to a full standing position. Repeat several times.

   Progression:
   - Increase repetitions/sets

© 2017 Brach JS et al. *JAMA Internal Medicine.*

7. Standing hip abduction
   Start standing behind chair with hands resting on chair for support. Keeping the left foot on the ground and the right knee straight, lift the right leg out to the side and then slowly return it to the starting position. Repeat several times then switch to the other side. Be sure to keep the upper body still throughout this motion.

   Progression:
   - Increase repetitions/sets

8. Standing hip extension
   Start standing behind chair with hands resting on chair for support. Keeping the left foot on the ground and the right knee straight, lift the right heel back towards the wall behind you and then slowly return it to the starting position. Repeat several time then switch to the other side. Be sure to keep the upper body still throughout this motion.

   Progression:
   - Increase repetitions/sets

9. Heel raises
   Start standing behind chair with hands resting on chair for support. Keeping knees straight, press toes into ground while lifting heels off of the ground. Hold in this position for several seconds then slowly lower heels until they are on the ground. Repeat several times.

   Progression:
   - Increase hold time
   - Increase repetitions/sets

10. Repeated chair rise
    Start seated in chair with bottom towards the front of the seat and feet flat on the ground. Lean forward while keeping back flat and stand up from chair. Once you have reached a full standing posture, lean forward and slowly lower yourself back to the starting position. Repeat several times. Complete more sets as able.

    Progression:
    - Increase repetitions/sets
    - Perform while squeezing ball between knees

2.5 Cool-down

1. Seated hamstring stretch
   Start seated in chair with one knee bent so that the foot is flat on the ground and one knee extended so that the heel is on the ground and the toes are sticking up. Keeping your back straight, bend at your waist and reach for the toes that are sticking up. Hold for several seconds then return to the starting position and

© 2017 Brach JS et al. *JAMA Internal Medicine.*

switch legs. Repeat several times on each side, increasing hold time up to 30 seconds as able.

2. Seated figure 4 stretch
   Start seated in chair with both feet flat on the floor. Pick up one foot and cross it over the other knee such that your knee falls out to the side. Gently press down on your knee until a stretch is felt. Hold for several seconds then return to the starting position and switch legs. Repeat several times on each side, increasing hold time up to 30 seconds as able.

3. Standing calf stretch
   Start by standing up behind your chair and holding on to chair with both hands. Put one foot in front of the other, bend the front knee and lean forward while attempting to keep the back heel down (the back knee should remain extended). Once a stretch is felt, hold for several seconds then return to the starting position and switch legs. Repeat several time on each side, increasing hold time up to 30 seconds as able.

4. Seated heel/toe raises
   Start seated in chair with both feet flat on the floor. Keeping toes down, raise heels off the ground. Now, lower the heels to the ground and keep heels down as your raise your toes off of the ground. Repeat several times.

<u>Usual Care Intervention</u>

The Usual Care program consists of 4 component parts: warm-up, cardiovascular exercise, strengthening, and a cool-down. The following provides a short description and rationale for each specific section.

A. Warm-up

1. (approximately 10 minutes, 2 songs) This section uses range of motion exercises for both arms and legs to prepare the cardiovascular and musculoskeletal systems for what is to come in the remainder of the class.

B. Cardiovascular Exercise

1. (approximately 20 minutes, 4 songs) This section consists of exercises for the heart, which will consist of arm and leg movements causing the heart rate to go up. All exercises can be modified to make more or less challenging by performing using arms only, legs only or by using both arms and legs. This portion consists of 3 levels, each progressively more challenging and lasting 4 weeks each.

C. Strengthening

1. (approximately 15-20 minutes, 3 songs) This section consist of a series of exercises for both the arms
and the legs that aim to increase muscular strength. Some of these exercises are completed by sitting in a

© 2017 Brach JS et al. *JAMA Internal Medicine.*

chair or in a supported standing position. A number of exercises in this section require the use of a playground ball.

D. Cool-down/Stretching

1. (approximately 10 minutes, 2 songs) This section consists of a brief set of arm and leg stretches that provide the body with the cues it needs to return to the resting state. During this time, the heart rate and blood pressure should return to a level that is at or close to each participant's pre-exercise level.

Specific Exercises and Description

Safe seated position:

During most of the exercises, it is recommended that the participant start with feet flat on the floor, sitting straight up in the chair with good posture, and back supported. Provide these tips to participants as needed.

A. Warm-up

1. Breathing (3 repetitions)

While reaching upward with both arms take a deep breath, inhale, through the nose. Bring both arms back down, exhale out through mouth.

2.Heel raises (10 repetitions each)

Start seated in chair with both feet flat on the floor. Keeping toes down, raise heels off the ground and then lower the heels to the ground. Repeat several times.

3.Toe raises (10 repetitions each)

Start seated in chair with both feet flat on the floor. Keeping heels down, raise your toes off of the ground and then lower toes back down. Repeat several times.

4.Marching/alternating kicks (10 repetitions each)

Start seated in a chair with both feet flat on the ground. Slowly lift right knee in the marching motion, hold it for a 4 seconds, and lower it back to the ground. Repeat with the left knee. Alternate.

5.Ankle circles (10 repetitions each (5 in each direction clockwise/counterclockwise))

While seated, lift the right leg in the air (just around 2 inches from the floor) and perform a circular motion with the big toe. Pretend that you are drawing a big circle with it. Reverse direction. When you are finished with the right foot, repeat with the left foot. Progress by making the ABC's with your ankle. Using your right ankle, trace the letters of the alphabet from A-Z, imagining your big toe as a writing instrument. Repeat on the other side.

6.Shoulder shrugs (10 repetitions each)

With arms straight and resting at sides (palms facing your torso), elevate the shoulders as high as possible while you inhale. Hold the contraction at the top for a second. Tip: The arms should remain at sides at all times. Only the shoulders should be moving up and down. Lower the shoulders back to the original position during the exhale.

© 2017 Brach JS et al. *JAMA Internal Medicine.*

7.Shoulder circles (10 repetitions each (5 in each direction))
With shoulders relaxed and arms resting loosely at your sides (or in your lap if you're seated), gently roll your shoulders forward, up, back, and down. Reverse direction. You can do this exercise alternating shoulders or both at the same time.

8.Wrist circles (10 repetitions each (5 in each direction))
Begin to rotate both wrists forward in a circular motion. Tip: Pretend that you are trying to draw circles by using your hands as the brush. Reverse direction.

9.Flexion and extension of the hands (4 count hold, 5 repetitions)
Make a fist as tight as possible. Hold for 4 seconds. Open hands, stretching fingers out wide as far as possible. Hold for 4 seconds.

10.Finger taps (5 repetitions down and back)
Touch your thumb to each of your fingertips, one at a time. Make sure that each touch makes an "O" shape. You can perform on one hand or on both at the same time.

11.Neck stretches (5 repetitions total)
Begin with head in neutral position. Rotate your head to the right so that your ear is directly over your right shoulder. Lift head back to neutral position. Rotate your head so that your ear is over your left shoulder. Lift head back to neutral position. Lastly, bring chin toward chest and then back to neutral. Repeat. AVOID NECK EXTENTION. DO NOT look up to the ceiling.

B. Cardiovascular Exercise
Level 1
1.Alternating toe taps forward. Alternating toe taps to the side. Progress to alternating toe taps forward/forward, side/side.

Toe taps forward
While seated, tap right foot out to the front with toes pointed. Bring foot back. Tap left foot out to the front with toes pointed. Bring foot back. Repeat alternating right and left.

Toe taps to the side
While seated, tap right foot out to the right side with toes pointed. Bring foot back to center. Tap left foot out to the left side with toes pointed. Bring foot back. Repeat alternating right and left.

Variations:
*right side only/left side only
*alternating right, then left

2. Alternating heel taps forward. Alternating heel taps to the side. Progress to alternating heel taps forward/forward, side/side.

Heel taps forward
While seated, tap right foot out to the front with heel down. Bring foot back. Tap left foot out to the front with heel down. Bring foot back. Repeat alternating right and left.

Heel taps to the side

While seated, tap right foot out to the right side with heel down. Bring foot back to center. Tap left foot out to the left side with heel down. Bring foot back. Repeat alternating right and left.

Variations:
*right side only/left side only
*alternating right, then left

3. Slow marching with high knee. Progress to tempo.

Marching-high knees

While seated, raise one knee high to the front, picking foot high off of the floor. Lower foot back to the floor. Raise the other knee high to the front, picking foot high off of the floor. Lower back to the floor. Repeat alternating right and left.

Variations:
*right side only/left side only
*alternating right, then left

4. Slow alternating kicks. Progress to tempo.

Kicks

While seated, raise one knee high to the front and kick foot out parallel to the floor. Bend knee and place foot back on floor. Raise other knee high to the front and kick foot out parallel to the floor. Bend knee and place foot back on floor. Repeat alternating right and left.

Variations:
*right side only/left side only
*alternating right, then left

5. Repeat 1-4.

6. Both arms up, elbows out, fingertips touching shoulders. Straight arms reach out to the side slowly, then to tempo. When ready, add marching with high knees slowly and then to tempo.

Variations:
*legs only performing action
*arms only performing action
*for those with history of heart issues, modify movement by keeping arms low below the heart, mimicking arm movement. Start with hands in lap. Then reach down and out to the side with both hands, extending arms. Lastly bring hands back to starting point in the lap

7. Single alternating punches forward. Slow then to tempo. Slowly add kicks, opposite arm punching towards opposite leg. Progress to tempo.

Punching forward

Start with both arms bent at the elbows, close to chest and making a fist. Reach right arm forward and slightly across the body and return back to starting position. Reach left arm forward and slightly across body and return back to starting position.

© 2017 Brach JS et al. *JAMA Internal Medicine.*

Variations:
*legs only performing action
*arms only performing action
*for those with history of heart issues, modify movement by tapping opposite hand on opposite knee with emphasis on moving trunk and shoulder during the movement.

8. Continue punches forward with both arms but change legs to alternating heel taps. Opposite arm punching towards opposite leg. Progress to tempo. Progress again to hopping on heel (which is just quicker heel taps) let arms rest in lap or at side. Hold abdominals in.

Variations:
*continue with heel taps if hopping is too difficult. Drop or add arms when necessary.
*legs only performing action
*arms only performing action
*for those with history of heart issues, modify movement by tapping opposite hand on opposite knee with emphasis on moving trunk and shoulder during the movement.

9. Repeat 6-8.

10. Place paper plates under feet. Perform single heel slides and progress to alternating heel slides forward and back (cross country skiing). When ready, add arms to simulate cross country skiing. Progress again to tempo.

Heel slides forward and back

While seated with paper plates under feet, slide right foot out to the front. Slide foot back. Slide left foot out to the front. Slide foot back. Repeat alternating right and left.

Variations:
*right side only/left side only
*alternating right, then left
*legs only performing action
*arms only performing action
*for those with history of heart issues, modify movement by bringing arms down and tapping the knee with hand, mimicking the cross country skiing.

11. Again with paper plates, perform single heel slides out to the side. When ready, slide both feet out to side and back together. Progress to tempo. Add both arms, reaching up when legs are out and pulling down when legs are together.

Heel slides out to the side

While seated with paper plates under feet, slide right foot out to the right side. Slide foot back to center. Slide left foot out to the left side. Slide foot back to center. Repeat alternating right and left.

Variations:

© 2017 Brach JS et al. *JAMA Internal Medicine.*

*both feet together. When performed together, exercise can be progressed again by crisscrossing the feet when bringing them in together.
*right side only/left side only
*alternating right, then left
*legs only performing action
*arms only performing action
*for those with history of heart issues, modify movement by lowering arms and tapping both knees with hands when legs are out and tapping hips when legs are together.

12. Put plates aside for cool down. Perform alternating heel taps while reaching forward and pulling back with both arms.

Level 2
1.Perform single heel taps out to the front. Come back to starting point with a toe tap (light on feet). Progress by reaching both arms forward with heel taps and pulling back both arms with toe taps. Switch feet. Progress with both arms reaching forward and pulling back with alternating heel taps.

Heel taps forward, Toe taps in
While seated, tap right foot out to the front with heel down. Bring foot back to starting point with a toe tap. Repeat several repetitions on the right, then do the same on the left.

Variations:
*right side only/left side only
*legs only performing action
*arms only performing action
*for those with history of heart issues, modify movement by keeping arms low below the heart, mimicking arm movement. Reach out with both hands, extending arms, to pat the knees with heel taps and pulling back with both arms with toe taps

2. Begin marches. Progress by adding both arms reaching out to the sides and then back in. Slow, then tempo.

Marching
While seated, raise one knee high to the front, picking foot high off of the floor. Lower foot back to the floor. Raise the other knee high to the front, picking foot high off of the floor. Lower back to the floor. Repeat alternating right and left.

Variations:
*right side only/left side only
*alternating right, then left
*legs only performing action
*arms only performing action
*for those with history of heart issues, modify movement by keeping arms low below the heart, mimicking arm movement. Reach out with both

© 2017 Brach JS et al. *JAMA Internal Medicine.*

hands, extending arms, to pat the knees and pulling back with both arms during marching.

3. Perform alternating kicks. Progress with alternating punches forward with opposite arm punching towards opposite leg.

Kicks

While seated, raise one knee high to the front and kick foot out parallel to the floor. Bend knee and place foot back on floor. Raise other knee high to the front and kick foot out parallel to the floor. Bend knee and place foot back on floor. Repeat alternating right and left.

Punching forward

Start with both arms bent at the elbows, close to chest and making a fist. Reach right arm forward and slightly across the body and return back to starting position. Reach left arm forward and slightly across body and return back to starting position.

Variations:

*right side only/left side only
*alternating right, then left
*legs only performing action
*arms only performing action
*for those with history of heart issues, modify movement by tapping opposite hand on opposite knee with the emphasis on moving trunk and shoulder during the movement.

4. Repeat 1-3.

5. Come back to alternating heel taps. Progress with arm bike movement forward.

Heel taps forward

While seated, tap right foot out to the front with heel down. Bring foot back. Tap left foot out to the front with heel down. Bring foot back. Repeat alternating right and left.

Arm bike movement

This exercise is performed by pretending that you are cranking bike pedals in full circles using your hands, the same way you would turn the pedals on a bike with your feet. In this case, your arms should be cranked clockwise, which is forward and away from you or counter clockwise, making big circles or little circles, moving quickly or slowly.

Variations:

*right side only/left side only
*alternating right, then left
*legs only performing action
*arms only performing action
*for those with history of heart issues, modify movement by keeping arms low below the heart, down by abdomen, mimicking arm movement.

© 2017 Brach JS et al. *JAMA Internal Medicine.*

6. Change to alternating toe taps. Progress with arm bike movement moving arms up and down.

Toe taps forward

While seated, tap right foot out to the front with toes pointed. Bring foot back. Tap left foot out to the front with toes pointed. Bring foot back. Repeat alternating right and left.

Arm bike movement up and down

This exercise is performed by pretending that you are cranking bike pedals in full circles using your hands, the same way you would turn the pedals on a bike with your feet. In this case, your arms should be cranked clockwise, which is forward and away from you or counter clockwise. As you are pedaling with your arms, move the arms up toward the head and down toward the lap.

Variations:
*right side only/left side only
*alternating right, then left
*legs only performing action
*arms only performing action
*for those with history of heart issues, modify movement by keeping arms low below the heart, down by abdomen, mimicking arm movement.

7. Change to alternating heel taps forward and coming back to starting point on the toe (light on feet). Progress with both arms reaching forward with heel taps forward and back with toe taps. Add opposite arm punching towards opposite leg.

Variations:
*right side only/left side only
*alternating right, then left
*legs only performing action
*arms only performing action
*for those with history of heart issues, modify movement by keeping arms low below the heart. For the first movement, reach out with both hands, extending arms, to pat the knees with heel tap and pulling back with both arms with toe tap. For the second movement, alternate tapping opposite hand on opposite knee.

8. Repeat 5-7.

9. Place paper plates under feet. Slowly perform alternating heel slides forward and back (cross country skiing). Progress by adding arms to simulate cross country skiing.

Heel slides forward and back

While seated toward the front of the chair, with paper plates under feet, slide right foot out to the front. Slide foot back. Slide left foot out to the front. Slide foot back. Repeat alternating right and left.

Variations:
*right side only/left side only
*alternating right, then left

© 2017 Brach JS et al. *JAMA Internal Medicine.*

*legs only performing action
*arms only performing action
*for those with history of heart issues, modify movement by bringing arms down and tapping the knee with hand, mimicking the cross country skiing.

10. Continue with paper plates under feet. Slide right leg out to the side and slide back in. Progress with opposite arm reaching out and in as leg slides out. Switch to left leg, slide out to side and progress with opposite arm reaching out and in.

Heel slides out to the side

While seated with paper plates under feet, slide right foot out to the right side. Slide foot back to center. Slide left foot out to the left side. Slide foot back to center. Repeat alternating right and left.

Variations:
*right side only/left side only
*alternating right, then left
*legs only performing action
*arms only performing action
*for those with history of heart issues, modify movement by sliding right leg out and tapping with left hand to left knee and bring back as leg comes back.

11. Again with paper plates, slowly slide both feet out to side and back together. Progress to tempo. When ready, add both arms, reaching up when legs are out and pulling down when legs are together. Keep abdominals tight.

Heel slides out to the side

While seated with paper plates under feet, slide right foot out to the right side. Slide foot back to center. Slide left foot out to the left side. Slide foot back to center. Repeat alternating right and left.

Variations:
*right side only/left side only
*alternating right, then left
*legs only performing action
*arms only performing action
*for those with history of heart issues, modify movement by lowering arms and tapping both knees with hands when legs are out and tapping hips when legs are together.

12. To progress again, with paper plate under feet, slide both feet out and in, but when in, crisscross feet and move back out again for 8 repetitions. When ready, add both arms, reaching up when legs are out and pulling down when legs are crisscrossed.

Heel slides out to the side with crisscross

While seated with paper plates under feet, slide both feet out to the side and then back to center, but when in the center, crisscross both feet, alternating left foot in front, then right foot in front. Slide back out again.

Variations:
*legs only performing action

© 2017 Brach JS et al. *JAMA Internal Medicine.*

*arms only performing action
*for those with history of heart issues, modify movement by lowering arms and tapping both knees with hands when legs are out and tapping hips when legs are together.

13. Repeat 9-12 when ready, if not, go to 14.

14. Put plates aside for cool down. Perform alternating heel taps while reaching forward and pulling back with both arms.

Level 3
1. Perform single heel taps out to the front with right leg only. Come back to starting point on toe (light on feet). Progress with both arms reaching forward with heel taps forward and back to starting point with toe taps. Switch heel taps to the left foot only and drop the arms to lap. Progress with both arms reaching forward and pulling back.

Heel taps forward, Toe taps in
While seated, tap right foot out to the front with heel down. Bring foot back to starting point with a toe tap. Repeat several repetitions on the right, then do the same on the left.

Variations:
*right side only/left side only
*legs only performing action
*arms only performing action

2. Begin marches. Progress by adding both arms reaching out to the side and then back in. Slow, then tempo.

Marching
While seated, raise one knee high to the front, picking foot high off of the floor. Lower foot back to the floor. Raise the other knee high to the front, picking foot high off of the floor. Lower back to the floor. Repeat alternating right and left.

Variations:
*right side only/left side only
*alternating right, then left
*legs only performing action
*arms only performing action

3. Clasp hands out in front of body. Make a figure 8, scooping down to hip and sweeping up and over and down to the other side. Stomach tight and inner thighs tight.

Variations:
*if you cannot clasp hands, simply hold arms out in front of body
*if movement is too difficult, participant can simply reach forward and pull back with both arms.

4. Continue to clasp hands out in front of body, but progress to making a rainbow with both arms (side crunches).

Rainbow movement (side crunches)

© 2017 Brach JS et al. *JAMA Internal Medicine.*

While seated with both hands clasped, bring both arms over to the right hip. With both arms reach up and overhead and continue over to the left hip forming a rainbow. Emphasize flexing obliques when arms are coming down to hips. Repeat and make rainbow from left hip to the right hip. Again, emphasize flexing obliques when arms are coming down to hips.

Variations:
*if you cannot clasp hands, simply hold arms out in front of body
*if this movement is too difficult, participant can simply reach forward and pull back with both arms.

5. Repeat 1-4.

6. Come back to alternating heel taps. Progress with arm bike movement forward.

Heel taps forward
While seated, tap right foot out to the front with heel down. Bring foot back. Tap left foot out to the front with heel down. Bring foot back. Repeat alternating right and left.

Arm bike movement
This exercise is performed by pretending that you are cranking bike pedals in full circles using your hands, the same way you would turn the pedals on a bike with your feet. In this case, your arms should be cranked clockwise, which is forward and away from you or counter clockwise, making big circles or little circles, moving quickly or slowly.

Variations:
*right side only/left side only
*alternating right, then left
*legs only performing action
*arms only performing action
*for those with history of heart issues, modify movement by keeping arms low below the heart, down by abdomen, mimicking arm movement.

7. Change to alternating toe taps. Progress with arm bike movement moving arms up and down.

Toe taps forward
While seated, tap right foot out to the front with toes pointed. Bring foot back. Tap left foot out to the front with toes pointed. Bring foot back. Repeat alternating right and left.

Arm bike movement up and down
This exercise is performed by pretending that you are cranking bike pedals in full circles using your hands, the same way you would turn the pedals on a bike with your feet. In this case, your arms should be cranked clockwise, which is forward and away from you or counter clockwise. As you are pedaling with your arms, move the arms up toward the head and down toward the lap.

Variations:
*right side only/left side only

© 2017 Brach JS et al. *JAMA Internal Medicine.*

*alternating right, then left
*legs only performing action
*arms only performing action
*clockwise or counter clockwise

8. Twist side to side while arms punch out and across body. Continue to twist but change to punching across and up. Change twist again by punching across and down. Progress by punching across up/across up and across down/across down. Progress to tempo.

Punching with a twist

Start with both arms bent at the elbows, close to chest and making a fist. As you twist to the right, reach left arm forward and slightly across the body and return back to starting position. As you twist to the left, reach right arm forward and slightly across body and return back to starting position.

Variations:
*if movement is too difficult, participant can simply reach forward and pull back with both arms.
*right side only/left side only
*alternating right, then left

9. Perform alternating kicks. Progress by adding both arms, reaching up and down. Progress to tempo.

Kicks

While seated, raise one knee high to the front and kick foot out parallel to the floor. Bend knee and place foot back on floor. Raise other knee high to the front and kick foot out parallel to the floor. Bend knee and place foot back on floor. Repeat alternating right and left.

Variations:
*right side only/left side only
*alternating right, then left
*legs only performing action
*arms only performing action

10. Repeat 6-9.

11. Place paper plates under feet. Slide right leg out to the side and slide back in. Progress with opposite arm reaching out as leg slides out and in as leg slides back in. Switch to left leg, slide out to side and progress with opposite arm reaching out and in.

Heel slides out to the side

While seated with paper plates under feet, slide right foot out to the right side. Slide foot back to center. Slide left foot out to the left side. Slide foot back to center. Repeat alternating right and left.

Variations:
*right side only/left side only
*alternating right, then left
*legs only performing action
*arms only performing action

© 2017 Brach JS et al. *JAMA Internal Medicine.*

12. With paper plates, continue performing alternating heel slides out to the side. Both arms will reach up (jumping jack) when leg is out to the side. Progress with both legs out to the side and both arms up and both legs and both arms back in (seated jumping jack). Repeat. Put plates aside.

Variations:
*right side only/left side only
*alternating right, then left
*legs only performing action
*arms only performing action

13. Slow high knee marching with a twist.

Marching with a twist

Slow high marches with right leg only. Make goal post with both arms. Twist torso leading left elbow, reaching down and across toward right knee. Repeat several repetitions. Drop arms to sides. Switch marching with left leg only. Make goal post with both arms. Twist torso leading with right elbow reaching down and across toward left knee. Progress to alternating twists.

Variations:
*right side only/left side only
*alternating right, then left
*if movement is too difficult, participant can simply reach forward and pull back with both arms.

14. Alternating heel taps out to the side. Both arms will reach up (jumping jack) when leg is out to the side and return to chest when leg comes back to starting point. Progress with both legs out to the side and both arms up and both legs and both arms back in (seated jumping jack). Repeat.

Variations
*right side only/left side only
*alternating right, then left
*legs only performing action
*arms only performing action

15. Repeat 13, but perform single twists to the right for 8 repetitions then to the left. Alternate twists when ready.

16. Slowly perform alternating heel taps while reaching forward and pulling back with both arms to cool down.

C. Strengthening

1. Bum squeezes

As the name suggests, simply contract (i.e. squeeze) the muscles in your bum (glutes) for as long as you can (5 sec.). Repeat several times.

# of seconds holding:

© 2017 Brach JS et al. *JAMA Internal Medicine.*

# of repetitions completed:
# of sets completed:
Progression:
( ) Increase hold time
( ) Increase repetitions/sets

2.Resisted heel and toe raises (10 repetitions)
Start seated in a chair, bend at waist and rest elbows or hands on knees for added resistance. Perform heel raises by pressing toes into the ground while lifting heels off the ground. Lower heels back to ground. Repeat several times. Change to resisted toes raises. Keep elbows or hands on knees and perform toe raises. Press heels into the ground while lifting toes off the ground. Lower toes back to ground. Repeat several times.
# of repetitions completed:
# of sets completed:
Progression:
( ) Increase repetitions/sets

3.Seated hip abduction (begin with 5 repetitions with a 5 sec. hold and progress to 10 reps)
Start seated in a chair with both feet flat on the ground. Slowly lift one knee in the marching motion. While keeping the knee lifted, bring knee out to the side and hold or pulse for 5 seconds and then bring back into the middle. Repeat several times without putting foot back on the floor then repeat with the opposite leg.
# of seconds holding:
# of repetitions completed:
# of sets completed:
Progression:
( ) Increase repetitions of knee out to the side without putting leg down; increase sets
( ) Increase hold time

4.Seated hip adduction with ball (5 sec. hold) (10 repetitions)
Start seated in chair with both feet flat on the ground. Place ball between knees and squeeze ball as if attempting to pop it. Hold for several seconds. Slowly release. Repeat.
# of seconds holding:
# of repetitions completed:
# of sets completed:
Progression:
( ) Increase repetitions/sets
( ) Increase hold time

5.LAQ with ball (5 sec. hold) (10 repetitions)
Start seated in chair with feet flat on the ground. Place ball in between ankles and squeeze to hold in place. Slowly extend both legs to lift feet and ball off of the ground,

© 2017 Brach JS et al. *JAMA Internal Medicine.*

hold it for a few seconds, then slowly bend knees and bring feet and ball back down to ground. Repeat several times.

# of seconds holding:
# of repetitions completed:
# of sets completed:

Progression:
( ) Increase hold time
( ) Increase repetitions/sets

6.Hamstring curls with ball (10 repetitions each)
Start seated in chair with bottom toward the front of the seat and feet flat on the ground. Place ball under one foot. Pressing down into ball, slowly roll the ball out until your knee is straight and then roll in back toward you, bending your knee. Repeat several times then repeat with opposite leg.

# of repetitions completed:
# of sets completed:

Progression:
( ) Increase repetitions/sets

7.Seated pushups (begin with 5 repetitions and progress to 10)
Start seated in the chair. Place hands on knees. Bring chest down as far as you can to your lap, pause, and push chest away from knees by straightening arms and pressing both hands into lap.
When ready, progress to Chair Push-Ups. Grab the armrests of your chair, elbows bent at a 90-degree angle, and push yourself up, straightening your arms to do minilifts (your bum raises off the seat).

# of repetitions completed:
# of sets completed:

Progression:
( ) Increase repetitions/sets
( ) Chair pushups

8.Front shoulder raises (begin with 5 repetitions and progress to 10 reps with 10 pulses on the last rep).
Sit with a straight torso and arms at the front of your thighs, palms of the hand facing your thighs. This will be your starting position. While maintaining a stationary torso (no swinging), lift the right arm to the front with a slight bend on the elbow and the palms of the hands always facing down. Continue to go up until arm is parallel to the floor. Pause for a second at the top. Lower the right arm back down slowly to the starting position. Finish repetitions before moving on to left arm.

# of repetitions completed:
# of sets completed:

Progression:
( ) Increase repetitions/sets
( ) Perform exercise alternating arms

© 2017 Brach JS et al. *JAMA Internal Medicine.*

( ) Perform exercise with both arms at the same time
( ) Perform exercise with both arms at the same time holding a ball

9.Lateral shoulder raises (begin with 5 repetitions and progress to 10 reps with 10 pulses on the last rep).
Sit with a straight torso and arms by your sides with palms facing you. This will be your starting position. While maintaining the torso in a stationary position (no swinging), lift right arm out to your side with a slight bend on the elbow and the hand slightly tilted forward as if pouring water in a glass. Continue to go up until your arm is parallel to the floor. Pause for a second at the top. Lower the right arm back down slowly to the starting position. Finish repetitions before moving on to left arm.

# of repetitions completed:
# of sets completed:

Progression:
( ) Increase repetitions/sets
( ) Perform exercise alternating arms
( ) Perform exercise with both arms at the same time

10.Rear deltoid flies (begin with 5 repetitions and progress to 10 reps with 10 pulses on the last rep).
While sitting at edge of chair, place feet on floor, bring torso slightly down toward knees. Arms should be resting at sides. This will be your starting position. Raise arms out to side with the hand slightly tilted forward as if pouring water in a glass and elbows slightly bent. Pause for a second at the top. Lower back down slowly to the starting position.

# of repetitions completed:
# of sets completed:

Progression:
( ) Increase repetitions/sets
( ) Perform exercise alternating arms
( ) Perform exercise with both arms at the same time

D. Cool-down/Stretching
1.Seated hamstring stretch (10 sec. hold each side)
Start seated at the edge of the chair with your right leg stretched straight out in front of you and your left leg bent at the knee. Lean forward from your hips and reach for your right ankle until you feel a stretch in your hamstring. Hold for 10 seconds. Repeat on the other side.

2.Seated hip abductor stretch (fig. 4) (10 sec. hold each side)
Sit up straight on chair. Cross your right leg over the top of your left knee (making the #4) and lean forward. Hold for 10 seconds. Repeat on the other side.

3.Overhead triceps stretch (10 sec. hold each side)
Sit up straight, gently lift and reach one arm straight over head. Slowly bend the elbow until your hand is behind its shoulder. Bring your other arm around and slowly and

© 2017 Brach JS et al. *JAMA Internal Medicine.*

gently pull the elbow of the arm being stretched down and back. Hold for the 10 seconds. Repeat on the other side.

4.Cross body shoulder stretch (10 sec. hold each side)
Reach across the chest with one arm. The other arm/hand grasps at the elbow region gently bringing it into your chest or as close as your flexibility will allow. Hold stretch for 10 seconds. Repeat on the other side.

5.Lateral trunk stretch (10 sec. hold each side)
While sitting, stretch one arm over your head and bend in that direction as far as possible. Reach your other hand down toward your heel. Hold for 10 seconds. Repeat on the other side.

6.Trapezius stretch (10 sec. hold each side)
Sit on one hand, palm facing up and sit up straight. This locks in the shoulder so it doesn't move. Look down towards the opposite hip. With your free hand, firmly pull your head towards that hip. Hold for 10 seconds. Repeat on the other side.

7.Upper back and chest stretch (hold for 10 sec. each)
Make a circle with both arms to the front (hands cupping) while rounding back. Bring chin to chest as if looking into a well. Push against opposite hand to stretch. Let go of hands and bring them back to open chest for chest stretch. Hold stretch for 10 sec. Go back to upper back stretch and switch hands. Hold stretch for 10 sec.

8.Neck stretches (3 repetitions)
Sit up straight in a chair. Begin with head in neutral position. Rotate your head to the right so that your ear is directly over your right shoulder. Lift head back to neutral position. Rotate your head so that your ear is over your left shoulder. Lift head back to neutral position. Lastly, bring chin toward chest and then back to neutral. Repeat. AVOID NECK EXTENTION. DO NOT look up to the ceiling.

9.Breathing (3 repetitions)
While reaching upward with both arms take a deep breath, inhale, through the nose. Bring both arms back down, exhale out through mouth.

© 2017 Brach JS et al. *JAMA Internal Medicine.*

eFigure 1a. Detailed CONSORT diagram part 1

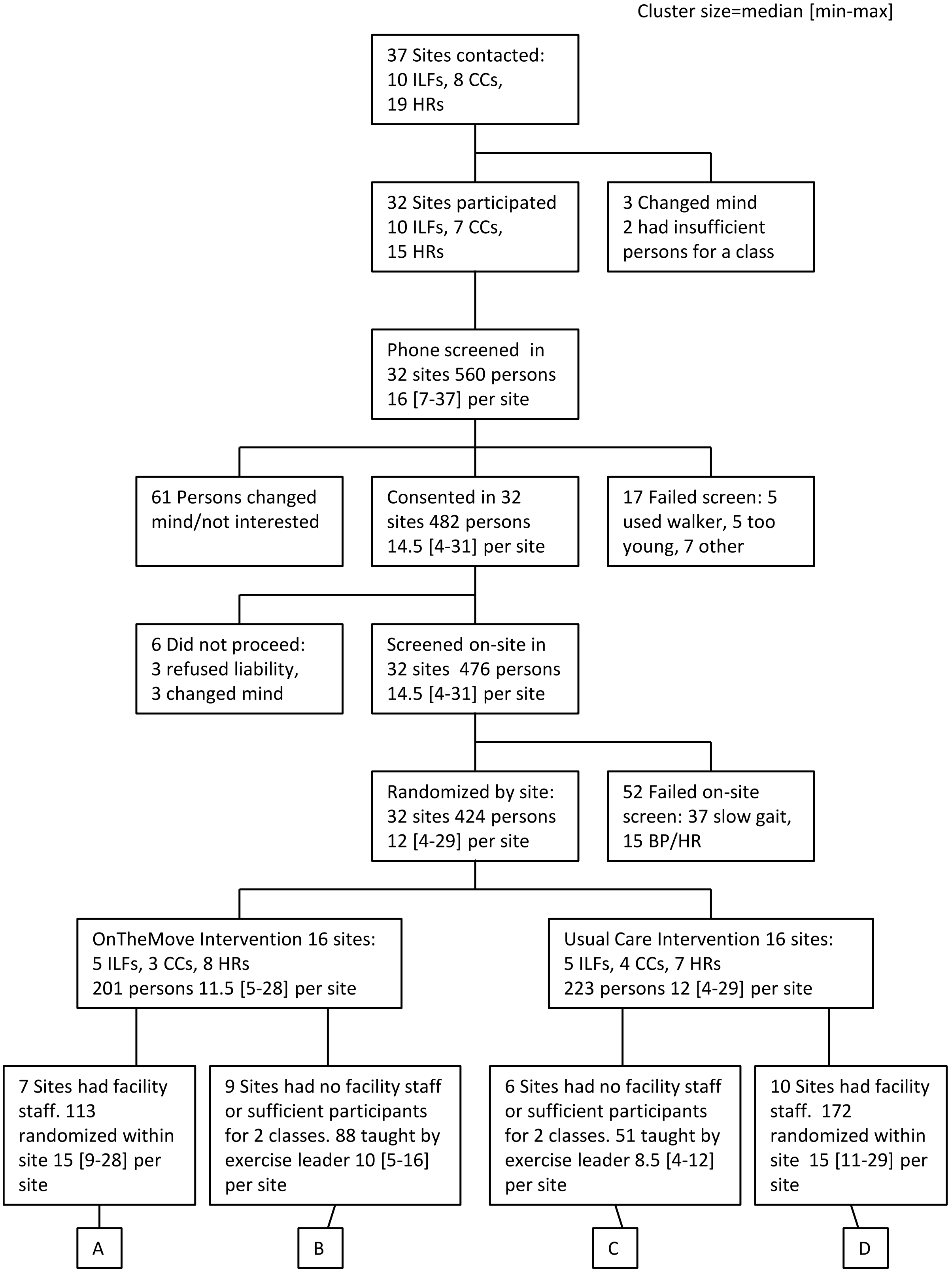

© 2017 Brach JS et al. *JAMA Internal Medicine.*

eFigure 1b. Detailed CONSORT diagram part 2

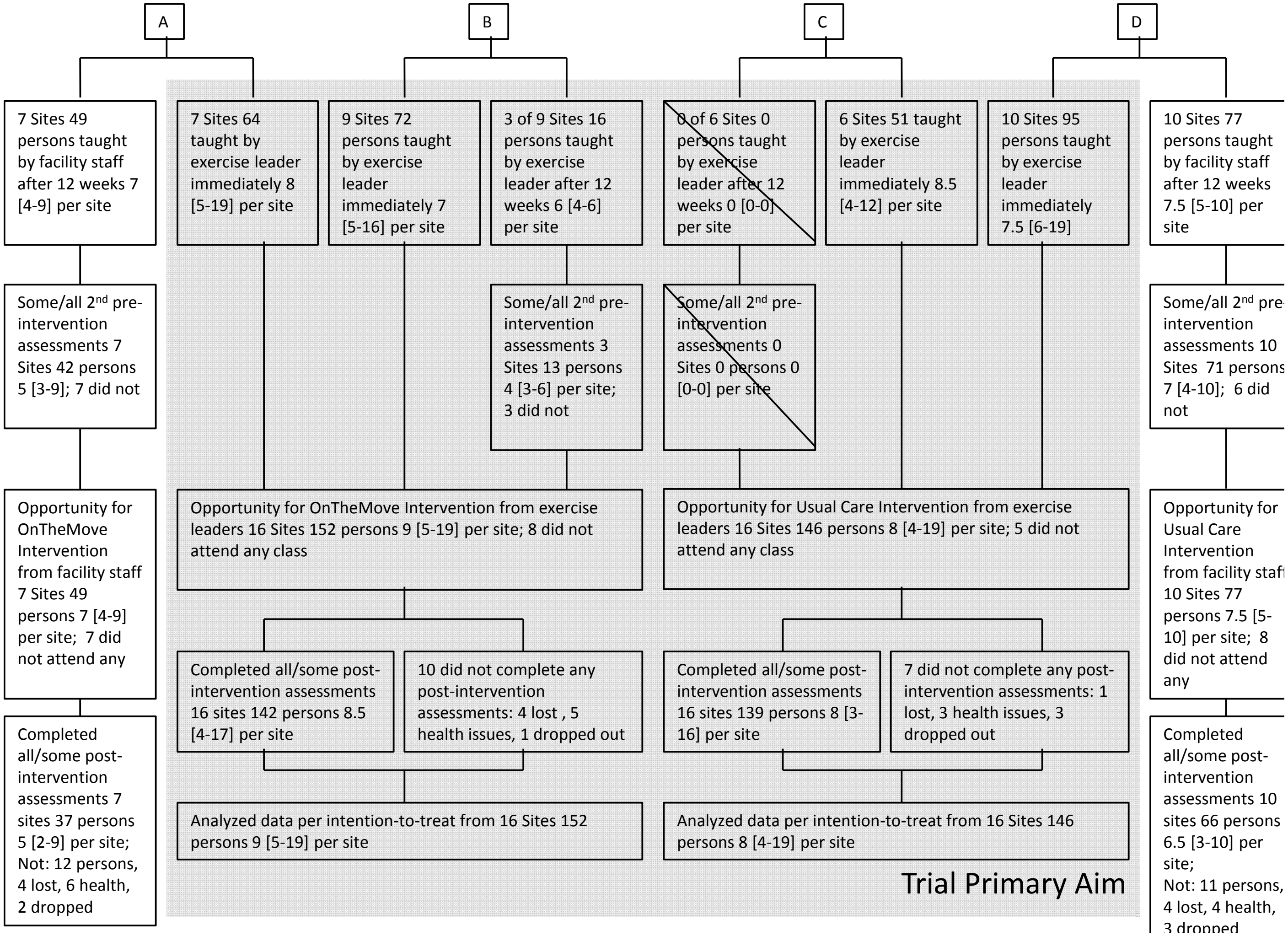

© 2017 Brach JS et al. *JAMA Internal Medicine.*
